# Synthesis, Molecular Docking Study, and Biological Evaluation of New 4-(2,5-Dimethyl-1*H*-pyrrol-1-yl)-*N*’-(2-(substituted)acetyl)benzohydrazides as Dual Enoyl ACP Reductase and DHFR Enzyme Inhibitors

**DOI:** 10.3390/antibiotics12040763

**Published:** 2023-04-16

**Authors:** Mater H. Mahnashi, Pooja Koganole, Prem Kumar S. R., Sami S. Ashgar, Ibrahim Ahmed Shaikh, Shrinivas D. Joshi, Ali S. Alqahtani

**Affiliations:** 1 Department of Pharmaceutical Chemistry, College of Pharmacy, Najran University, Najran 66462, Saudi Arabia; 2 Novel Drug Design and Discovery Laboratory, Department of Pharmaceutical Chemistry, Soniya Education Trust’s College of Pharmacy, Sangolli Rayanna Nagar, Dharwad 580 002, Karnataka, India; 3 Department of Microbiology, Faculty of Medicine, Umm Al-Qura University, Makkah 21955, Saudi Arabia; 4 Department of Pharmacology, College of Pharmacy, Najran University, Najran 66462, Saudi Arabia; 5 Department of Medical Laboratory Sciences, Faculty of Applied Medical Sciences, King Khalid University, Abha 62529, Saudi Arabia

**Keywords:** ADMET studies, antibacterial activity, antitubercular activity, molecular docking, dimethyl pyrrolyl benzohydrazide derivatives

## Abstract

In this study, a new series of 4-(2,5-dimethyl-1*H*-pyrrol-1-yl)-*N′*-(2-(substituted)acetyl) benzohydrazides (**5a–n**) were prepared and new heterocycles underwent thorough characterization and evaluation for antibacterial activity; some of them underwent further testing for in vitro inhibition of enoyl ACP reductase and DHFR enzymes. The majority of the synthesized molecules exhibited appreciable action against DHFR and enoyl ACP reductase enzymes. Some of the synthesized compounds also showed strong antibacterial and antitubercular properties. In order to determine the potential mode of action of the synthesized compounds, a molecular docking investigation was conducted. The results revealed binding interactions with both the dihydrofolate reductase and enoyl ACP reductase active sites. These molecules represent excellent future therapeutic possibilities with potential uses in the biological and medical sciences due to the compounds’ pronounced docking properties and biological activity.

## 1. Introduction

The WHO’s 2022 report lists tuberculosis (TB) as the eighth most common cause of death globally and the most common infectious disease, surpassing HIV/AIDS. The TB-causing bacteria *Mycobacterium tuberculosis* (Mtb) is a slow-growing, acid-fast bacterium with a highly impermeable cell wall. Mtb is an opportunistic pathogen that can remain dormant in macrophages for years before reactivating in people with weakened immune systems, such as those who are also HIV-infected. The rise of extensively drug-resistant (XDR) TB and the expansion of multidrug-resistant (MDR) TB have revived efforts to find new antitubercular drugs. As a result, there is a critical unmet medical need for TB medications that are safer and more potent and that work through various mechanisms [1].

Several medications that include nitrogen heterocycles are now being tested in clinical studies to treat TB [2]. Pyrroles stand out among them as a significant group with strong anticonvulsant [3], antidiabetic [4], antimicrobial [5], and antimycobacterial properties [6,7,8]. Phase IIa clinical studies for the pyrroles-based lead analogue LL3858 are currently taking place in India. LL3858’s MIC values against Mtb were first announced by Lupin Limited in 2004 and ranged from 0.05 to 0.1 µg/mL [9]. Better interaction with enzymes and receptors is likely the result of a structural property of the pyrrole ring with favorable electron properties. This feature has room for additional alteration in the scaffold to achieve the optimal activity profile.

Inhibitors with excellent specificity and the capacity to distinguish between bacterial and host DHFR have found use as antibacterial drugs since bacteria need DHFR to thrive and multiply. These remarkable characteristics make the DHFR enzyme an important target for the development of both antibacterial and antitubercular drugs.

Finding new therapeutic targets relies heavily on academic research, especially with regard to understanding target biology and linkages between targets and disease states. However, research must advance from basic study to the identification and testing of a drug candidate in clinical trials, which are normally carried out by the biopharmaceutical industry, if it is to result in the development of novel pharmaceuticals. Timely attention to target evaluation factors such as target-related safety problems, druggability, and assayability, as well as the possibility for target manipulation to achieve differentiation from current medicines, might help ease this shift [10,11,12].

The goal of this effort was to synthesize some novel pyrrole derivatives with strong antibacterial activity in order to create new therapeutically active units. These compounds were examined for activity against typical infections. The inhibition of the enzymes DHFR and enoyl-ACP reductase was also tested for the active derivatives. Finally, the action target and binding manner were confirmed by molecular docking studies.

## 2. Results and Discussion

**Scheme 1:**
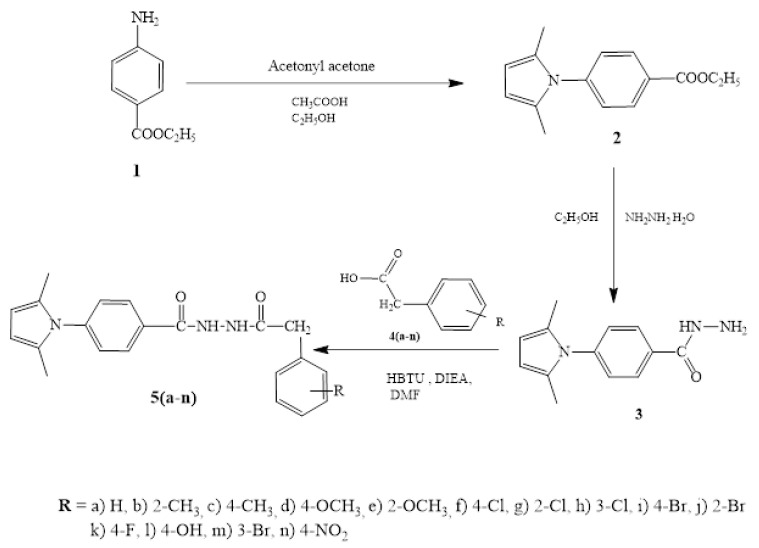
Synthetic route for novel pyrrolyl benzohydrazide derivatives.

The compounds of 4-(2,5-dimethyl-1*H*-pyrrol-1-yl)-*N′*-(2-(substituted)acetyl)benzohydrazides (**5a–n**) were prepared via the reaction of 4-(2,5-dimethyl-1*H*-pyrrol-1-yl)benzohydrazide **3** and substituted phenyl acetic acids in distilled *N,N*-dimethyl formamide using coupling agent 2-(1*H*-benzotriazol-1-yl)-1,1,3,3-tetramethyluronium hexafluorophosphate and *N,N*-diisopropylethylamine as a catalytic reagent under cold conditions (Scheme 1). Structures of the entire novel reported pyrrolyl benzohydrazides were established on the proof of their analytical and spectral information.

In the ^1^H NMR spectrum of compound **5a**, a proton of two –NH appeared as pair of doublets at 10.14 and 9.70 *δ* ppm, and two protons of bridging phenyl-C_3_, C_5_ appeared as a doublet at 7.90 *δ* ppm. Similarly, seven protons of bridging phenyl-C_2,_ C_6_ and phenyl C_2,_ C_3_, C_4,_ C_5,_C_6_ appeared as multiplets at 7.29–7.17 *δ* ppm. The protons of pyrrole-C_3,_ C_4_ appeared as singlet at 5 *δ* ppm, and six methyl protons appeared as singlet at 2.03 *δ* ppm. A singlet signal at 3.65 *δ* ppm proved the presence of the CH_2_ group. The ^13^C NMR of compound **5a** revealed peaks at 169.30 and 164.21 *δ* ppm due to two carbonyl groups separately, and shifts for pyrrole and phenyl carbons appeared in the probable segment between δ ppm of 106.58 to 133.94. Further, the structural confirmation of compound **5a** was attained through a mass spectrum analysis that displayed a peak at *m*/*z* 348.31 (M^+1^), which was equivalent to its molecular weight.

### 2.1. Molecular Docking

To obtain connections and a direction to the active pocket for comparison with other recently synthesized molecules; the 2NSD ligand was re-docked

**Figure 1:**
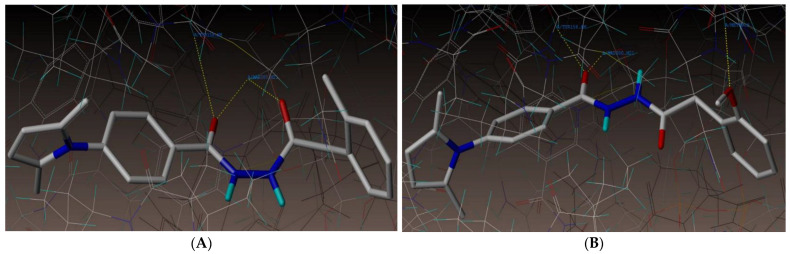
(**A**,**B**) Docked image of molecules **5b** and **5e** in the enzyme’s active site (PDB: 2NSD).

**Figure 2:**
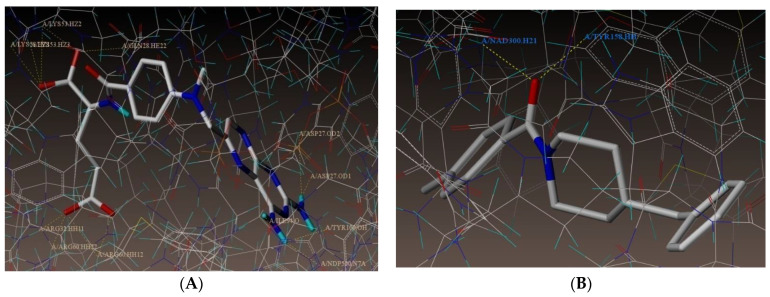
(**A**,**B**) Docked views of methotrexate at the enzyme’s active site in PDB: 1DF7 and 2NSD_ligand at the enzyme’s active site in PDB: 2NSD.

As shown in Figure 1A, compound **5b** had three hydrogen bonding interactions at the enzyme’s active site (PDB ID: 2NSD), two of which were between the oxygen atom of the CO-NH group and the hydrogen atoms of TYR158 and NAD300 (O----H-TYR158, 2.66 Å; O----H-NAD300, 2.33 Å) and one of which was between the oxygen atom of the NHCO group and the hydrogen atom of NAD300 (O----H-NAD300, 2.60 Å). As portrayed in Figure 1B, at the active site of the Inh A enzyme, molecule **5e** demonstrated three hydrogen bonding interactions, including two communications between the oxygen atom of the CO-NH group and the hydrogen atoms of TYR158 and NAD300 (O----H-TYR158, 2.01 Å; O----H-NAD300, 1.59 Å) as well as a residual hydrogen bonding interface between the oxygen atom of the methoxy group and the hydrogen atom of MET98 (O------H-MET98, 2.25 Å). The docked image of the binding interaction of 1DF7_ligand with enzyme active sites shown in Figure 2A reveals 14 bonding connections. The docked image of the 2NSD ligand’s binding contact with the enzyme active sites is shown in Figure 2B. The total docking score (consensus score) for all of the molecules was in the range of 6.73–4.44, representing the summary of all forces of interaction among ligands and the Inh A enzyme. Table 1 is a list of the compounds’ expected binding energies.

Another docking investigation of PDB ID: 1DF7 indicated that all of the molecules had extremely good docking scores against the combination of NADPH and methotrexate and Mycobacterium TB’ dihydrofolate reductase forms. The findings of our study were in line with previously studies, which reported the molecular docking and three-dimensional quantitative structural analysis studies of potent inhibitors of enoyl-acyl carrier protein reductase as possible antimycobacterial agents [13,14].

**Figure 3:**
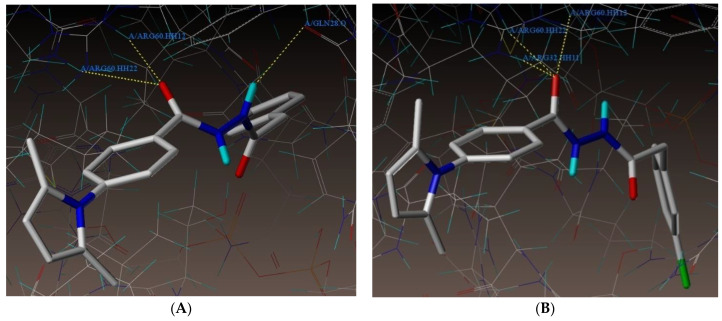
(**A**,**B**) Compounds **5a** and **5h** docked at the enzyme’s active site in PDB: 1DF7.

As shown in Figure 3A, compound **5a** underwent three hydrogen bonding interactions at the enzyme’s active site (PDB ID: 1DF7), two of which were raised by the oxygen of the C=O group of benzohydrazide and involve the hydrogen atoms of ARG60 (-O---H-ARG60, 2.12 Å, 2.54 Å) and hydrogen atom of NH group makes an interaction with oxygen atom of GLN28 (-H----O-GLN28, 2.47 Å). As shown in Figure 3B, at the enzyme’s active site, compound **5h** formed three hydrogen bonds (PDB ID: 1DF7). These bonds were formed by the oxygen atom of the C=O group of benzohydrazide and the hydrogen atoms of ARG60 and ARG32 (-O---H-ARG60, 2.22 Å, 2.07 Å; O---H-ARG32, 2.65 Å). 

**Figure 4:**
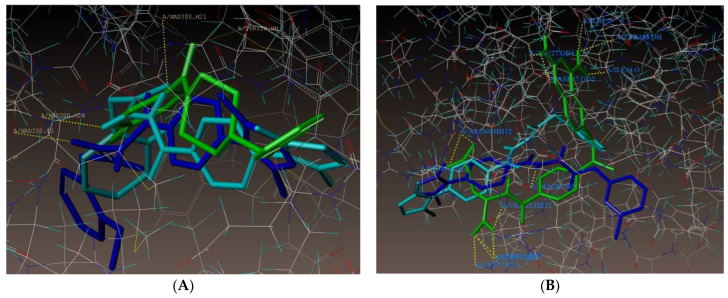
(**A**) Superimposition of the active compounds **5b** (cyan) and **5e** (blue) with corresponding 2NSD_ligand (green); (**B**) superimposition of the active compounds **5a** (cyan) and **5h** (blue) with corresponding 1DF7_ligand (green).

The superimposition of the active compounds **5b** (cyan) and **5e** (blue) with the corresponding 2NSD_ligand (green) and the superimposition of the active compounds **5a** (cyan) and **5h** (blue) with the corresponding 1DF7_ligand (green) are depicted in Figure 4A,B.

All of the compounds demonstrated consensus scores in the range of 6.09 to 4.29 as shown in Table 2, which summarizes all forces of interaction between the ligands and the enzyme. Furthermore, we noticed that the investigated compounds exhibited similar interactions with the amino acid residues (ARG60, ARG32, and GLN28) as the 1DF7 ligand. This implied that molecules connected to enzymes in a manner similar to that of a ligand. These findings corroborated previous studies that reported novel 4-(2,5-dimethylpyrrol-1-yl)/4-pyrrol-1-yl benzoic acid hydrazide analogs and related oxadiazole, triazole, and pyrrole ring systems as a new class of antibacterial, antifungal, and antitubercular medicines [15,16].

### 2.2. Antitubercular and Antibacterial Activities

The results of the MABA method used to test all compounds for antitubercular activity against the *M. tuberculosis* H37Rv strain as well as an antibacterial investigation against *S. aureus* (Gram +ve) and *E. coli* (Gram −ve) are shown in Table 3. The minimum inhibitory concentration (MIC) was used to describe the activities of specific chemicals. In each case, triplicate tests were performed, and the average was taken as the final reading. The molecules displayed good antitubercular activity between 0.8 and 25 µg/mL according to the initial antitubercular analysis. At an MIC of 1.6 µg/mL, compounds **5f**, **5i**, **5j**, and **5n** demonstrated greater action. At an MIC of 0.8 µg/mL, compound **5k** exhibited the highest level of activity. Compounds exhibited antibacterial activity (expressed as MIC) in the range of 0.8 to 100 µg/mL. Previous studies have reported similar findings with novel quinolinone-based thiosemicarbazones and novel analogs of 2,4-disubstituted quinoline derivatives against *M. tuberculosis* [17,18,19].

### 2.3. MtDHFR Inhibitory Activity

Our chemical series’ in vitro MtDHFR inhibitory activity was assessed by monitoring the fluorescence released by MtDHFR substrates when stimulated at 340 nm (Table 3). As the product (NADP+) does not glow, the enzymatic activity was assessed by consuming the enzyme substrate. In this approach, the assay’s positive control (trimethoprim) had an IC_50_ value of 92 μM, which was in agreement with the literature data (88 μM). The majority of the studied molecules were noticeably more effective against MtDHFR (Table 3). The inhibition profiles of 11 of them (**5d–5n**), were stronger than those of trimethoprim.

The various in silico techniques, including molecular docking and the quantitative structure–activity relationship (QSAR), are crucial for obtaining this background information in drug discovery projects [20]. Multidisciplinary professionals from all across the world are working together to find new antimicrobial compounds [21,22]. Bioinformatics researchers have recently concentrated on in silico-designed molecules, enabling the discovery of new molecular targets without the need for an experimental laboratory phase. Researching a compound’s structural characteristics and how it interacts with cellular pathways to either induce or stop a particular human ailment route or pathogen is necessary for compound discovery [23]. For instance, there are few substances that could effectively combat the TB epidemic; as a result, research and the creation of new chemicals are crucial in the fight against TB [24]. By bypassing the manufacture of inert compounds and clinical testing and instead targeting relevant molecules, novel research may lead to the identification of a number of compounds, shortening the time required for new compound discovery [25,26,27].

### 2.4. ADME Studies

The ADME properties of all of the synthesized molecules were determined by using the Swiss ADME web tool, and all of the molecules complied with Lipinski’s rule of five. Swiss ADME studies showed that all of the molecules had good synthetic accessibility, moderate solubility, and GI absorption. Only compounds **5l** and **5n** did not cross BBB. The compounds showed moderate skin permeability in the range of −5.70 to −6.33. The results are shown in Table 4.

The toxicological summary of all of the compounds was determined by using ProTox-II, and the toxic effects are depicted in Table 5. This toxicity data showed that all of the compounds did not exhibit any toxicity.

Additionally, we investigated the potential toxicity of five selected pyrrole derivatives (**5c**, **5d**, **5f**, **5i**, and **5k**) toward the mammalian Vero cell lines and A549 (lung adenocarcinoma) cell lines up to MIC values of 62.5 µg/mL. These tested molecules showed a modest cytotoxicity compared to the standard INH (see Table 6).

## 3. Experimental Section

### 3.1. Chemicals

Chemicals were purchased from Sigma Aldrich, Spectrochem Pvt Ltd., and S. D. Fine Chem. Ltd. for use in the synthesis of the compounds described. Either the recrystallization process or the distillation method was used to purify all of the chemicals and solvents.

### 3.2. Instruments

The melting points of synthesized compounds were calculated using the SHITAL-Digital programmable melting point equipment (SSI -22(B)) and occasionally using a Thiles Tube and were uncorrected; IR spectra were obtained using KBr pellets on a Bruker-T spectrophotometer. Chemical shifts are expressed as δ values (ppm) for the ^1^H NMR and ^13^C NMR measurements, which were performed on Bruker Avance IIINMR500/125 MHz instruments using chloroform (CDCl_3_)/dimethylsulfoxide (DMSO-d6) as the solvent and TMS as the internal standard. The NMR spectra were split into singlet (s), doublet (d), doublet of doublet (dd), triplet (t), quartret (q), and multiplet (m). All of the compounds’ mass spectra on a Water-Q-Tof Premier-HAB213 showed data that were compatible with the projected structure. Analytical thin layer chromatography (TLC) was carried out using Silica Gel GF, and the solvent’s movement was detected using an ultraviolet lamp.

### 3.3. General Procedure for the Synthesis of Dimethylpyrrolylbenzohydrazide Derivatives ***5(a-n)***

In dry DMF (20 mL), 4-(2,5-dimethyl-1*H*-pyrrol-1yl)benzoic acid hydrazide **3** (1.8 mmol) was dissolved. Then, under cold conditions (0–5 °C), substituted phenyl acetic acid (1.9 mmol) was added to it [28]. HBTU (0.87 g, 2.3 mmol) and DIEA (0.93 mL, 5.3 mmol) were added to this mixture and aggressively mixed for 24–30 h at 25 °C. Then, 25% aq NaCl solution was added to quench the reaction, and the mixture was extracted with ethyl acetate (3 × 15 mL). The gathered ethyl acetate extract was washed with saturated Na_2_CO_3_ solution (20 mL) and 1N HCl (15 mL). After that, a 25% aq NaCl solution (10 mL) was added to the process. The combined ethyl acetate extract was concentrated with rotavapor after drying over anhydrous sodium sulphate. The resulting residue was purified using column chromatography by eluting with ethyl acetate:petroleum ether (6:4).

All related spectra are provided in the Supplementary Materials.

#### 3.3.1. Synthesis of (**5a**): 4-(2,5-Dimethyl-1H-pyrrol-1-yl)-N′-(2-phenylacetyl)benzohydrazide

Light brown amorphous solid. (Yield 87%). M.p106–108 °C; FTIR (KBr-cm^−1^): 3245 (NH), 3025 (NH), 2926 (Ar-C=CH), 1679 (C=O), 1647 (C=O).

^1^H NMR (CDCl_3_,500 MHz, δ ppm): 10.14 (d, 1H, *J* = 8.2, CONH), 9.70 (d, 1H, *J* = 8.2, NHCO), 7.90-7.88 (d, 2H, bridging phenyl-C_3_, C_5_-H), 7.29-7.17 (m, 7H, bridging phenyl-C_2_, C_6_-H, phenyl-C_2_, C_3_, C_4_, C_5_, C_6_ -H), 5.89 (s, 2 H, pyrrole-C_3_, C_4_-H), 3.65 (s, 2H, CH_2_), 2.03 (s, 6H, 2CH_3_).

^13^C NMR (CDCl_3_, 125 MHz, δ): 169.30 (-NHCO), 164.21 (-CONH), 133.94 (phenyl-C_1_), 128.61 (bridging phenyl-C_2_, C_6_) 128.39 (bridging phenyl-C_4_) 128.51 (phenyl-C_2_, C_5_), 128.25 (pyrrole-C_2_, C_5_), 127.38 (bridging phenyl-C_3_, C_5_), 127.31 (phenyl-C_4_), 106.58 (pyrrole-C_3_, C_4_), 41.09(-CH_2_), 15.25 (2CH_3_).

Mass (*m*/*z*): found 348.3158 (M^+^ + 1); Calcd. 347.42.

CHN Anal. Of C_21_H_21_N_3_O_2_: Calcd.C, 72.60; H, 6.09; N, 12.10; Found: C, 72.58; H, 6.08; N, 12.09.

#### 3.3.2. Synthesis of (**5b**): 4-(2, 5-Dimethyl-1H-pyrrol-1-yl)-N′-(2-(o-tolyl)acetyl)benzohydrazide

Light brown amorphous solid. (Yield 76%). M.p109–112 °C; FTIR (KBr-cm^−1^): 3232 (NH), 2922 (Ar-C=CH), 1618 (C=O), 1570 (C=C).

^1^H NMR (CDCl_3_, 500 MHz, δ ppm): 9.97 (d, 1H, *J* = 8.2, CONH), 8.96 (d, 1H, *J* = 8.2, NHCO), 7.88-7.86 (d, 2H, *J* = 8.2, bridging phenyl-C_3_, C_5_-H), 7.25-7.10 (m, 6H, bridging phenyl-C_2_, C_6_-H, phenyl-C_3_, C_4_, C_5_, C_6_ -H), 5.89 (s, 2H, pyrrole-C_3_, C_4_-H), 3.67 (s, 2H, CH_2_), 2.32 (s, 3H, CH_3_), 1.98 (s, 6H, 2CH_3_).

^13^C NMR (CDCl_3_, 125 MHz, δ): 168.94 (-NHCO), 163.98 (-CONH), 142.66 (bridging phenyl-C_1_), 137.21 (phenyl-C_2_), 131.91 (phenyl-C_1_), 130.85 (phenyl-C_3_), 130.34 (phenyl-C_4_), 130.22 (bridging phenyl-C_2_, C_6_), 128.56 (bridging phenyl-C_3_, C_5_), 128.33 (bridging phenyl-C_4_), 128.28 (phenyl-C_6_), 126.63 (phenyl-C_5_), 128.09 (pyrrole-C_2_, C_5_), 106.59 (pyrrole-C_3_, C_4_), 39.45 (-CH_2_), 19.58 (CH_3_), 13.06 (2CH_3_).

Mass (*m*/*z*): found 362.3349 (M^+^ + 1); Calcd. 361.45.

CHN Anal. Of C_22_H_23_N_3_O_2_: Calcd.C, 73.11; H, 6.41; N, 11.63; Found: C, 73.09; H, 6.40; N, 11.64.

#### 3.3.3. Synthesis of (**5c**): 4-(2,5-Dimethyl -1H-pyrrol-1-yl)-N′-(2-(p-tolyl)acetyl)benzohydrazide

Light brown amorphous solid. (Yield 65%). M.p95–98 °C; FTIR (KBr-cm^−1^): 3422 (NH), 3259 (NH), 2922 (Ar-C=CH), 1625 (C=O), 1493 (C=C).

^1^H NMR (CDCl_3_, 500 MHz, δ ppm):10.03 (d, 1H, CONH), 9.45 (d, 1H, *J* = 8.2, NHCO), 7.90 (d, 2H, *J* = 8.2, bridging phenyl-C_3_, C_5_-H), 7.25-7.10 (m, 6H, bridging phenyl-C_2_, C_6_-H, phenyl-C_3_, C_4_, C_5_, C_6_-H), 5.89 (s, 2H, pyrrole-C_3_, C_4_-H), 3.60 (s, 2H, -CH_2_), 2.30 (s,3H, CH_3_), 1.98 (s, 6H, 2CH_3_).

^13^C NMR (CDCl_3_, 125 MHz, δ): 168.55 (-NHCO), 163.59 (-CONH), 142.71 (bridging phenyl-C_1_), 137.41 (phenyl-C_4_), 130.31 (phenyl-C_1_), 129.77 (bridging phenyl-C_2_, C_6_), 129.23 (phenyl-C_2_, C_6_, C_3,_ C_5_), 128.58 (bridging phenyl-C_3_, C_5_), 128.42 (bridging phenyl-C_4_), 128.21 (pyrrole-C_2_, C_5_), 106.59 (pyrrole-C_3_, C_4_), 38.62 (-CH_2_), 21.10 (CH_3_), 13.05 (2CH_3_).

Mass (*m*/*z*): found 362.3388 (M^+^ + 1); Calcd. 361.45.

CHN Anal. Of C_22_H_23_N_3_O_2_: Calcd.C, 73.11; H, 6.41; N, 11.63; Found: C, 73.10; H, 6.41; N, 11.62.

#### 3.3.4. Synthesis of (**5d**): 4-(2,5-Dimethyl-1H-pyrrol-1-yl)-N′-(2-(4-methoxyphenyl)acetyl)benzohydrazide

Light brown amorphous solid. (Yield 78%). M.p118–120 °C; FTIR (KBr-cm^−1^): 3422 (NH), 3259 (NH), 29223(Ar-C=CH), 16259 (C=O), 1493 (C=C).

^1^H NMR (CDCl_3_, 500 MHz, δ ppm):10.04 (d, 1H, *J* = 8.2, CONH). 9.47 (d, 1H, *J* = 8.2, NHCO), 7.91 (d, 2H, *J* = 8.2, bridging phenyl-C_3_, C_5_-H), 7.25-7.17 (m, 4H, bridging phenyl-C_2_, C_6_-H, phenyl-C_2_, C_6_-H), 6.83 (d, 2H, phenyl-C_3_, C_5_-H), 5.89 (s, 2H, pyrrole-C_3_, C_4_-H), 3.74 (s, 3H, OCH_3_), 3.58 (s, 2H, CH_2_), 1.98 (s, 6H, 2CH_3_).

^13^C NMR (CDCl_3_, 125 MHz, δ): 169.21 (-NHCO), 163.87 (-CONH), 142.68 (bridging phenyl-C_1_), 159.04 (phenyl-C_4_), 130.41 (phenyl-C_1_), 130.24 (phenyl-C_2,_ C_6_), 128.54 (bridging phenyl-C_2_, C_6_), 128.35 (bridging phenyl-C_3_, C_5_), 128.29 (pyrrole-C_2_, C_5_), 114.40 (phenyl-C_3,_ C_5_), 125.3 (bridging phenyl-C_4_), 106.61 (pyrrole-C_3_, C_4_), 55.26 (OCH_3_), 40.44 (-CH_2_), 13.05 (2CH_3_).

Mass (*m*/*z*): found 378.3364 (M^+^ + 1); Calcd. 377.44.

CHN Anal. Of C_22_H_23_N_3_O_3_: Calcd.C, 70.01; H, 6.14; N, 11.13; Found: C, 70.04; H, 6.13; N, 11.12.

#### 3.3.5. Synthesis of (**5e**): 4-(2,5-Dimethyl-1H-pyrrol-1-yl)-N′-(2-(2-methoxyphenyl)acetyl)benzohydrazide

Light brown amorphous solid. (Yield 85%). M.p138–142 °C; FTIR (KBr-cm^−1^): 3180 (NH), 3026(NH), 2970 (Ar-C=CH), 1606 (C=O).

^1^H NMR (CDCl_3_, 500 MHz, δ ppm):9.78 (d, 1H, *J* = 8.2, CONH). 9.18 (d, 1H, *J* = 8.2, NHCO), 7.88 (d, 2H, *J* = 8.2, bridging phenyl-C_3_, C_5_-H), 7.29-6.91 (m, 6H, bridging phenyl-C_2_, C_6_-H, phenyl-C_2_, C_3_, C_4_, C_5_-H), 5.89 (s, 2H, pyrrole-C_3_, C_4_-H), 3.90 (s, 3H, OCH_3_), 3.67(s, 2H, CH_2_), 2.03 (s, 6H, 2CH_3_).

^13^C NMR (CDCl_3_, 125 MHz, δ): 168.32 (-NHCO), 163.11 (-CONH), 157.04 (phenyl-C_2_), 142.50 (bridging phenyl-C_1_),131.17 (phenyl-C_6_),130.17 (bridging phenyl-C_4_), 129.23 (bridging phenyl-C_2_, C_6_), 128.58 (pyrrole-C_2_, C_5_), 128.33 (phenyl-C_4_), 128.19 (bridging phenyl-C_3_, C_5_), 122.27 (phenyl-C_1_), 122.19 (phenyl-C_5_), 110.83 (phenyl-C_3_), 106.52 (pyrrole-C_3_, C_4_), 55.62 (OCH_3_), 36.83 (-CH_2_), 13.05 (2CH_3_).

Mass (*m*/*z*): found 378.2687 (M^+^ + 1); 379.2685 (M^+^ + 1); Calcd. 377.44

CHN Anal. Of C_22_H_23_N_3_O_3_: Calcd.C, 70.01; H, 6.14; N, 11.13; Found: C, 70.02; H, 6.12; N, 11.11.

#### 3.3.6. Synthesis of (**5f**): N′-(2-(4-Chlorophenyl)acetyl)-4-(2,5-dimethyl-1H-pyrrol-1-yl)benzohydrazide

Light brown amorphous solid. (Yield 67%). M.p130–132 °C; FTIR (KBr-cm^−1^): 3227 (NH), 3029 (NH), 2923 (Ar-C=CH), 1695 (C=O), 1620 (C=O).

^1^H NMR (DMSO-*d*_6_, 500 MHz, δ ppm):10.23 (d, 1H, *J* = 8.2, CONH). 10.04 (d, 1H, *J* = 8.2, NHCO), 7.92 (d, 2H, *J* = 8.2, bridging phenyl-C_3_, C_5_-H), 7.25-7.16 (m, 6H, bridging phenyl-C_2_, C_6_-H, phenyl-C_2_, C_3_, C_5_, C_6_-H), 5.89 (s, 2H, pyrrole-C_3_, C_4_-H), 3.57 (s, 2H, CH_2_), 1.97 (s, 6H, 2CH_3_).

^13^C NMR (CDCl_3_, 125 MHz, δ): 168.32 (-NHCO), 163.99 (-CONH), 142.88 (bridging phenyl-C_1_), 133.53 (phenyl-C_4_), 132.18 (phenyl-C_1_),130.59 (phenyl-C_2,_ C_6_), 130.04 (bridging phenyl-C_2_, C_6_), 129.03 (phenyl-C_3,_ C_5_), 128.52 (bridging phenyl-C_3_, C_5_), 128.44 (bridging phenyl-C_4_), 128.27 (pyrrole-C_2_, C_5_), 106.71 (pyrrole-C_3_, C_4_), 40.39 (-CH_2_), 13.06 (2CH_3_).

Mass (*m*/*z*): found 382.2198 (M^+^ + 1), 384.2190 (M^+^ + 3); Calcd. 381.86.

CHN Anal. Of C_21_H_20_ClN_3_O_2_: Calcd.C, 70.01; H, 6.14; N, 11.13; Found: C, 70.04; H, 6.13; N, 11.12.

#### 3.3.7. Synthesis of (**5g**): N′-(2-(2-Chlorophenyl)acetyl)-4-(2,5-dimethyl-1H-pyrrol-1-yl)benzohydrazide

Light brown amorphous solid. (Yield 70%). M.p145–147 °C; FTIR (KBr-cm^−1^): 3222 (NH), 3031 (NH), 2923 (Ar-C=CH), 1695 (C=O), 1611 (C=O).

^1^H NMR (CDCl_3_, 500 MHz, δ ppm): 10.38 (d, 1H, *J* = 8.2, CONH), 9.77 (d, 1H, *J* = 8.2, NHCO), 7.92 (d, 2H, *J* = 8.2, bridging phenyl-C_3_, C_5_-H), 7.31-7.13 (m, 6H, bridging phenyl-C_2_, C_6_-H, phenyl-C_2,_ C_3_, C_4_, C_5_-H), 5.88 (s, 2H, pyrrole-C_3_, C_4_-H), 3.78 (s, 2H, CH_2_), 1.97 (s, 6H, 2CH_3_).

^13^C NMR (CDCl_3_, 125 MHz, δ): 167.72 (-NHCO), 163.86 (-CONH), 142.64 (bridging phenyl-C_1_), 134.51 (phenyl-C_1_), 131.88 (phenyl-C_6_), 131.59 (bridging phenyl-C_4_), 130.17 (bridging phenyl-C_2_, C_6_), 129.73 (phenyl-C_3_), 129.12 (phenyl-C_2_), 128.55 (pyrrole-C_2_, C_5_), 128.36 (phenyl-C_4_), 128.30 (bridging phenyl-C_3_, C_5_), 127.27 (phenyl-C_5_), 106.59 (pyrrole-C_3_, C_4_), 38.96 (-CH_2_), 13.06 (2CH_3_).

Mass (*m*/*z*): found 382.2158 (M^+^ + 1), 383.21.50 (M^+^ + 2); Calcd. 381.86.

CHN Anal. Of C_21_H_20_ClN_3_O_2_: Calcd.C, 66.05; H, 5.28; N, 11.00; Found: C, 66.02; H, 5.26; N, 11.02.

#### 3.3.8. Synthesis of (**5h**): N′-(2-(3-Chlorophenyl)acetyl)-4-(2,5-dimethyl-1H-pyrrol-1-yl)benzohydrazide

Light brown amorphous solid. (Yield 55%). M.p104–108 °C; FTIR (KBr-cm^−1^): 3450 (NH), 3026 (NH), 2922 (Ar-C=CH), 1606 (C=O).

^1^H NMR (CDCl_3_, 500 MHz, δ ppm):10.29 (d, 1H, *J* = 8.2, CONH), 10.20 (d, 1H, *J* = 8.2, NHCO), 7.93 (d, 2H, *J* = 8.2, bridging phenyl-C_3_, C_5_-H), 7.30-7.12 (m,6H, bridging phenyl-C_2_, C_6_-H, phenyl-C_2_, C_4_, C_5_, C_6_-H), 5.88 (s, 2H, pyrrole-C_3_, C_4_-H), 3.59 (s, 2H, CH_2_), 2.03 (s, 6H, 2CH_3_).

^13^C NMR (CDCl_3_, 125 MHz, δ): 167.88 (-NHCO), 163.88 (-CONH), 142.85 (bridging phenyl-C_1_), 134.71 (phenyl-C_1_), 134.61 (phenyl-C_3_), 130.08 (phenyl-C_5_), 129.37 (bridging phenyl-C_2_, C_6_), 128.55 (bridging phenyl-C_3_, C_5_), 128.45 (pyrrole-C_2_, C_5_), 128.28 (bridging phenyl-C_4_), 127.71 (phenyl-C_4,_ C_6_), 127.44 (phenyl-C_2_), 106.67 (pyrrole-C_3_, C_4_), 40.60 (-CH_2_), 13.06 (2CH_3_).

Mass (*m*/*z*): found 382.2158 (M^+^ + 1); Calcd. 381.86.

CHN Anal. Of C_21_H_20_ClN_3_O_2_: Calcd.C, 66.05; H, 5.28; N, 11.00; Found: C, 66.07; H, 5.29; N, 11.03.

#### 3.3.9. Synthesis of (**5i**): N′-(2-(4-Bromophenyl)acetyl)-4-(2,5-dimethyl-1H-pyrrol-1-yl)benzohydrazide

Brown amorphous solid. (Yield 72%). M.p100–104 °C; FTIR (KBr-cm^−1^): 3450 (NH), 2922 (Ar-C=CH), 1622 (C=O).

^1^H NMR (CDCl_3_, 500 MHz, δ ppm): 10.06 (d, 1H, *J* = 8.2, CONH). 9.41 (d, 1H, *J* = 8.2, NHCO), 7.95 (d, 2H, *J* = 8.2, bridging phenyl-C_3_, C_5_-H), 7.52-7.21 (m, 4H, bridging phenyl-C_2_, C_6_-H, phenyl-C_2_, C_6_-H), 7.14 (d, 2H, phenyl-C_3_, C_5_-H), 5.89 (s, 2H, pyrrole-C_3_, C_4_-H), 3.82 (s, 2H, CH_2_), 1.98 (s, 6H, 2CH_3_).

^13^C NMR (DMSO-*d*_6_, 125 MHz, δ): 168.98 (-NHCO), 164.75 (-CONH), 141.20 (bridging phenyl-C_1_), 135.03 (phenyl-C_1_), 131.76 (phenyl-C_3,_ C_5_), 131.25 (phenyl-C_2,_ C_6_), 128.38 (bridging phenyl-C_2_, C_6_), 128.25 (bridging phenyl-C_3_, C_5_), 127.87 (bridging phenyl-C_4_),127.43 (pyrrole-C_2_, C_5_), 119.67 (phenyl-C_4_), 106.38 (pyrrole-C_3_, C_4_), 38.92 (-CH_2_), 12.77 (2CH_3_).

Mass (*m*/*z*): found 426.1733 (M^+^), 428.1699 (M^+^ + 2); Calcd. 426.31.

CHN Anal. Of C_21_H_20_BrN_3_O_2_: Calcd.C, 59.17; H, 4.73; N, 9.86; Found: C, 59.10; H, 4.72; N, 9.84.

#### 3.3.10. Synthesis of (**5j**): N′-(2-(2-Bromophenyl)acetyl)-4-(2,5-dimethyl-1H-pyrrol-1-yl)benzohydrazide

Light brown amorphous solid. (Yield 84%). M.p123–125 °C; FTIR (KBr-cm^−1^): 3445 (NH), 2987 (Ar-C=CH), 1611 (C=O).

^1^H NMR (CDCl_3_, 500 MHz, δ ppm):10.06 (d, 1H, *J* = 8.2, CONH). 9.40 (d, 1H, *J* = 8.2, NHCO), 7.91 (d, 2H, *J* = 8.2, bridging phenyl-C_3_, C_5_-H), 7.53-7.09 (m, 6H, bridging phenyl-C_2_, C_6_-H, phenyl-C_3_, C_4_, C_5_, C_6_-H), 5.89 (s, 2H, pyrrole-C_3_, C_4_-H), 3.82 (s, 2H, CH_2_), 1.98 (s, 6H, 2CH_3_).

^13^C NMR (DMSO-*d*_6_, 125 MHz, δ): 167.27 (-NHCO), 164.75 (-CONH), 142.67 (bridging phenyl-C_1_), 133.55 (phenyl-C_1_), 133.11 (phenyl-C_3_), 130.24 (phenyl-C_4_), 129.40 (bridging phenyl-C_2_, C_6_), 1129.26 (phenyl-C_5_), 128.58 (phenyl-C_6_), 128.38 (bridging phenyl-C_3_, C_5_), 128.30 (bridging phenyl-C_4_), 127.99 (pyrrole-C_2_, C_5_), 125.00(phenyl-C_2_), 106.58 (pyrrole-C_3_, C_4_), 41.62 (-CH_2_), 13.07 (2CH_3_).

Mass (*m*/*z*): found 426.1691 (M^+^ + 1), 428.1699 (M^+^ + 2); Calcd. 426.31.

CHN Anal. Of C_21_H_20_BrN_3_O_2_: Calcd.C, 59.17; H, 4.73; N, 9.86; Found: C, 59.15; H, 4.72; N, 9.87.

#### 3.3.11. Synthesis of (**5k**): 4-(2, 5-Dimethyl-1H-pyrrol-1-yl)-N′-(2-(4-flurophenyl)acetyl)benzohydrazide

Light brown amorphous solid. (Yield 73%). M.p134–137 °C; FTIR (KBr-cm^−1^): 3448 (NH), 2937 (Ar-C=CH), 1646 (C=O).

^1^H NMR (CDCl_3_, 400 MHz, δ ppm): 10.50 (s, 1H, CONH). 10.25 (s, 1H, NHCO), 9.24 (s, 1H, OH), 8.01-7.15 (m, 8H, bridging phenyl-C_2_, C_3_, C_5,_ C_6_-H, phenyl-C_2_, C_3_, C_5_, C_6_-H), 5.83 (s, 2H, pyrrole-C_3_, C_4_-H), 3.57 (s, 2H, CH_2_), 1.99 (s, 6H, 2CH_3_).

^13^C NMR (DMSO-*d*_6_, 125 MHz, δ): 168.71 (-NHCO), 163.83 (-CONH), 161.53 (phenyl-C_4_), 143.24 (bridging phenyl-C_1_), 131.81 (phenyl-C_1_), 131.57 (phenyl-C_2_), 130.19 (phenyl-C_6_), 129.73 (bridging phenyl-C_2_, C_6_), 129.32 (pyrrole-C_2_, C_5_), 128.37 (bridging phenyl-C_3_, C_5_), 128.32 (bridging phenyl-C_4_), 116.51 (phenyl-C_3,_ C_5_), 106.50 (pyrrole-C_3_, C_4_), 38.76 (-CH_2_), 12.96 (2CH_3_).

Mass (*m*/*z*): found 366.2451 (M^+^ + 1), 367.2485 (M^+^ + 2); Calcd. 365.41.

CHN Anal. Of C_21_H_20_FN_3_O_2_: Calcd.C, 69.03; H, 5.52; N, 11.50; Found: C, 69.01; H, 5.51; N, 11.49.

#### 3.3.12. Synthesis of (**5l**): 4-(2, 5-Dimethyl-1H-pyrrol-1-yl)-N′-(2-(4-hydroxyphenyl)acetyl)benzohydrazide

Light brown amorphous solid. (Yield 65%). M.p 190–193 °C; FTIR (KBr-cm^−1^): 3446 (NH, OH), 2922 (Ar-C=CH), 1643 (C=O).

^1^H NMR (CDCl_3_, 500 MHz, δ ppm): 10.45 (s, 1H, CONH). 10.14 (s, 1H, NHCO), 9.24 (s, 1H, OH), 8.02 (d, 2H, *J* = 8.2, bridging phenyl-C_3_, C_5_-H), 7.39-6.71 (m, 6H, bridging phenyl-C_2_, C_6_-H, phenyl-C_2_, C_3_, C_5_, C_6_-H), 5.83 (s, 2H, pyrrole-C_3_, C_4_-H), 3.42 (s, 2H, CH_2_), 1.99 (s, 6H, 2CH_3_).

^13^C NMR (DMSO-*d*_6_, 125 MHz, δ): 169.11 (-NHCO), 167.85 (-CONH), 163.83 (phenyl-C_4_), 142.85 (bridging phenyl-C_1_), 134.64 (phenyl-C_2,_ C_6_), 129.37 (bridging phenyl-C_4_), 128.50 (bridging phenyl-C_2_, C_6_), 128.49 (bridging phenyl-C_3_, C_5_), 128.28 (phenyl-C_1_), 127.74 (pyrrole-C_2_, C_5_), 115.64 (phenyl-C_3,_ C_5_), 105.65 (pyrrole-C_3_, C_4_), 39.63 (-CH_2_), 13.01 (2CH_3_).

Mass (*m*/*z*): found 364.3542 (M^+^ + 1), Calcd. 363.42.

CHN Anal. Of C_21_H_21_N_3_O_3_: Calcd.C, 69.41; H, 5.82; N, 11.56; Found: C, 69.40; H, 5.81; N, 11.55.

#### 3.3.13. Synthesis of (**5m**): 4-(2, 5-Dimethyl-1H-pyrrol-1-yl)-N′-(2-(3-bromophenyl)acetyl)benzohydrazide

Light brown amorphous solid. (Yield 57%). M.p 143–149 °C; FTIR (KBr-cm^−1^): 3447 (NH), 2924 (Ar-C=CH), 1641 (C=O).

^1^H NMR (CDCl_3_, 500 MHz, δ ppm): 10.30 (s, 1H, CONH). 10.04 (s, 1H, NHCO), 7.99 (d, 2H, *J* = 8.2, bridging phenyl-C_3_, C_5_-H), 7.25-7.16 (m, 6H, bridging phenyl-C_2_, C_6_-H, phenyl-C_2_, C_4_, C_5_, C_6_-H), 5.99 (s, 2H, pyrrole-C_3_, C_4_-H), 3.55 (s, 2H, CH_2_), 1.97 (s, 6H, 2CH_3_).

^13^C NMR (DMSO-*d*_6_, 125 MHz, δ): 169.25 (-NHCO), 167.21 (-CONH), 143.63 (bridging phenyl-C_1_), 134.05 (phenyl-C_1_), 133.13 (phenyl-C_4_), 131.62 (phenyl-C_5_), 130.24 (phenyl-C_2_), 129.06 (bridging phenyl-C_2_, C_6_), 128.88 (pyrrole-C_2_, C_5_), 128.58 (bridging phenyl-C_4_), 128.28 (phenyl- C_6_), 127.79 (bridging phenyl-C_3_, C_5_), 125.06 (phenyl-C_3_), 106.10 (pyrrole-C_3_, C_4_), 40.62 (-CH_2_), 13.03 (2CH_3_)

Mass (*m*/*z*): found 426.1225 (M^+^ + 1), Calcd. 425.07.

CHN Anal. Of C_21_H_20_BrN_3_O_2_: Calcd.C, 59.17; H, 4.73; N, 9.86; Found: C, 59.15; H, 4.72; N, 9.84.

#### 3.3.14. Synthesis of (**5n**): 4-(2, 5-Dimethyl-1H-pyrrol-1-yl)-N′-(2-(4-nitrophenyl)acetyl)benzohydrazide

Light brown amorphous solid. (Yield 78%). M.p 172–173 °C; FTIR (KBr-cm^−1^): 3252 (NH), 1703 (C=O), 1640 (C=O).

^1^H NMR (DMSO-*d*_6_, 500 MHz, δ ppm): 10.45 (s, 1H, CONH). 10.14 (s, 1H, NHCO), 8.01 (d, 2H, *J* = 8.2, bridging phenyl-C_3_, C_5_-H), 7.39 (d, 2H, phenyl-C_3_, C_5_-H), 7.15 (d, 2H, bridging phenyl-C_2_, C_6_-H), 6.72 (phenyl-C_2_, C_6_-H), 5.83 (s, 2H, pyrrole-C_3_, C_4_-H), 3.47 (s, 2H, CH_2_), 1.98 (s, 6H, 2CH_3_).

^13^C NMR (DMSO-*d*_6_, 125 MHz, δ): 169.20 (-NHCO), 164.86 (-CONH), 148.65 (phenyl-C_4_), 146.69 (bridging phenyl-C_1_), 142.63 (phenyl-C_1_), 130.34 (phenyl-C_2,_ C_6_), 128.24 (bridging phenyl-C_4_), 128.35 (bridging phenyl-C_2_, C_6_), 128.29 (pyrrole-C_2_, C_5_), 126.63 (bridging phenyl-C_3_, C_5_), 125.43 (phenyl-C_3,_ C_5_), 106.61 (pyrrole-C_3_, C_4_), 30.34 (-CH_2_), 13.05 (2CH_3_).

Mass (*m*/*z*): found 493.4536 (M^+^ + 1), Calcd. 392.42

CHN Anal. Of C_21_H_20_N_4_O_4_: Calcd.C, 64.28; H, 5.14; N, 14.28; Found: C, 64.26; H, 5.13; N, 14.26.

### 3.4. Molecular Docking Using Surflex-Dock

Utilizing the patented Sybyl-X 2.0 search engine, Surflex-Dock was employed for molecular docking to clarify the binding mechanism of chemicals at the active sites of the ENR enzyme and dihydrofolate reductase enzyme [29]. This provided clear information for future molecule structural optimization. Using the Brookhaven Protein Database (PDB http://www.rcsb.org/pdb), the crystal structures of enoyl acyl carrier protein reductase InhA in association with N-(4-methylbenzoyl)-4-benzylpiperidine (PDB ID 2NSD, 1.9 Å X-ray resolution) and dihydrofolate reductase of *Mycobacterium tuberculosis* complexed with NADPH and methotrexate were obtained. The ligands and protein used in our docking technique were produced in accordance with the Sybyl-X 2.0 standard protocol [30,31]. All of the hydrogen atoms were added to define the correct configuration and tautomeric states. Then, the modeled structure was energy-minimized using a Tripos force field with a distance-dependent dielectric function, partial atomic charges were calculated by using the AMBER7F9902 method, and finally water molecules were removed from the model. The geometry of the molecule CP was subsequently optimized to minimal energy using the Powell energy minimization algorithm and Tripos force field with Gasteiger-Hückel charges. The CP was then separately docked into the binding pocket for docking–scoring analysis. To identify the ligand–protein interactions, the top pose and protein were loaded into work area and the Molecular Computer Aided Design (MOLCAD) program was employed to visualize the binding mode between the protein and ligand.

### 3.5. ADMET Studies

ProTox-II was used to predict toxicities (the results are presented in Table 5), and Molecular ADME properties were calculated using the in silico Swiss ADME online program [32] (the results are presented in Table 4).

### 3.6. MTT-Based Cytotoxicity Activity

Cellular conversion of MTT (3-(4,5-dimethylthiazo-2-yl)-2,5-diphenyl-tetrazolium bromide) into a formazan product was performed to evaluate the cytotoxic activity (IC_50_) of some of the compounds against A549 (lung adenocarcinoma) MV cell lines up to a concentration of 50 mg/mL using a Promega Cell Titer 96 non-radioactive cell proliferation assay with cisplatin as the positive control. The IC_50_ values given in Table 6 are the averages ± SEM of three independent measurements.

### 3.7. Antitubercular Activity

All of the newly synthesized compounds were evaluated against *M. tuberculosis* strain H_37_Rv by means of a Microplate Alamar Blue assay (MABA). The results are reported in Table 3 with MIC values [33].

### 3.8. Antibacterial Activity

An antibacterial inhibition study of all molecules was conducted in comparison with ciprofloxacin as the reference drug against *S. aureus* (Gram +ve) and *E. coli* (Gram-ve) using a broth microdilution method [34,35]. Antibacterial activity data are given in Table 3 along with their MIC values.

## 4. Conclusions

All of the 14 newly synthesized pyrrolyl benzohydrazide derivatives exhibited moderate to good antitubercular potency with MICs that ranged from 0.8 to 25 µg/mL. All of the derivatives were also analyzed in a molecular docking study, and these reported novel inhibitors fitted closely with the binding site of the ENR enzyme and DHFR enzyme in a manner similar to that of the 2NSD_ligand and 1DF7_ligand. Compounds **5f**, **5i,** and **5k** showed promising enzyme inhibitory activity (on both the enzymes) during the in vitro assay. Due to the size of the present novel molecules used to minimize *M. tuberculosis* growth, additional modification of molecular fragment changes through molecular modeling and 3D-QSAR studies might be considered as new leads for the development of druggable candidates.

## Figures and Tables

**Table 1:** Surflex docking score for pyrrole derivatives on the PDB ID: 2NSD in kcal/mol.

Compounds	Total Score ^a^	Crash Score ^b^	Polar Score ^c^	D Score ^d^	PMF Score ^e^	G Score ^f^	Chem Score ^g^
2NSD_ligand	9.25	−0.93	1.54	−150.083	−63.091	−250.959	−46.922
**5m**	6.73	−1.45	0.00	−154.008	−47.31	−254.69	−35.77
**5l**	6.66	−1.40	0.00	−150.09	−49.357	−243.120	−33.421
**5b**	6.64	−1.40	0.00	−150.813	−67.397	−275.03	−38.55
**5h**	6.60	−1.32	0.02	−152.344	−47.406	−251.930	−35.59
**5d**	6.58	−1.43	0.00	−152.248	−151.700	−253.195	−35.191
**5a**	5.77	−1.94	0.00	−147.496	−48.955	−259.388	−34.870
**5e**	5.57	−2.26	2.20	−152.202	−81.681	−249.402	−43.879
**5k**	5.51	−350	1.09	−121.605	−70.322	−254.814	−32.838
**5i**	5.33	−202	0.40	−148.863	−52.545	−248.550	−36.981
**5n**	5.23	−161	0.78	−109.058	−49.383	−220.034	−30.758
**5c**	5.09	−1.90	0.67	−115.009	−60.352	−228.411	−29.464
**5g**	4.64	−1.53	0.00	−158.311	−42.321	−254.298	−35.355
**5j**	4.52	−3.58	1.75	−137.888	−69.052	−268.596	−40.945
**5f**	4.44	−3.21	1.79	−160.561	−68.462	−260.375	−41.004

^a^ C score (consensus score) integrates a number of popular scoring functions for ranking the affinity of ligands bound to the active site of a receptor and reports the output of total score. ^b^ Crash score revealing the inappropriate penetration into the binding site. Crash scores close to 0 are favorable. Negative numbers indicate penetration. ^c^ Polar score indicating the contribution of the polar interactions to the total score. The polar score may be useful for excluding docking results that make no hydrogen bonds. ^d^ D score for charge and van der Waals interactions between the protein and the ligand. ^e^ PMF score indicating the Helmholtz free energies of interactions for protein–ligand atom pairs (potential of mean force, PMF). ^f^ G score showing hydrogen bonding, complex (ligand–protein), and internal (ligand–ligand) energies. ^g^ Chem-score points for H-bonding, lipophilic contact, and rotational entropy, along with an intercept term.

**Table 2:** Surflex docking score for pyrrole derivatives on PDB ID 1DF7 in kcal/mol.

Compounds	C Score ^a^	Crash Score ^b^	Polar Score ^c^	D Score ^d^	PMF Score ^e^	G Score ^f^	Chem Score ^g^
Ligand	13.76	−1.32	8.92	−229.875	−138.104	−353.514	−38.494
**5i**	6.09	−0.80	1.24	−74.154	−46.492	−203.793	−23.832
**5j**	6.07	−1.60	1.12	−140.353	−40.058	−212.293	−30.808
**5n**	5.87	−1.06	5.71	−65.226	−50.507	−138.374	−31.331
**5e**	5.76	−1.66	1.01	−108.460	−32.896	−206.830	−29.186
**5b**	5.72	−1.92	1.13	−135.734	−48.681	−242.893	−31.229
**5a**	5.68	−1.82	1.78	−76.626	−51.678	−205.484	−26.926
**5f**	5.52	−1.05	0.34	−79.996	−40.690	−202.029	−25.188
**5c**	5.50	−2.09	1.09	−117.301	−42.304	−215.813	−29.720
**5k**	5.40	−2.17	1.08	−116.983	−44.793	−218.472	−29.380
**5m**	4.86	−1.06	0.00	−85.927	−64.808	−211.509	−26.216
**5g**	4.84	−1.73	0.00	−92.589	−53.332	−243.476	−28.948
**5h**	4.68	−1.01	2.52	−59.255	−62.042	−146.737	−24.631
**5d**	4.55	−2.34	0.66	−117.427	−47.229	−225.918	−27.805
**5i**	4.29	−1.31	2.78	−62.632	−41.743	−139.089	−23.044

^a^ C score (consensus score) integrates a number of popular scoring functions for ranking the affinity of ligands bound to the active site of a receptor and reports the output of total score. ^b^ Crash score revealing the inappropriate penetration into the binding site. Crash scores close to 0 are favorable. Negative numbers indicate penetration. ^c^ Polar score indicating the contribution of the polar interactions to the total score. The polar score may be useful for excluding docking results that make no hydrogen bonds. ^d^ D score for charge and van der Waals interactions be-tween the protein and the ligand. ^e^ PMF score indicating the Helmholtz free energies of interactions for protein–ligand atom pairs (potential of mean force, PMF). ^f^ G score showing hydrogen bonding, complex (ligand–protein), and internal (ligand–ligand) energies. ^g^ Chem-score points for H-bonding, lipophilic contact, and rotational entropy, along with an intercept term.

**Table 3:** Preliminary in vitro antitubercular results, antibacterial activity results, MtDHFR inhibition activity, and enoyl ACP reductase inhibition values (results are expressed as % InhA inhibition) of newly synthesized pyrrole derivatives.

Compound	*M. tuberculosis* H_37_Rv MIC Values in µg mL^−1^ (µmol mL^−1^)	*S. aureus* (Gram +ve)	*E. coli* (Gram –ve)	IC_50_ (µM) MtDHFR	% Inhibition of InhA at 50 µM
		MIC in µg mL^−1^ (µmol mL^−1^)	MIC in µg mL^−1^ (µmol mL^−1^)		
**5a**	25 (71.95)	50 (143.91)	50 (143.91)	146	-
**5b**	25 (69.16)	0.8 (2.21)	50 (138.33)	125	-
**5c**	25 (69.16)	3.12(8.63)	50 (138.33)	118	20
**5d**	3.25 (8.61)	3.12 (8.2)	100 (264.94)	85	32
**5e**	6.25 (16.55)	6.25 (16.55)	100 (264.94)	78	-
**5f**	1.6 (4.19)	1.6 (4.19)	50 (130.93)	25	76
**5g**	3.25 (8.51)	3.12 (8.17)	50 (130.93)	26	-
**5h**	3.25 (8.51)	6.25 (16.36)	50 (130.93)	30	-
**5i**	1.6 (3.75)	6.25 (14.66)	50 (117.28)	18	72
**5j**	1.6 (3.75)	6.25 (14.66)	50 (117.28)	15	-
**5k**	0.8 (2.18)	1.6 (4.37)	50 (136.41)	10	88
**5l**	3.25 (8.94)	6.25 (17.19)	50 (137.58)	30	-
**5m**	3.25 (7.64)	12.5 (29.40)	50 (117.62)	18	-
**5n**	1.6 (4.07)	12.5 (31.85)	50 (127.41)	15	-
Pyrazinamide	3.125	-	-		-
Streptomycin	6.25	-	-		-
Ciprofloxacin	-	2	2		-
TMP	-	-	-	92	-
TCL	-	-	-	-	>99

**Table 4:** Swiss ADME web tool’s ADME properties for synthetic molecules.

Compound	Log P	Molar Refractivity	TPSA	HBA	HBD	RB	GI Absorption	BBB Permeant	Log Kp cm/s	Solubility	CYP Inhibitor					Lipinski Violation	Synthetic Accessibility
											1A2	2C19	2C9	2D6	3A4		
**5a**	2.62	100.99	63.13	2	2	7	High	Yes	−5.93	Moderately soluble	No	Yes	Yes	Yes	Yes	0	2.65
**5b**	2.80	105.96	63.13	2	2	7	High	Yes	−5.76	Moderately soluble	No	Yes	Yes	Yes	Yes	0	2.81
**5c**	3.09	105.96	63.13	2	2	7	High	Yes	−5.76	Moderately soluble	No	No	Yes	Yes	Yes	0	2.76
**5d**	3.03	107.48	72.36	3	2	8	High	Yes	−6.14	Moderately soluble	No	Yes	Yes	Yes	Yes	0	2.74
**5e**	3.02	107.48	72.36	3	2	8	High	Yes	−6.14	Moderately soluble	No	Yes	Yes	Yes	Yes	0	2.82
**5f**	2.96	106.00	63.13	2	2	7	High	Yes	−5.70	Moderately soluble	Yes	Yes	Yes	Yes	Yes	0	2.70
**5g**	3.35	106.00	63.13	2	2	7	High	Yes	−5.70	Moderately soluble	Yes	Yes	Yes	Yes	Yes	0	2.77
**5h**	3.16	106.00	63.13	2	2	7	High	Yes	−5.70	Moderately soluble	Yes	Yes	Yes	Yes	Yes	0	2.70
**5i**	3.16	108.69	63.13	2	2	7	High	Yes	−5.93	Moderately soluble	Yes	Yes	Yes	Yes	Yes	0	2.75
**5j**	3.40	108.69	63.13	2	2	7	High	Yes	−5.93	Moderately soluble	Yes	Yes	Yes	Yes	Yes	0	2.83
**5k**	2.71	100.95	63.13	3	2	7	High	Yes	−5.97	Moderately soluble	No	No	Yes	Yes	Yes	0	2.69
**5l**	2.63	103.02	83.36	3	3	7	High	No	−6.28	Moderately soluble	Yes	No	Yes	Yes	No	0	2.64
**5m**	3.11	108.69	63.13	2	2	7	High	Yes	−5.93	Moderately soluble	Yes	Yes	Yes	Yes	Yes	0	2.83
**5n**	2.48	109.81	108.95	4	2	8	High	No	−6.33	Moderately soluble	No	Yes	Yes	No	Yes	0	2.90

**Table 5:** Toxicity studies of synthesized compounds.

Compound Code	LD50 mg/kg	Hepatotoxicity	Carcinogenicity	Immunotoxicity	Mutagenicity	Cytotoxicity	Aryl Hydrocarbon Receptor	Androgen Receptor (AR)	Androgen Receptor Ligand Binding Domain	Aromatase	Estrogen Receptor Ligand Binding Domain	Peroxisome Proliferator Activated Receptor Gamma	Nuclear Factor	Heat Shock Factor Response Element	Mitochondrial Membrane Potential	Phosphoprotein	ATPase Family AAA Domain Containing Protein 5
**5a**	150	Active	Active	Inactive	Inactive	Inactive	Inactive	Inactive	Inactive	Inactive	Inactive	Inactive	Inactive	Inactive	Inactive	Inactive	Inactive
**5b**	150	Active	Active	Inactive	Inactive	Inactive	Inactive	Inactive	Inactive	Inactive	Inactive	Inactive	Inactive	Inactive	Inactive	Inactive	Inactive
**5c**	150	Active	Active	Inactive	Inactive	Inactive	Inactive	Inactive	Inactive	Inactive	Inactive	Inactive	Inactive	Inactive	Inactive	Inactive	Inactive
**5d**	150	Active	Inactive	Inactive	Inactive	Inactive	Inactive	Inactive	Inactive	Inactive	Inactive	Inactive	Inactive	Inactive	Inactive	Inactive	Inactive
**5e**	700	Active	Active	Inactive	Inactive	Inactive	Inactive	Inactive	Inactive	Inactive	Inactive	Inactive	Inactive	Inactive	Inactive	Inactive	Inactive
**5f**	700	Active	Active	Inactive	Inactive	Inactive	Inactive	Inactive	Inactive	Inactive	Inactive	Inactive	Inactive	Inactive	Inactive	Inactive	Inactive
**5g**	150	Active	Inactive	Inactive	Inactive	Inactive	Inactive	Inactive	Inactive	Inactive	Inactive	Inactive	Inactive	Inactive	Inactive	Inactive	Inactive
**5h**	150	Active	Inactive	Inactive	Inactive	Inactive	Inactive	Inactive	Inactive	Inactive	Inactive	Inactive	Inactive	Inactive	Inactive	Inactive	Inactive
**5i**	150	Active	Inactive	Inactive	Inactive	Inactive	Inactive	Inactive	Inactive	Inactive	Inactive	Inactive	Inactive	Inactive	Inactive	Inactive	Inactive
**5j**	150	Active	Inactive	Inactive	Inactive	Inactive	Inactive	Inactive	Inactive	Inactive	Inactive	Inactive	Inactive	Inactive	Inactive	Inactive	Inactive
**5k**	150	Active	Active	Inactive	Active	Inactive	Inactive	Inactive	Inactive	Inactive	Inactive	Inactive	Inactive	Inactive	Inactive	Inactive	Inactive
**5l**	150	Active	Active	Inactive	Inactive	Inactive	Inactive	Inactive	Inactive	Inactive	Inactive	Inactive	Inactive	Inactive	Inactive	Inactive	Inactive
**5m**	150	Active	Inactive	Inactive	Inactive	Inactive	Inactive	Inactive	Inactive	Inactive	Inactive	Inactive	Inactive	Inactive	Inactive	Inactive	Inactive
**5n**	150	Active	Active	Inactive	Active	Inactive	Inactive	Inactive	Inactive	Inactive	Inactive	Inactive	Inactive	Inactive	Inactive	Inactive	Inactive

**Table 6:** In vitro evaluation of MTT-based cytotoxicity activity of selected compounds against human lung cancer cell lines A549 and MV cell line (IC_50_ in µg/mL).

Compound	IC50 (µM) ^a^	
	MV Cell Lines ^b^	A549 ^c^
**5a**	-	-
**5b**	-	
**5c**	221 ± 0.8	219 ± 0.7
**5d**	214 ± 0.6	214 ± 0.2
**5e**	-	-
**5f**	215 ± 0.2	216 ± 0.5
**5g**	-	-
**5h**	-	-
**5i**	218 ± 0.3	214 ± 0.4
**5j**		
**5k**	221 ± 0.3	219 ± 0.4
**5l**	-	-
**5m**	-	-
**5n**	-	-
INH	>450	>450

^a^ Cytotoxicity is expressed as IC_50_, which is the concentration of compound reduced by 50% of the optical density of treated cells with respect to untreated cells using an MTT assay. Values are the means ± SEM of three independent experiments. ^b^ Mammalian Vero cell lines (NCCS-Pune, India). ^c^ A549 (lung adenocarcinoma) cell lines (NCCS-Pune, India).

## Data Availability

The data used to support the findings of this study are available from the corresponding author upon request.

## Supplementary Materials

The following supporting information can be downloaded at: https://www.mdpi.com/article/10.3390/antibiotics12040763/s1.
