# Supplementary material for: Synthesis, Molecular Docking Study, and Biological Evaluation of New 4-(2,5-Dimethyl-1H-pyrrol-1-yl)-N’-(2-(substituted)acetyl)benzohydrazides as Dual Enoyl ACP Reductase and DHFR Enzyme Inhibitors
Source: Antibiotics (Basel). 2023 Apr 16;12(4):763. doi: 10.3390/antibiotics12040763 (PMC10135272; doi:10.3390/antibiotics12040763)

SPECTRUM NO:01 (IR spectrum of Compound 5a)

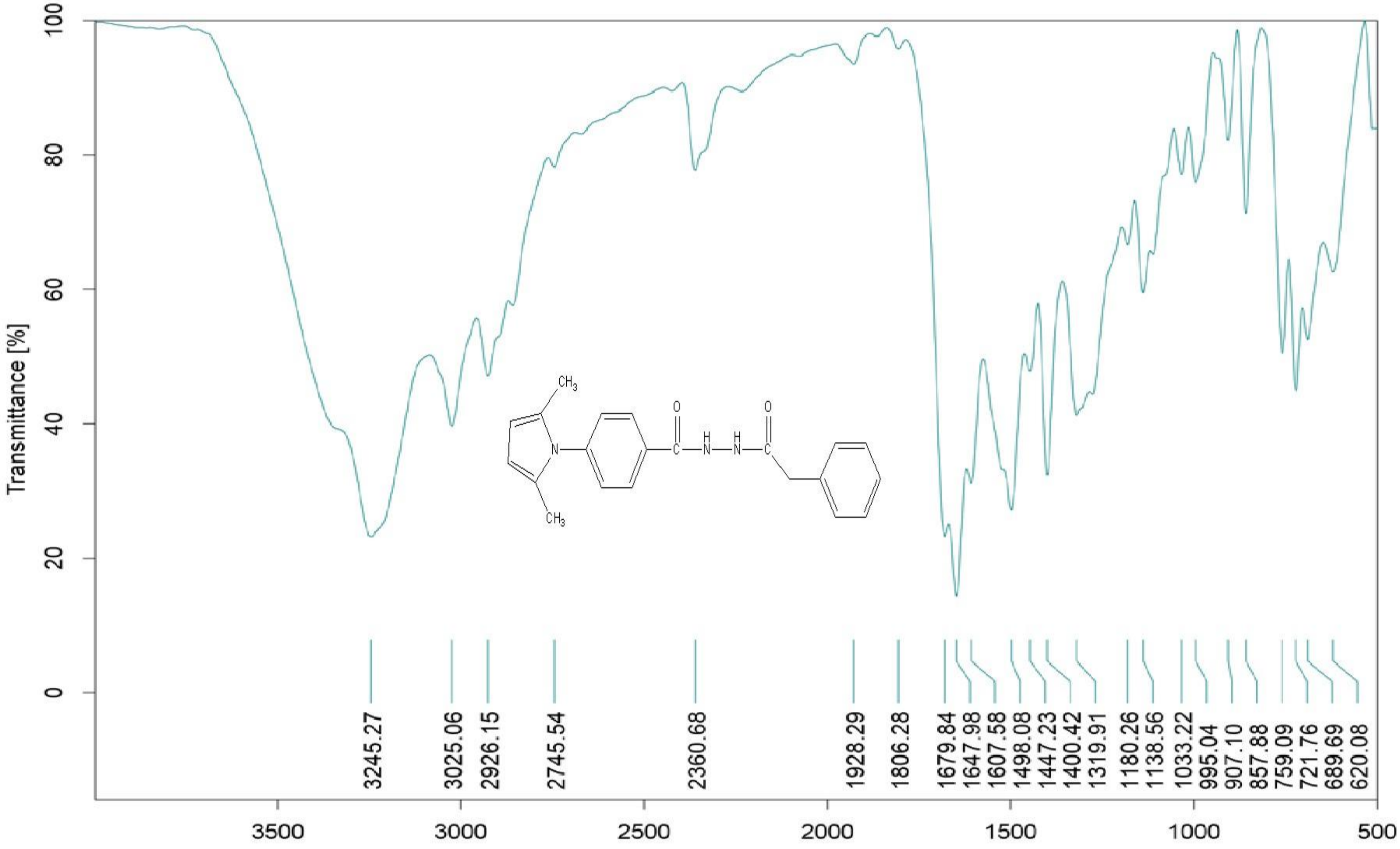

SPECTRUM NO :02 (^{1}H NMR spectrum of Compound 5a)

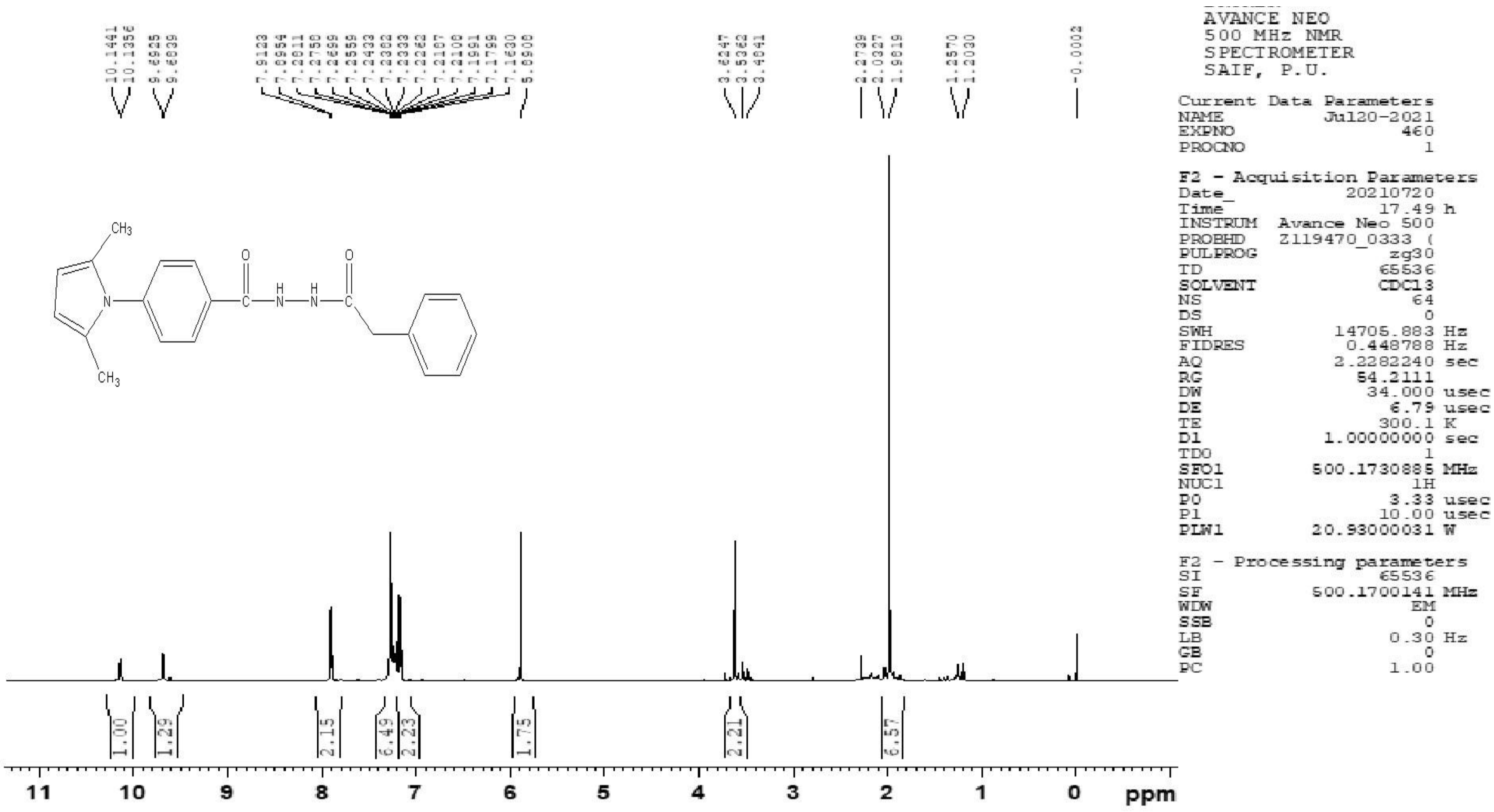

SPECTRUM NO:03 (^{13}C NMR spectrum of Compound 5a)

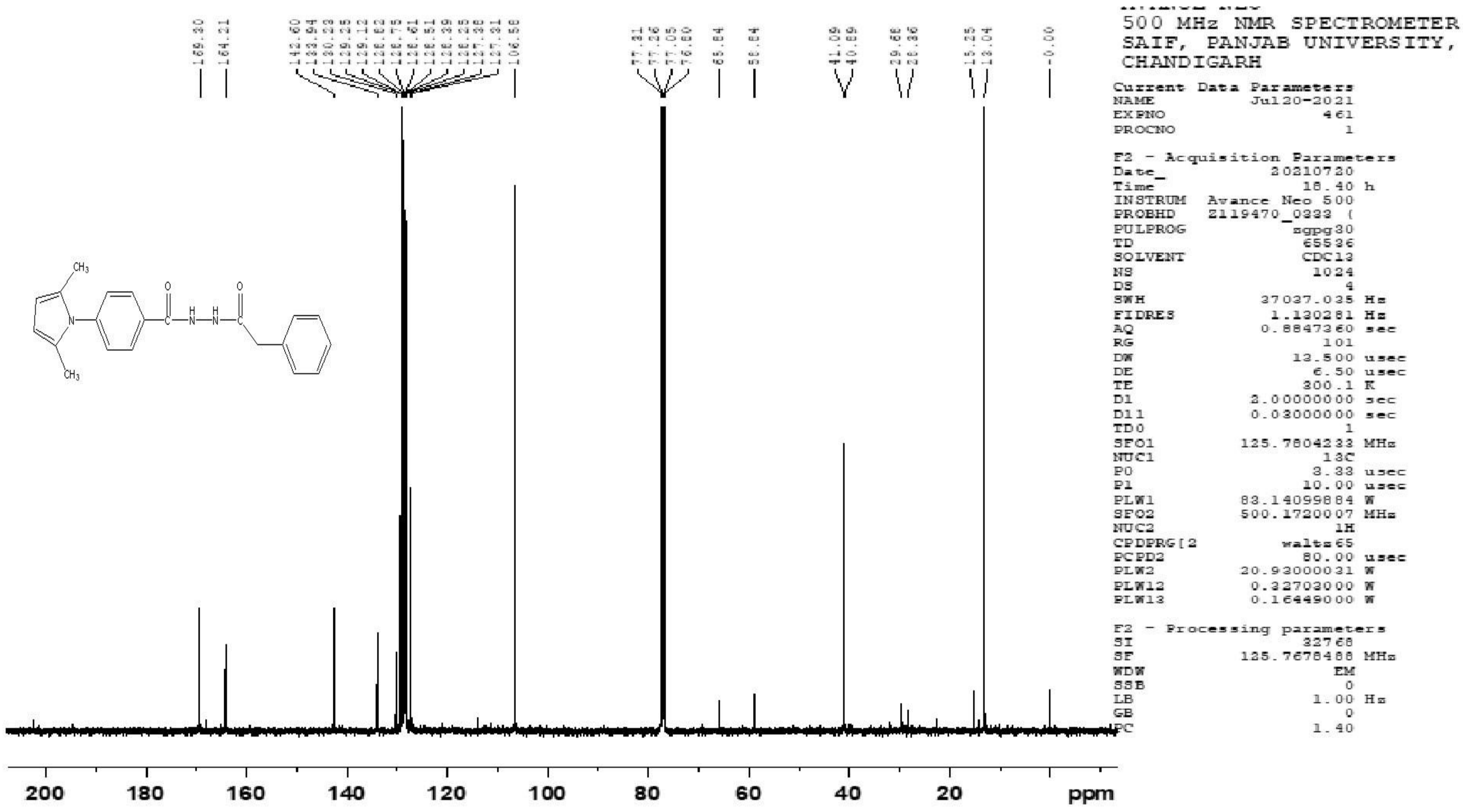

SPECTRUM NO:04 (Mass spectrum of Compound 5a)

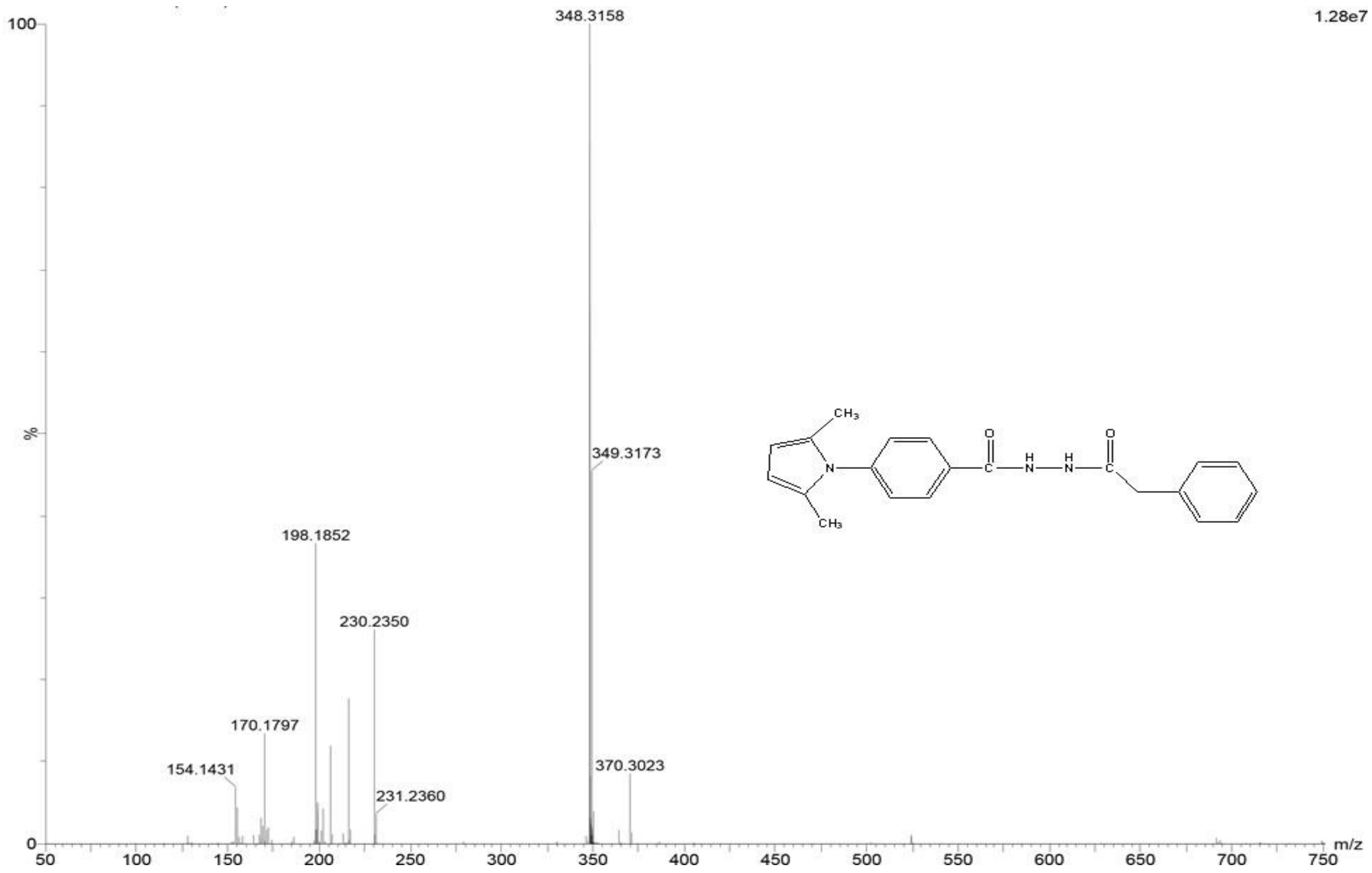

SPECTRUM NO:05 (IR spectrum of Compound 5b)

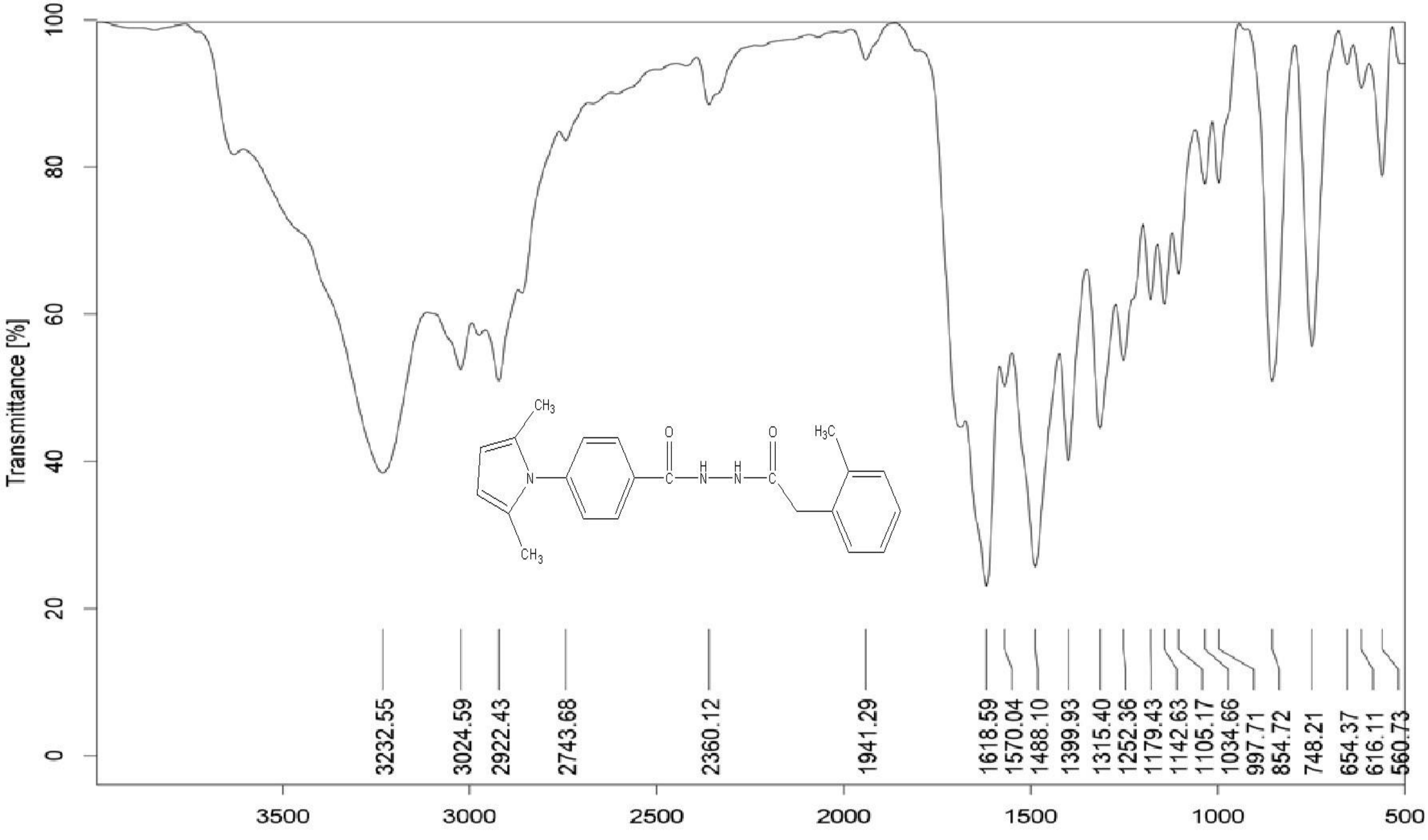

SPECTRUM NO:06 (^{1}H NMR spectrum of Compound 5b)

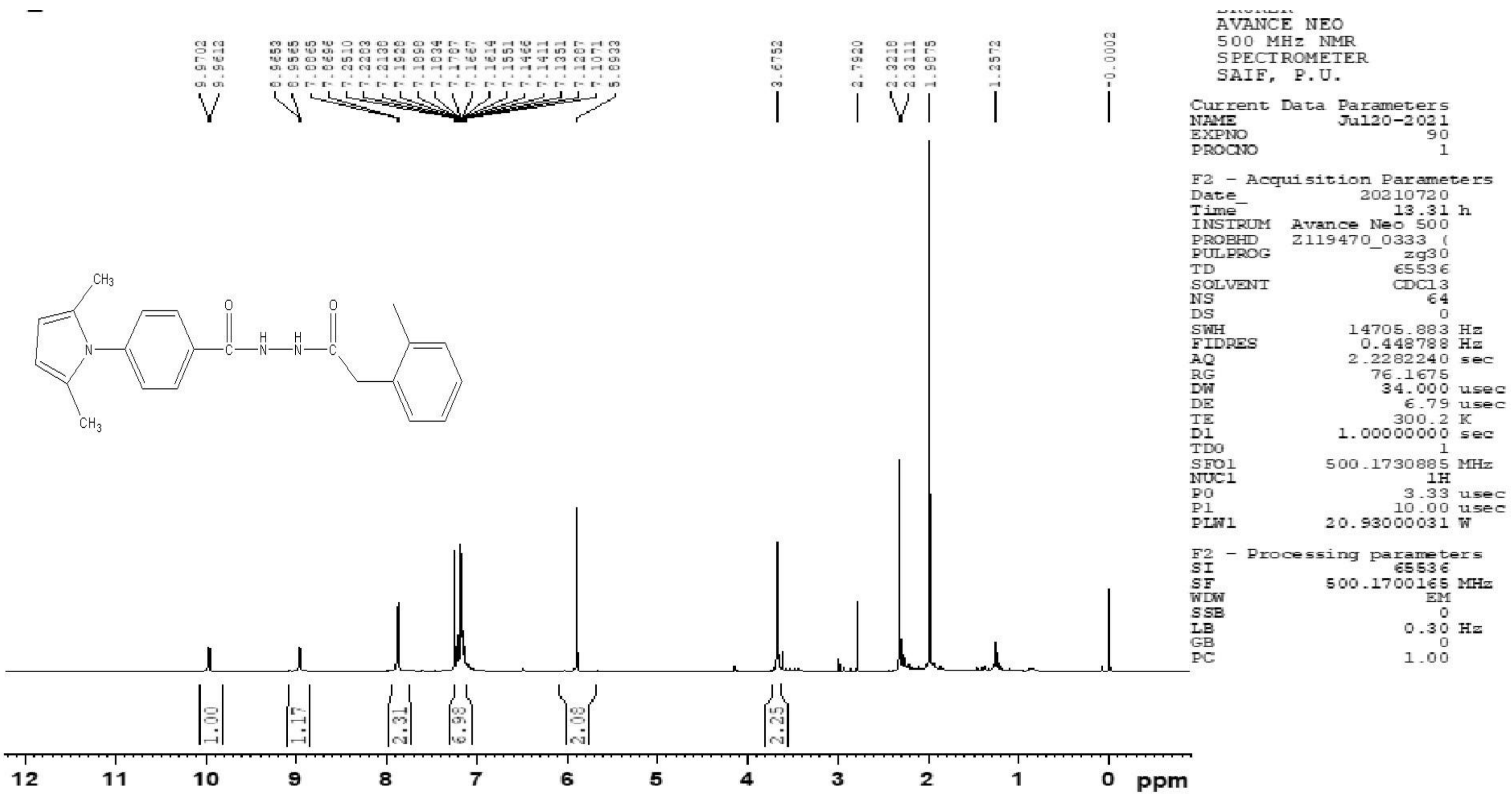

SPECTRUM NO:07 (^{13}C NMR spectrum of Compound 5b)

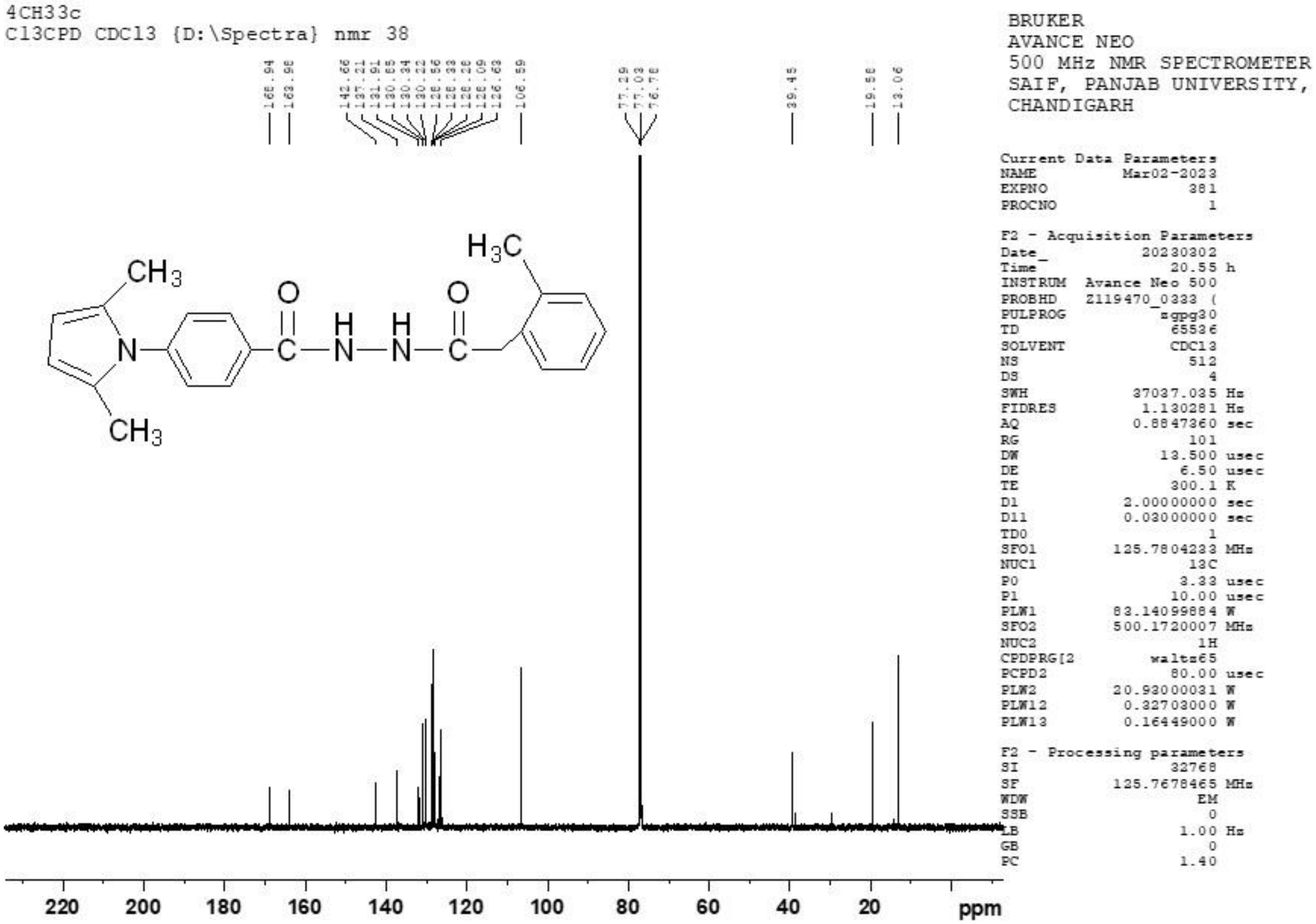

SPECTRUM NO:08 (Mass spectrum of Compound 5b)

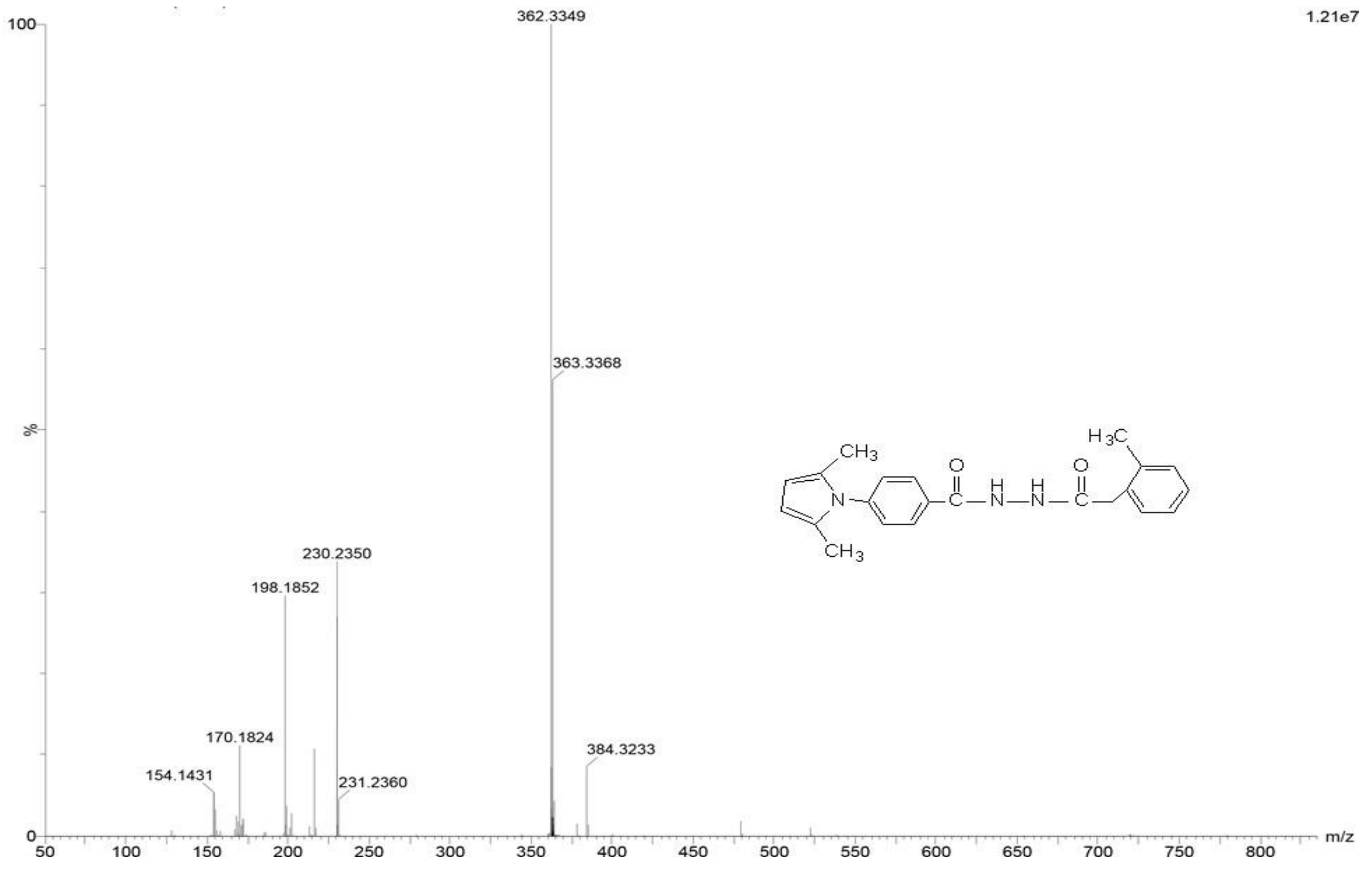

SPECTRUM NO :09 (IR spectrum of Compound 5c)

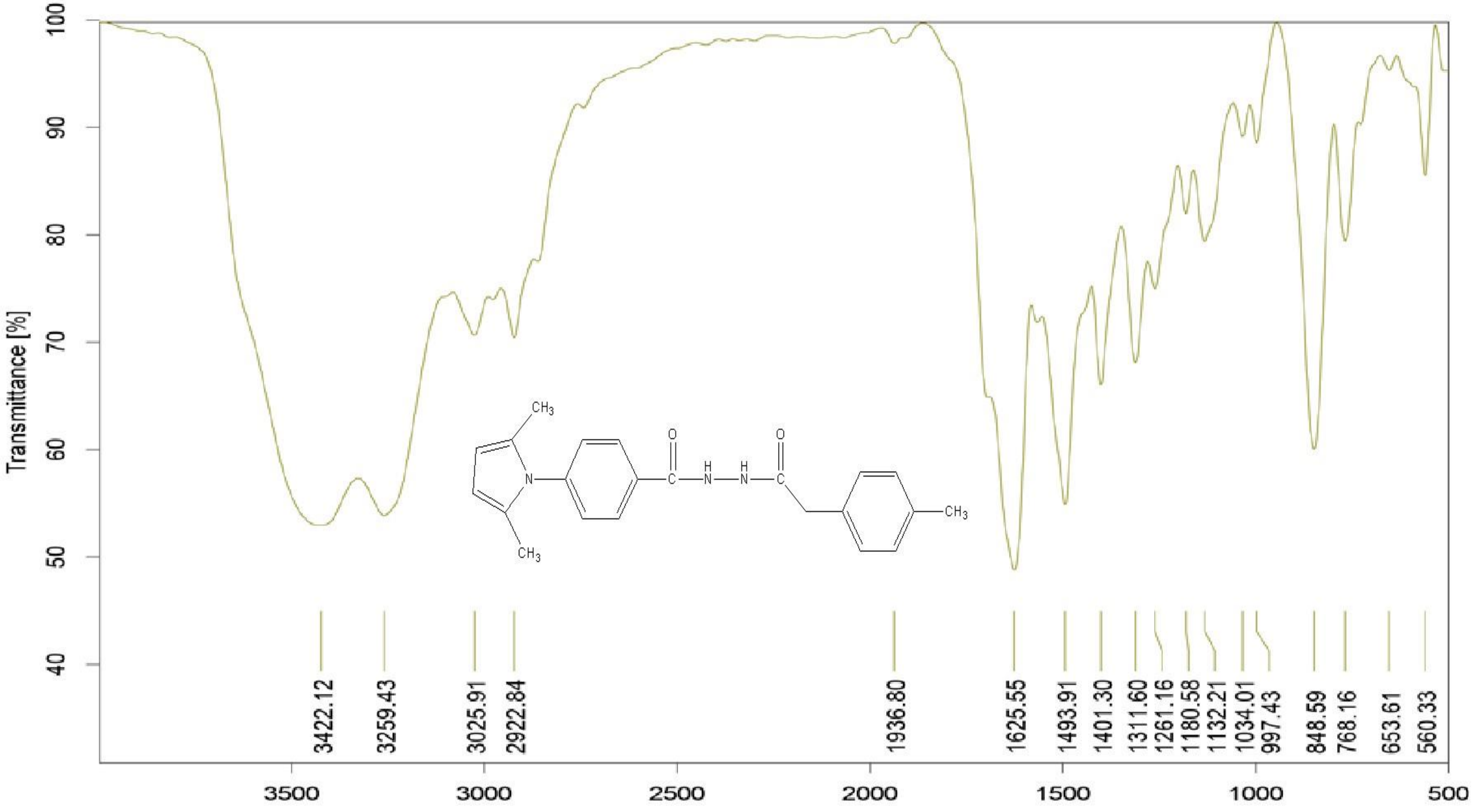

SPECTRUM NO:10 (^{1}H NMR spectrum of Compound 5c)

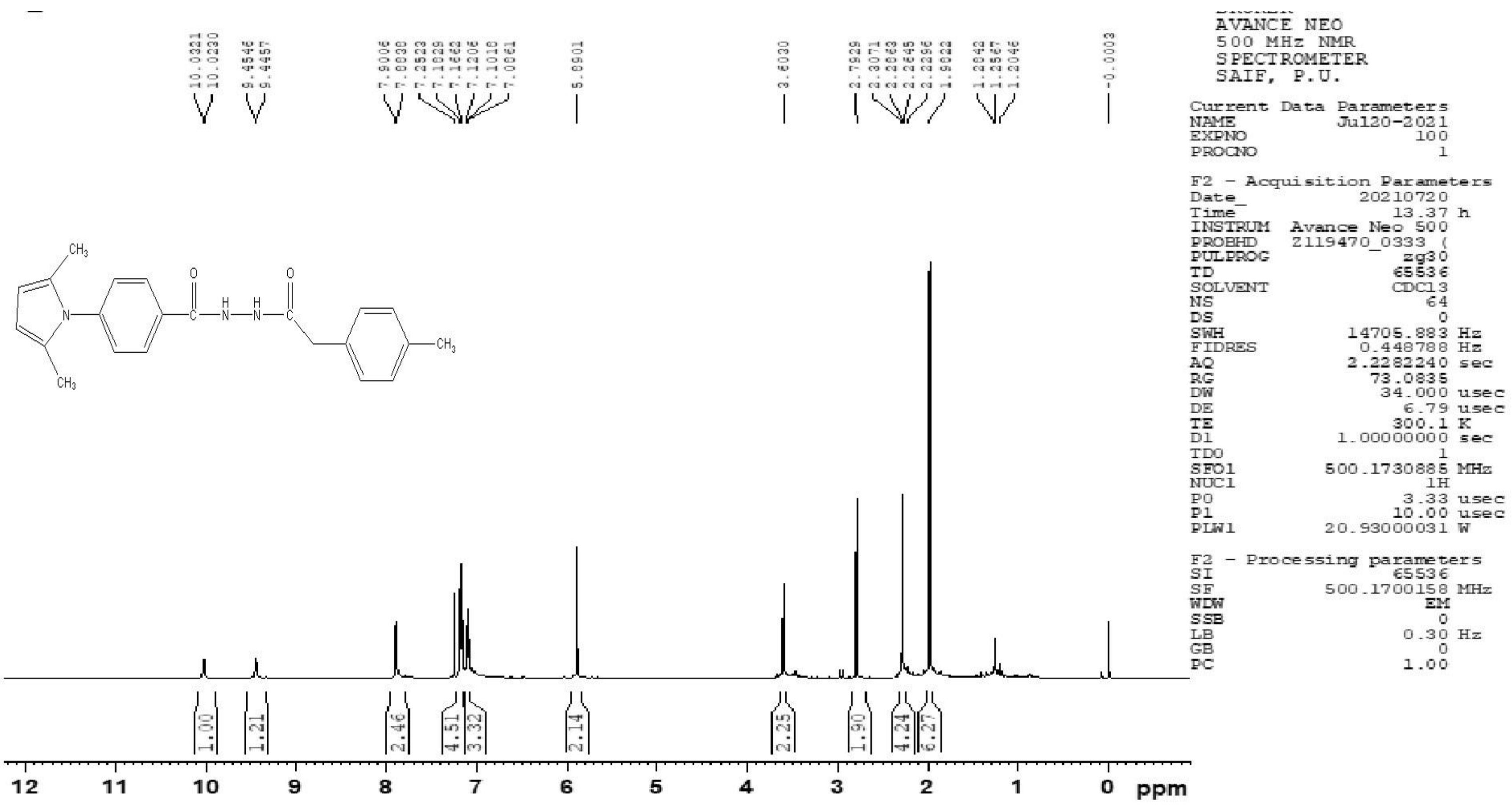

SPECTRUM NO:11 (^{13}C NMR spectrum of Compound 5c)

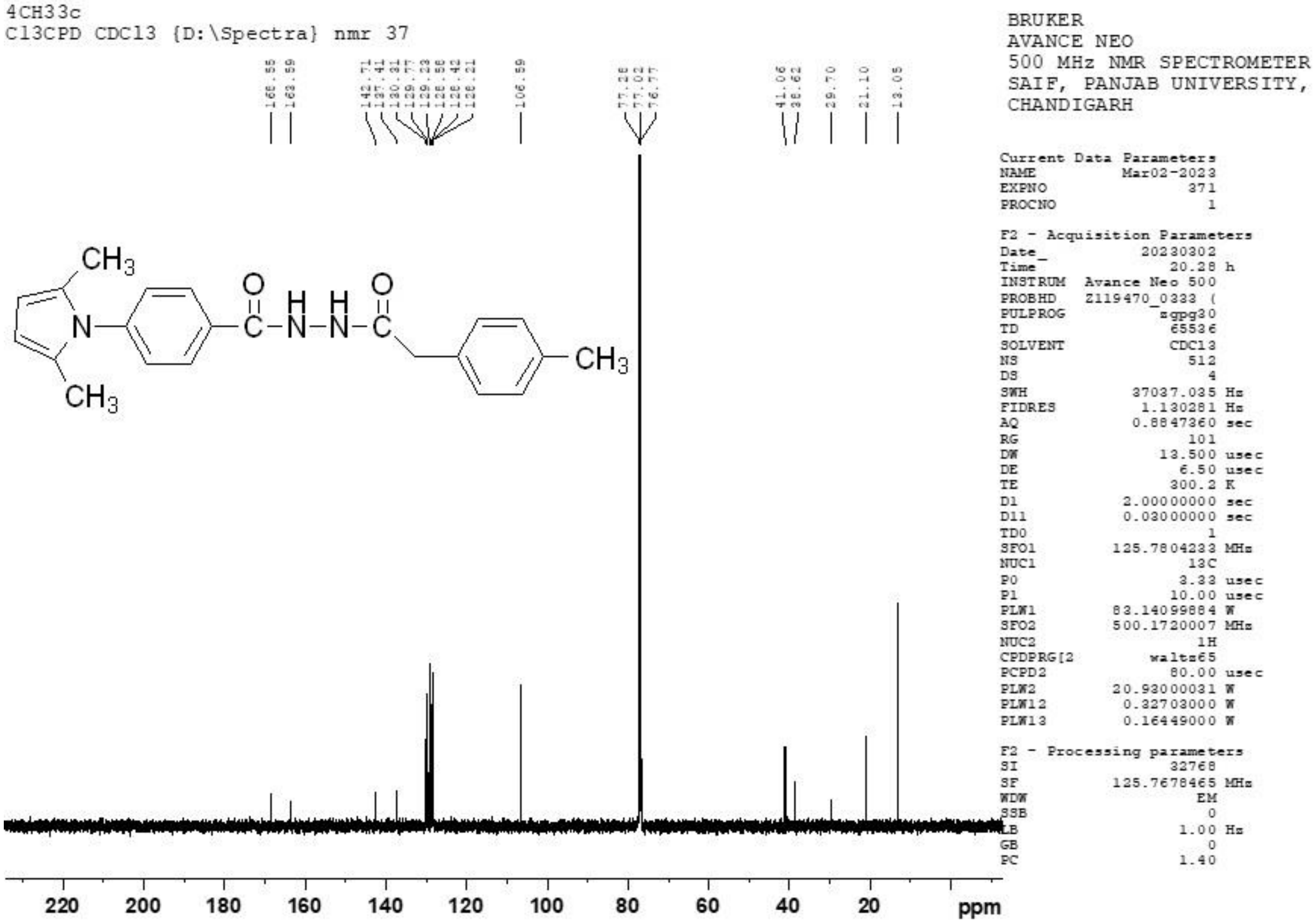

SPECTRUM NO:12 (Mass spectrum of Compound 5c)

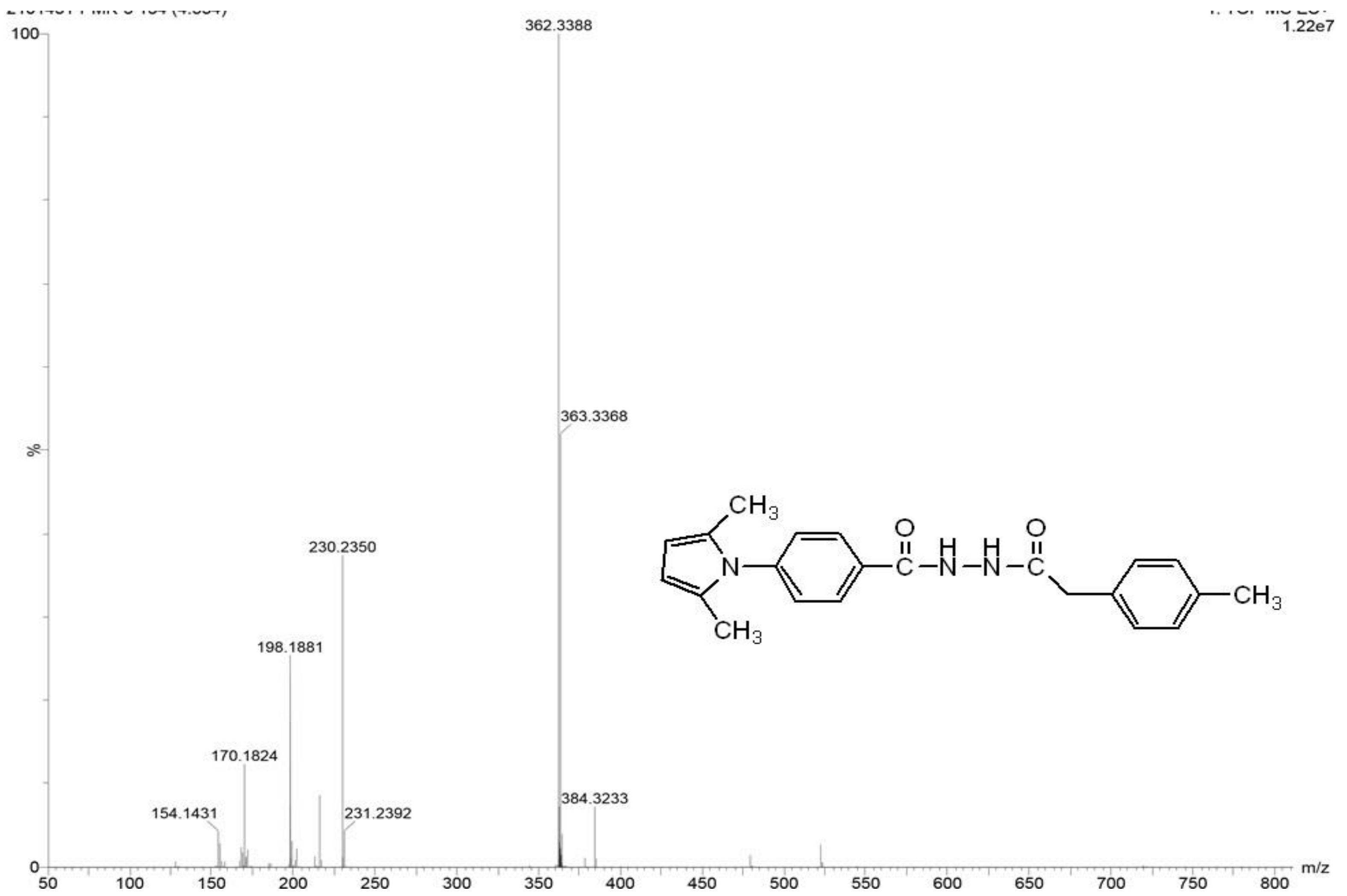

SPECTRUM NO:13 (IR spectrum of Compound 5d)

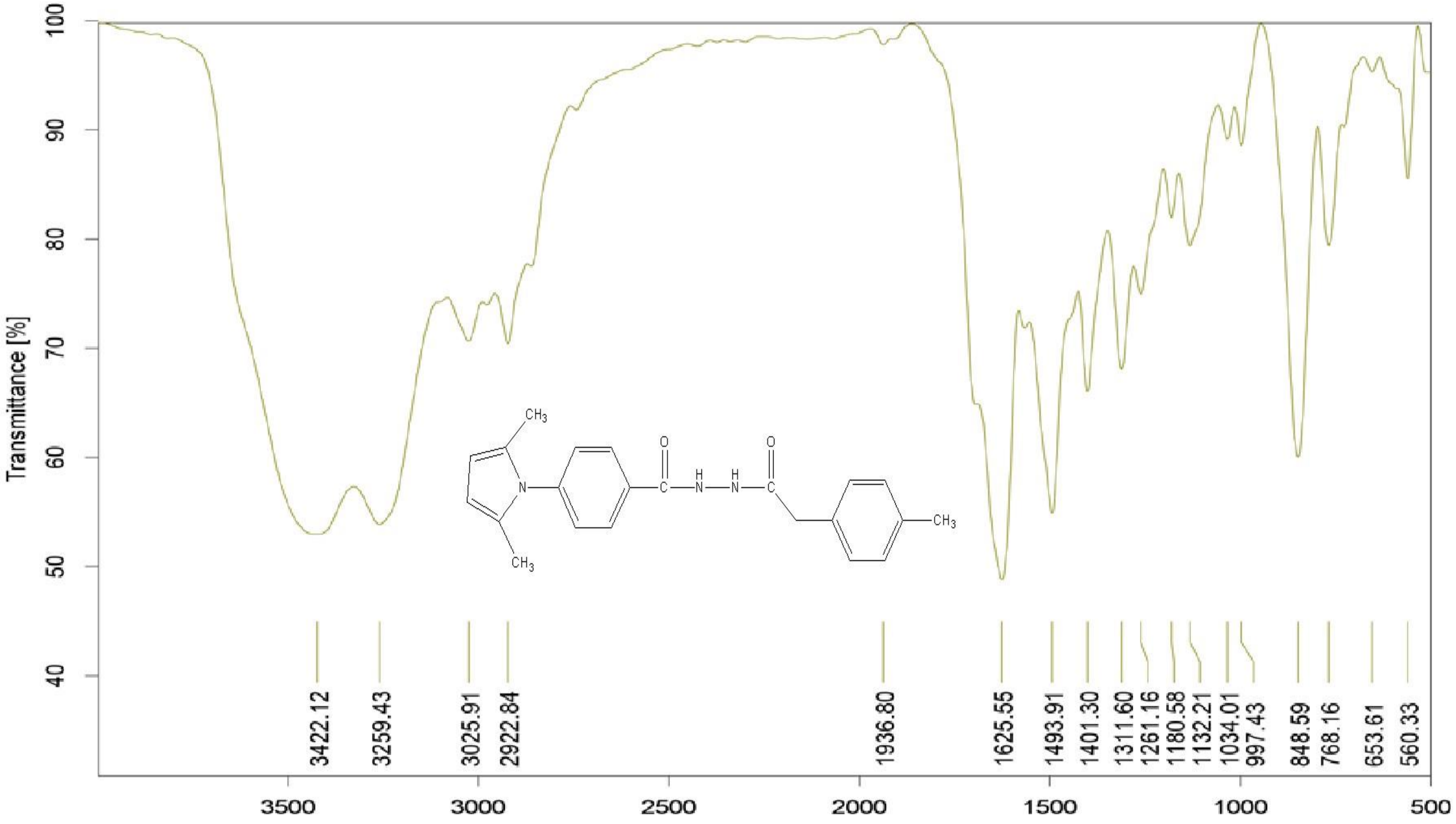

SPECTRUM NO:14 (^{1}H NMR spectrum of Compound 5d)

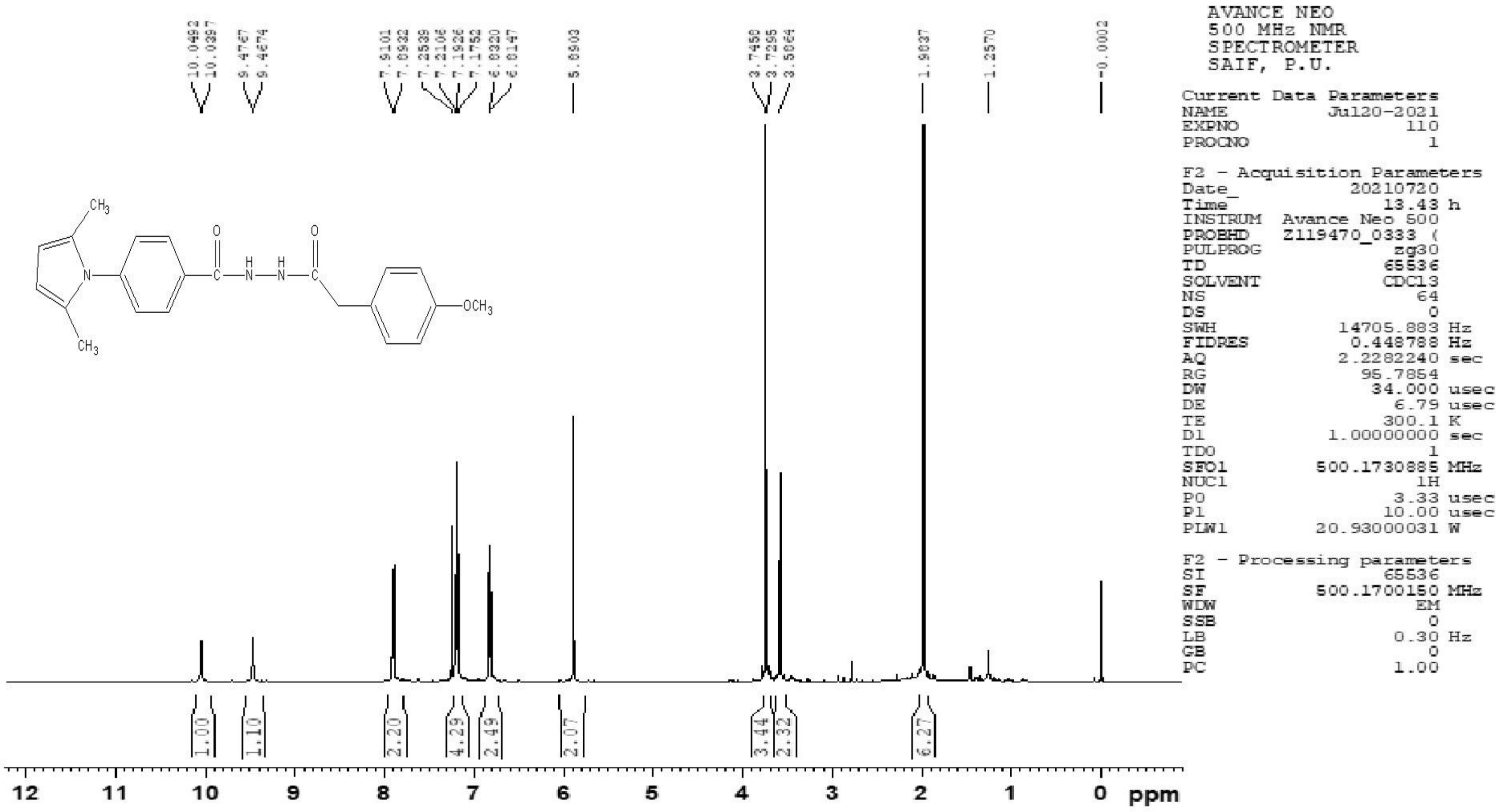

SPECTRUM NO:15 (^{13}C NMR spectrum of Compound 5d)

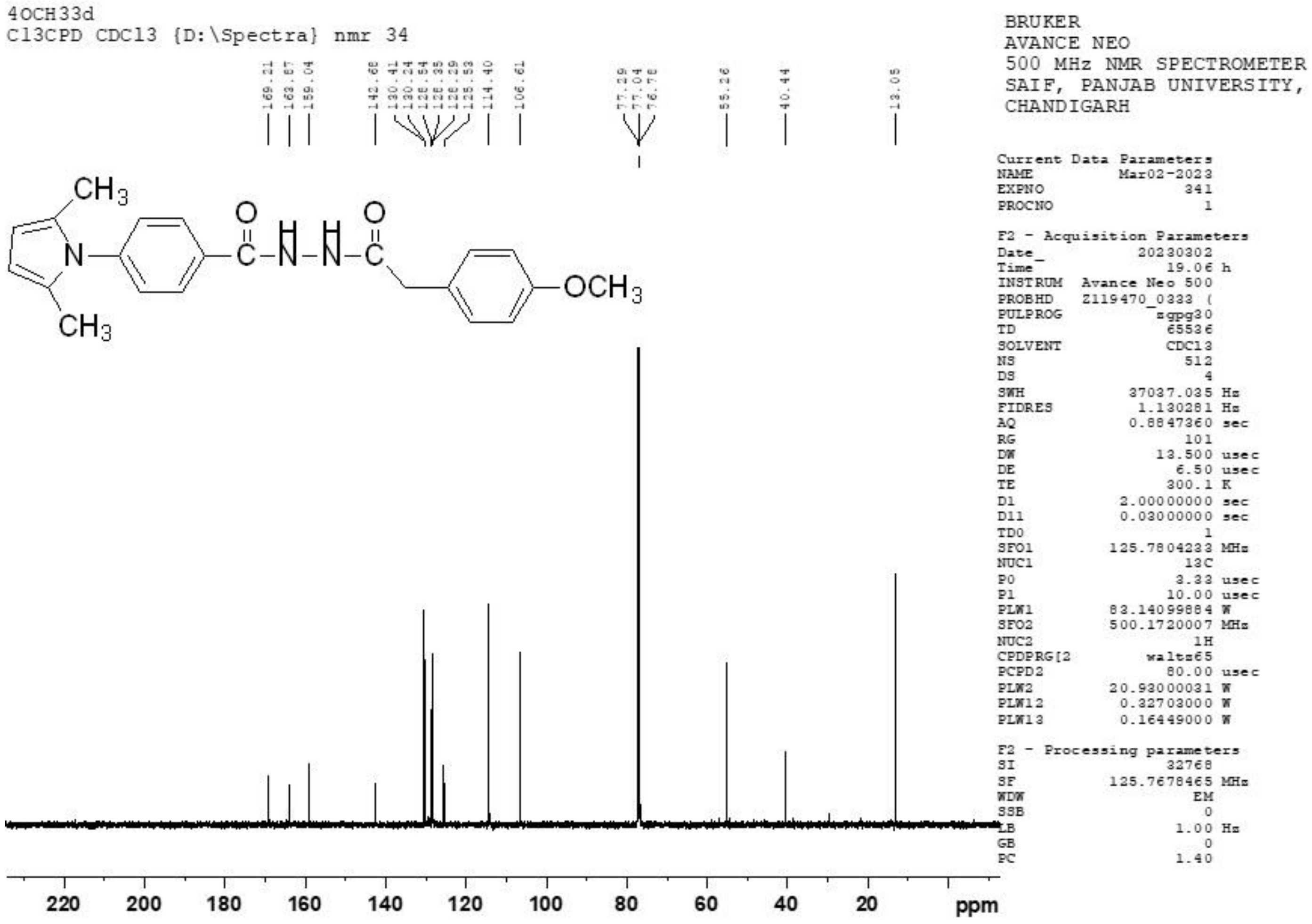

SPECTRUM NO:16 (Mass spectrum of Compound 5d)

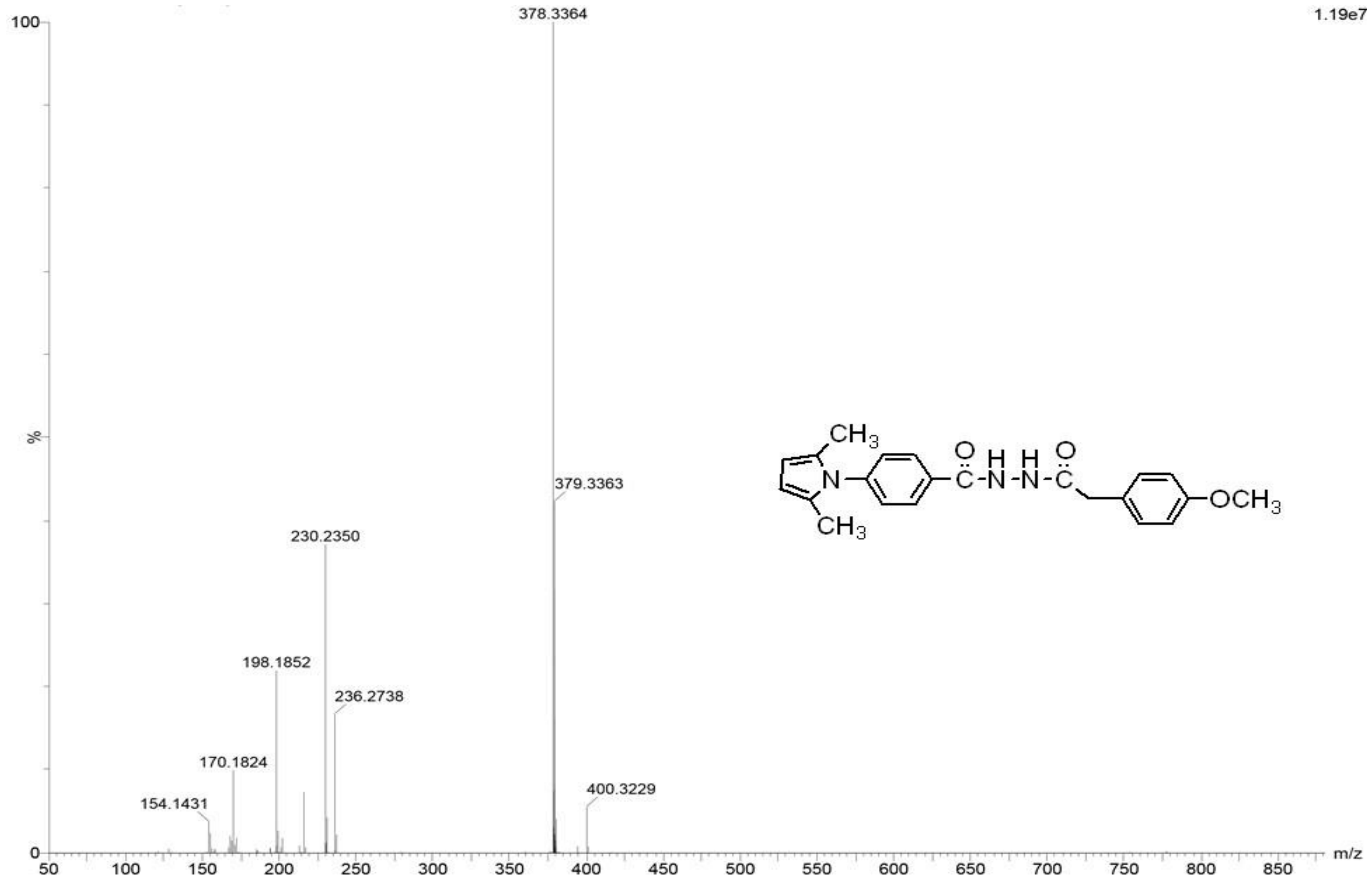

SPECTRUM NO:14

SPECTRUM NO :17 (IR spectrum of Compound 5e)

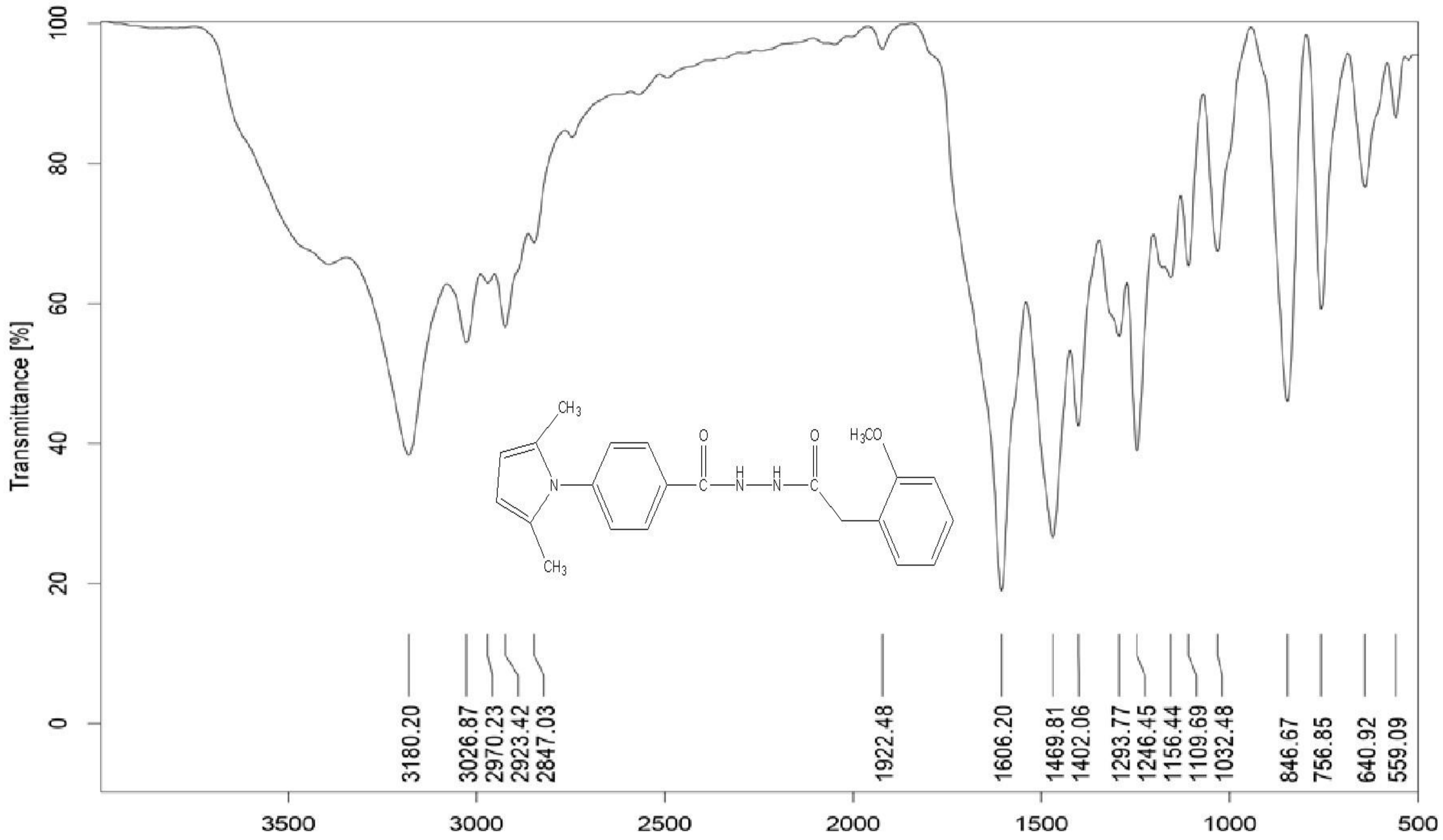

SPECTRUM NO:18 (^{1}H NMR spectrum of Compound 5e)

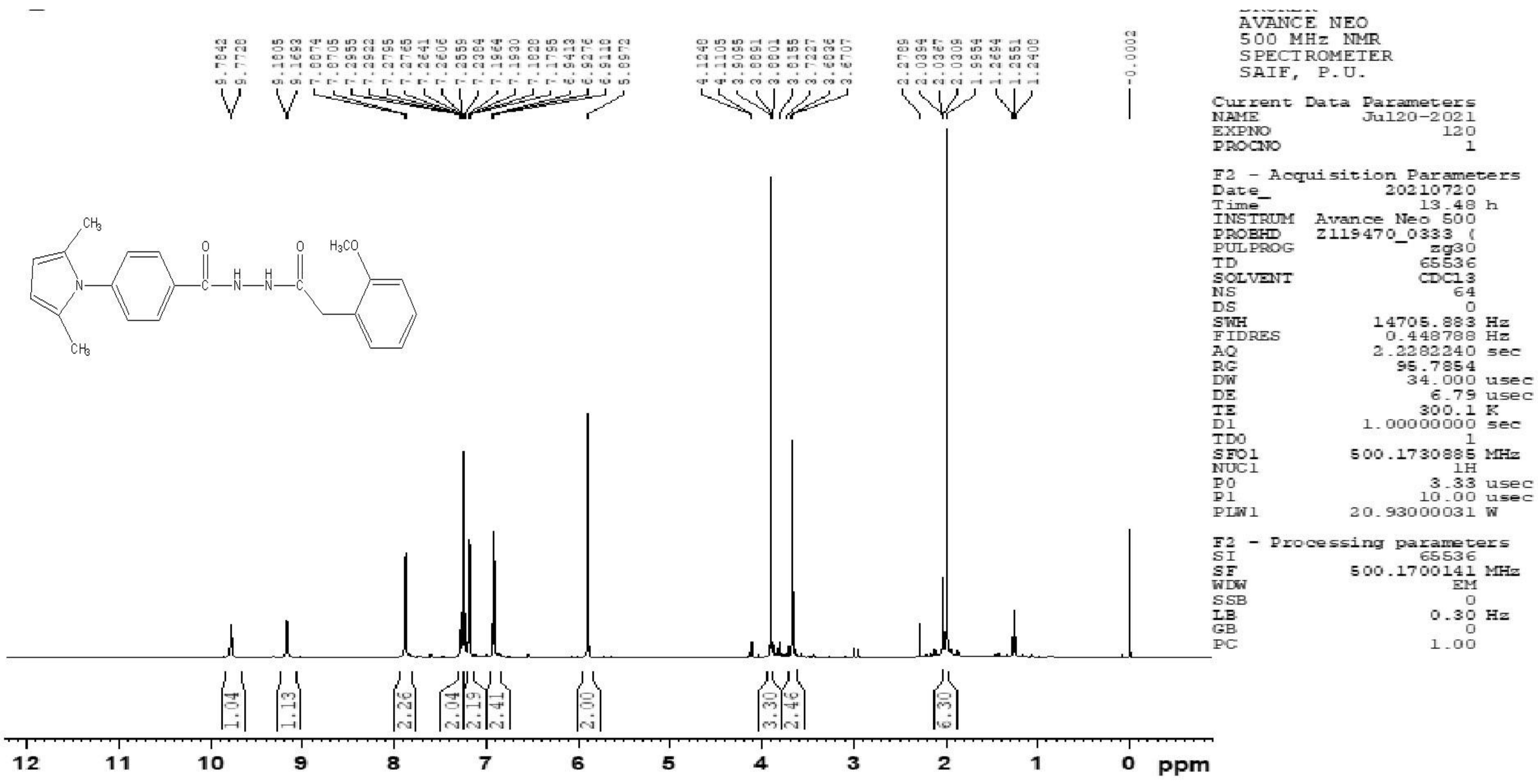

SPECTRUM NO:19 (^{13}C NMR spectrum of Compound 5e)

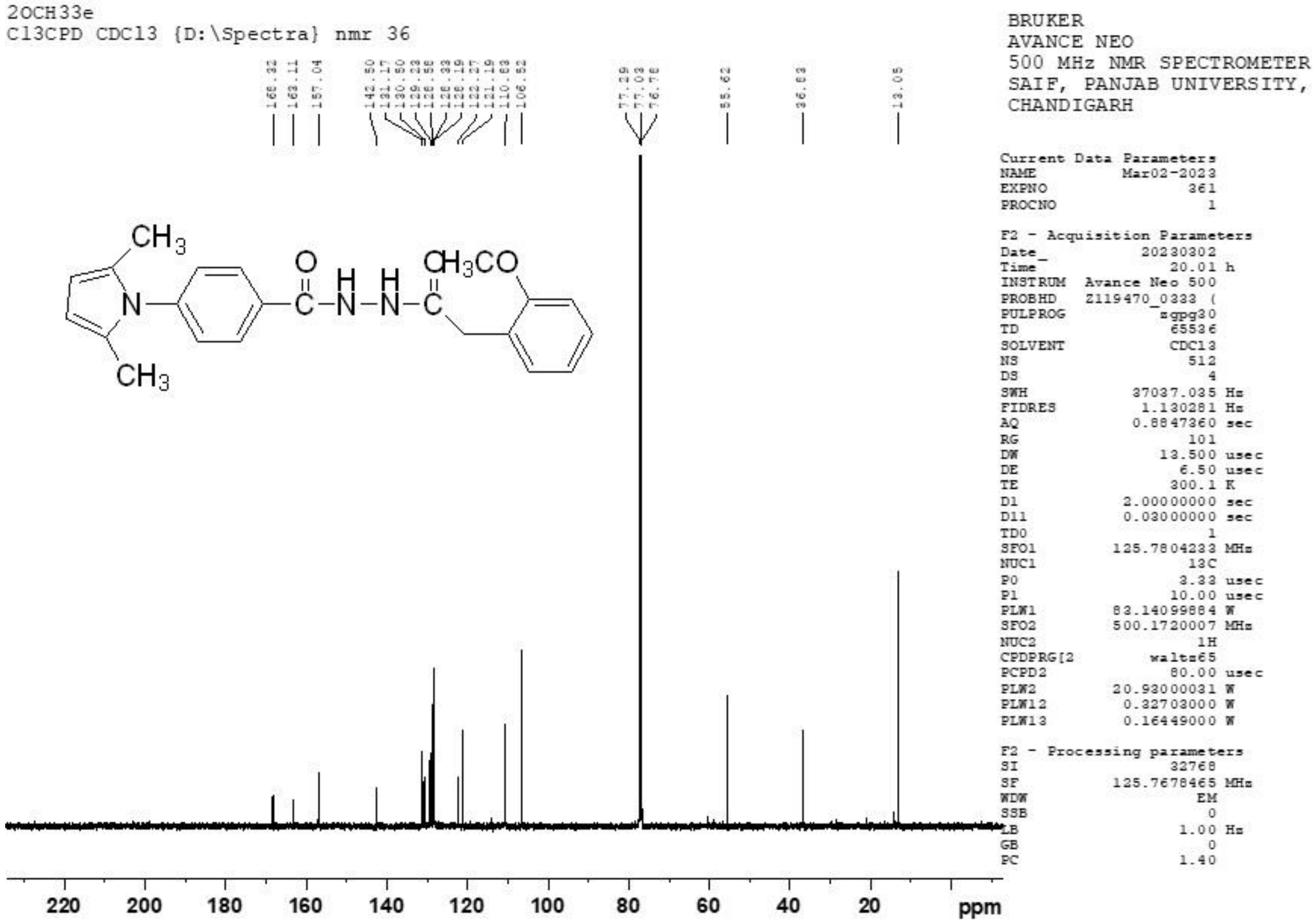

SPECTRUM NO:20 (Mass spectrum of Compound 5e)

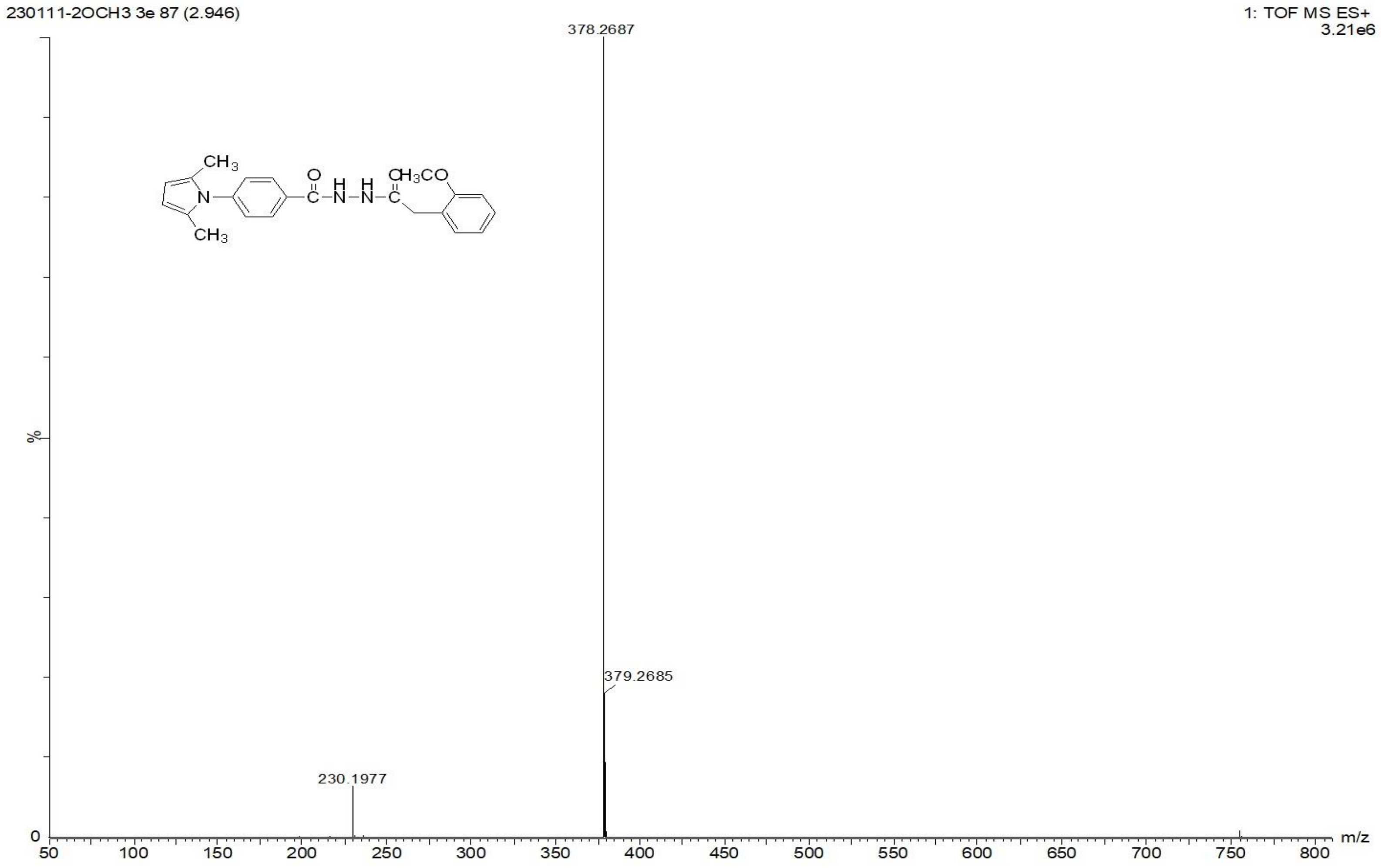

SPECTRUM NO: 21(IR spectrum of Compound 5f)

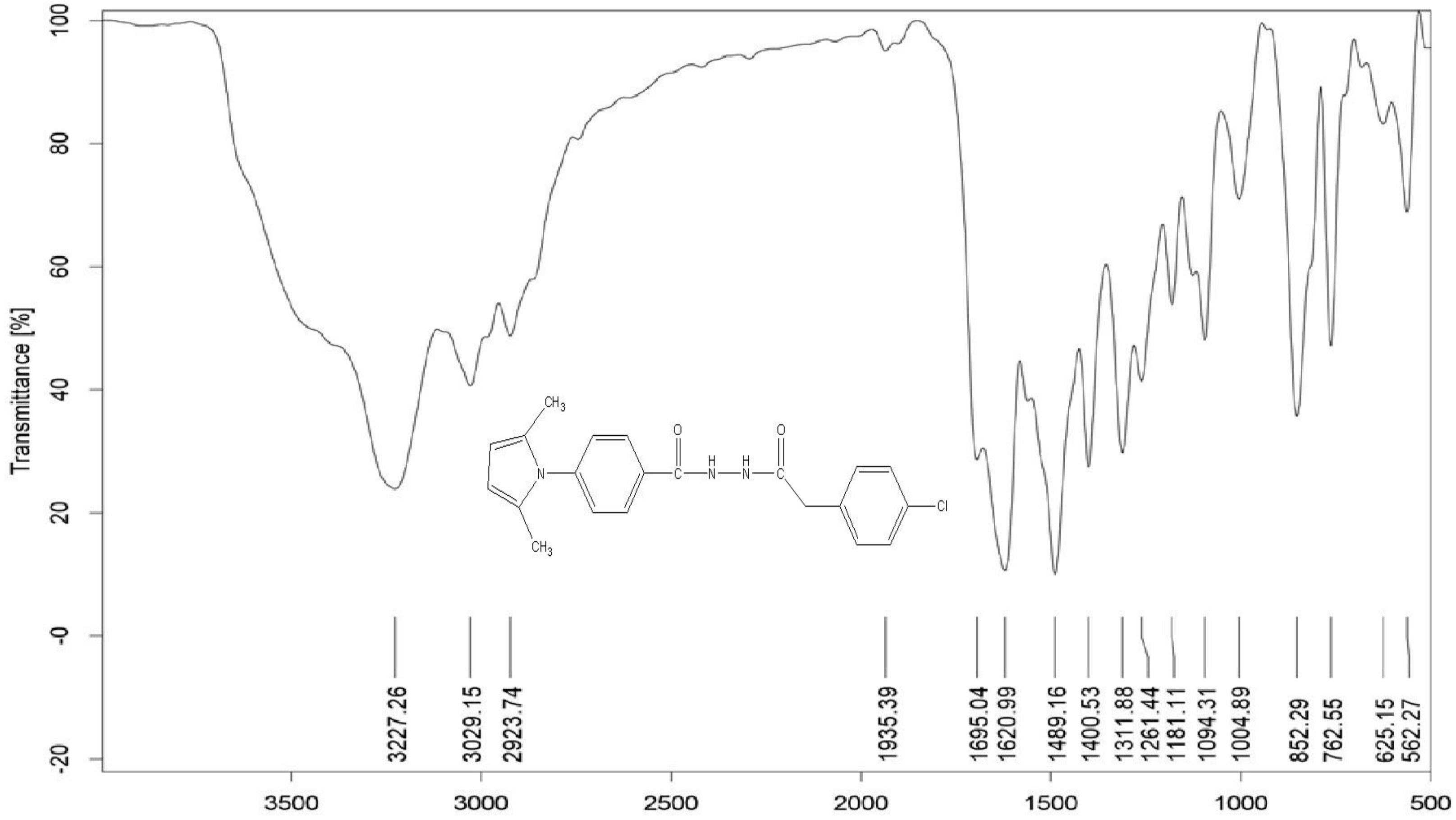

SPECTRUM NO: 22 (^{1}H NMR spectrum of Compound 5f)

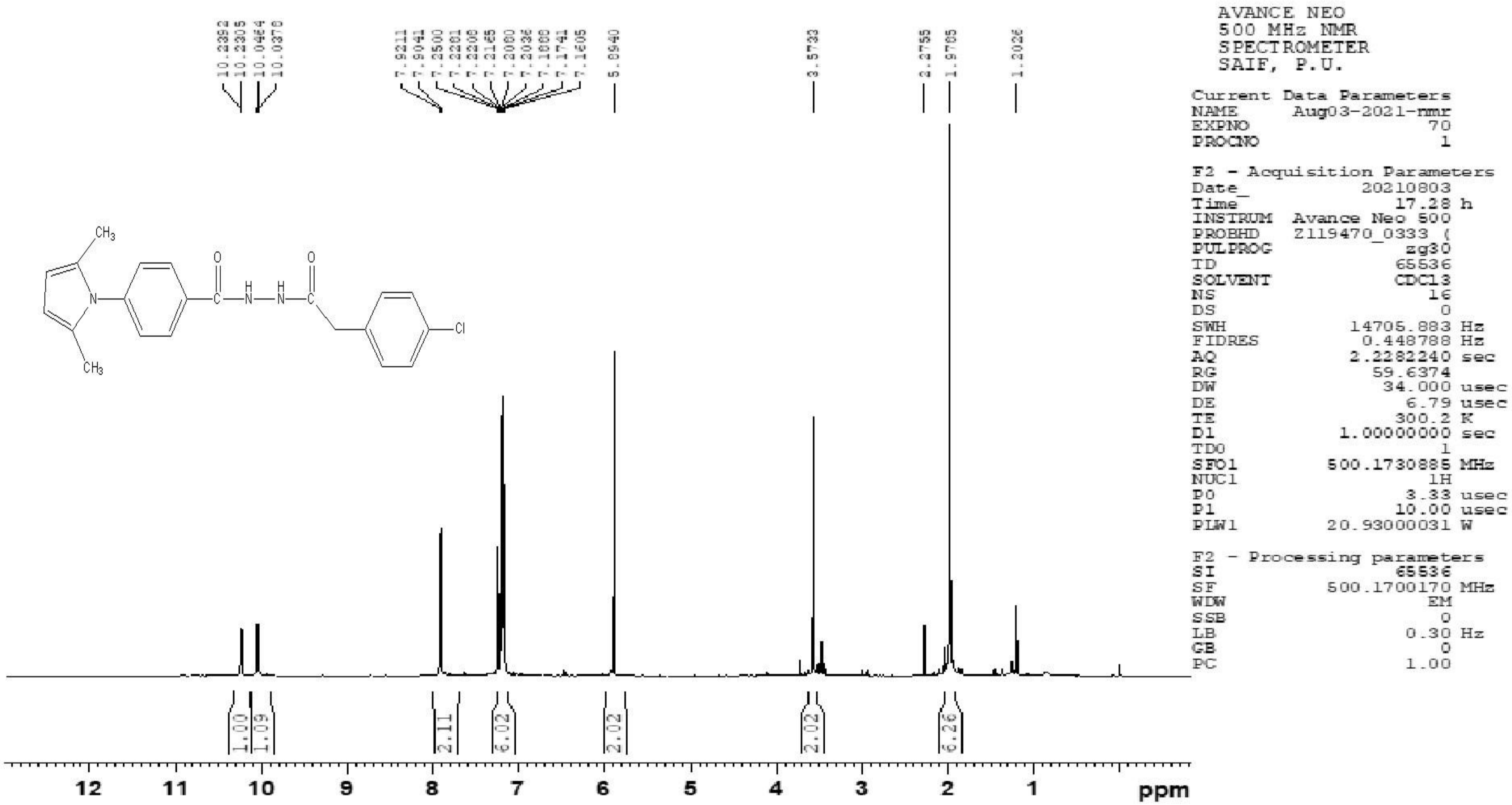

SPECTRUM NO: 23 (^{13}C NMR spectrum of Compound 5f)

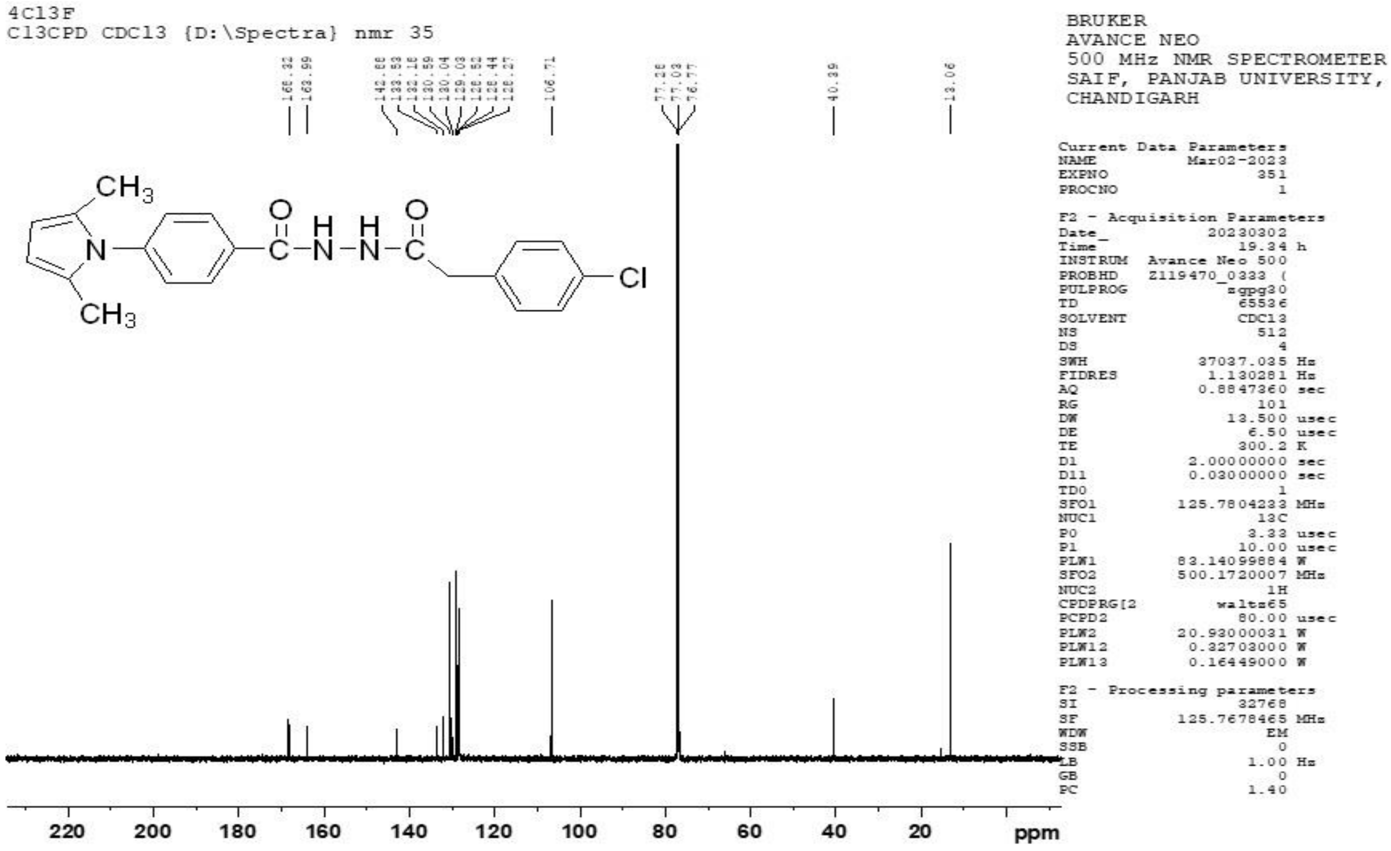

SPECTRUM NO:24 (Mass spectrum of Compound 5f)

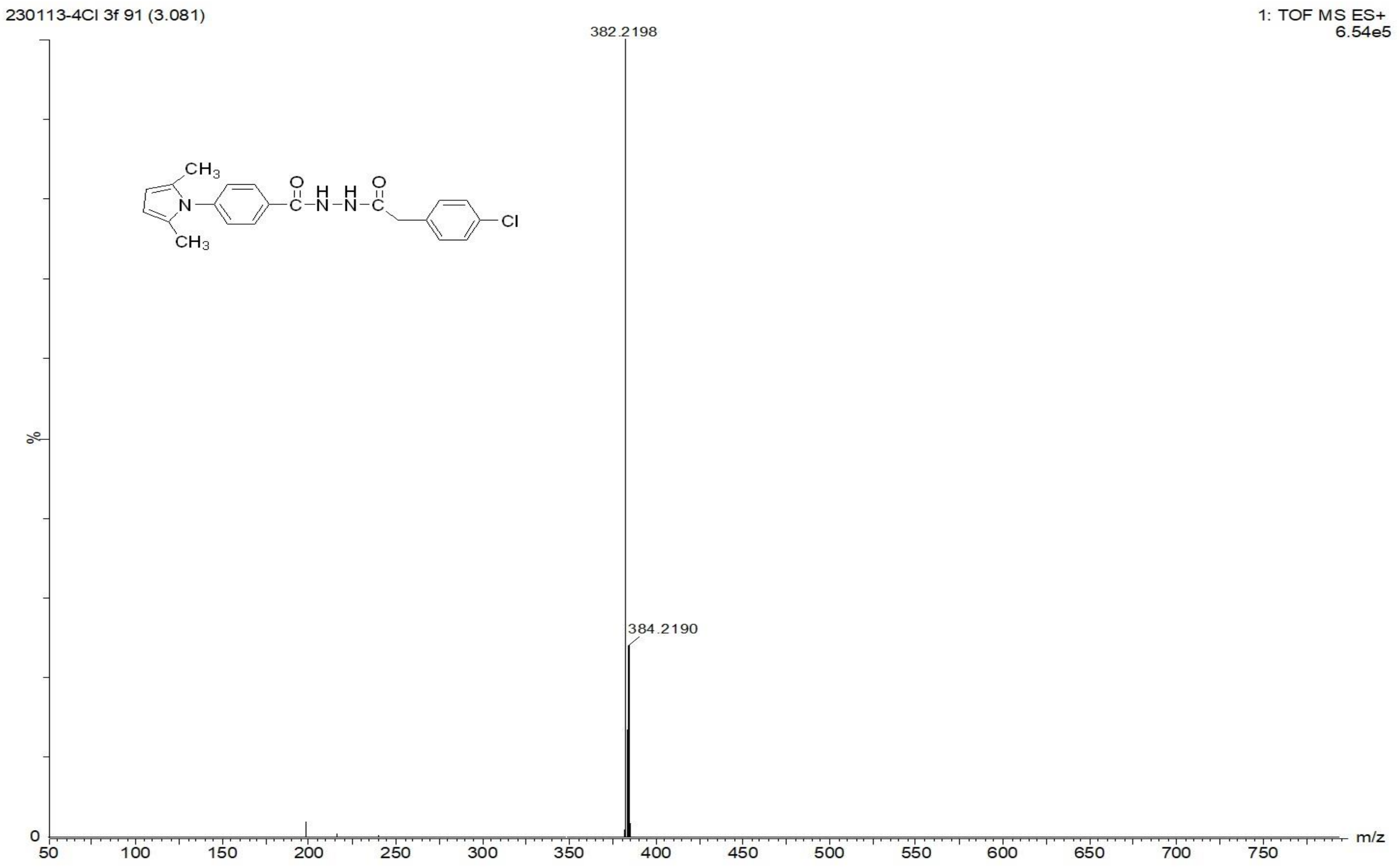

SPECTRUM NO: 25 (IR spectrum of Compound 5g)

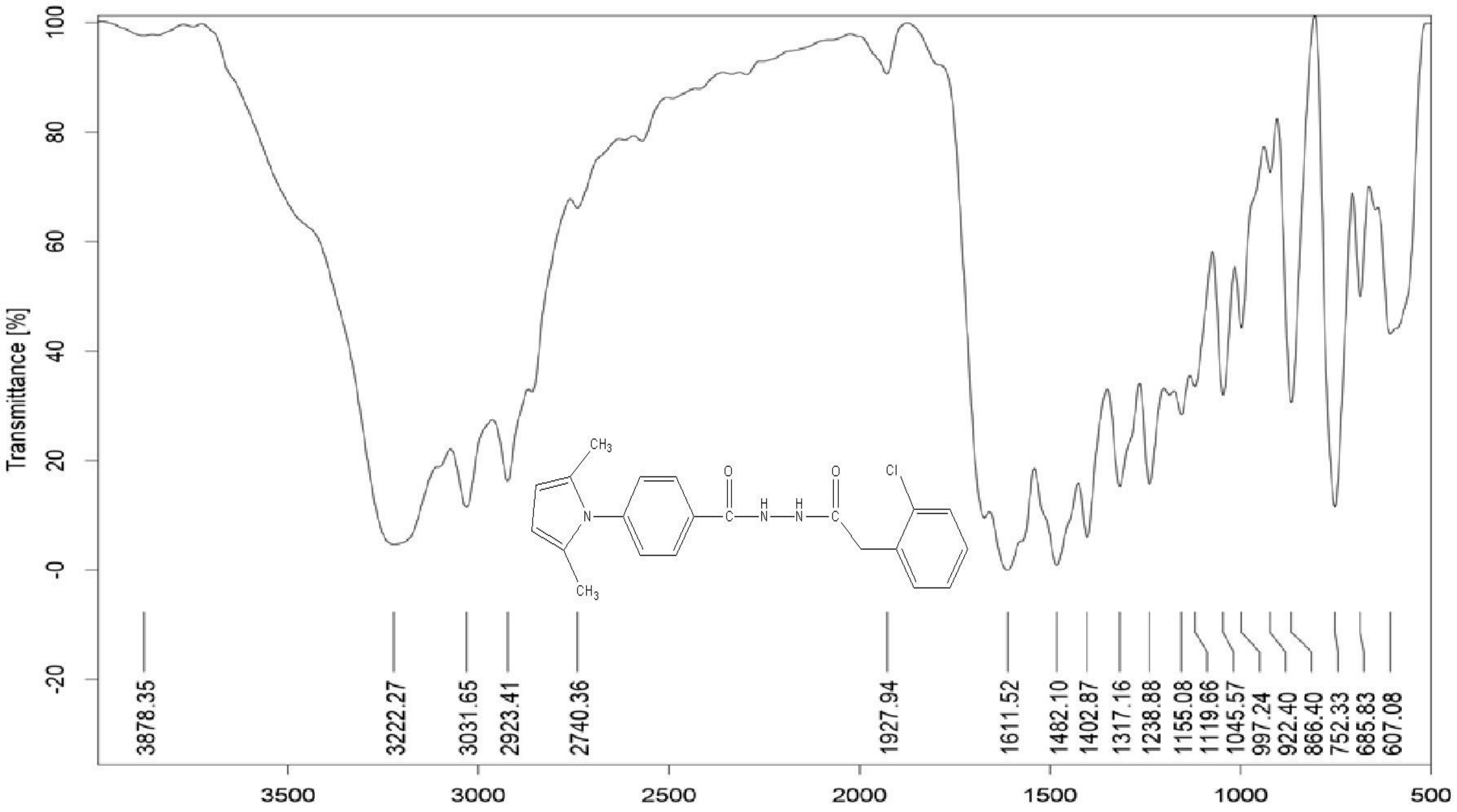

SPECTRUM NO: 26 (^{1}H NMR spectrum of Compound 5g)

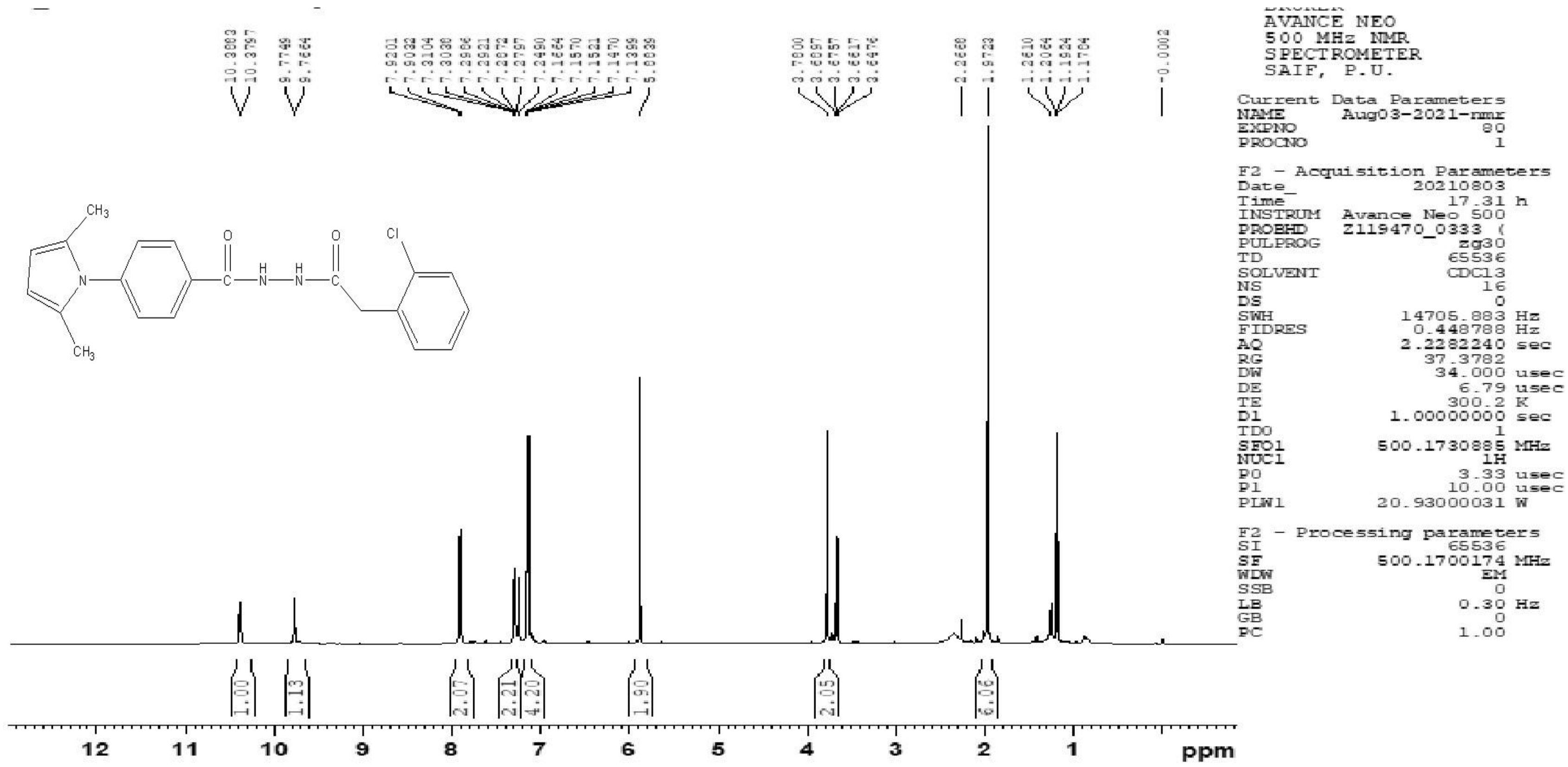

SPECTRUM NO: 27 (^{13}C NMR spectrum of Compound 5g)

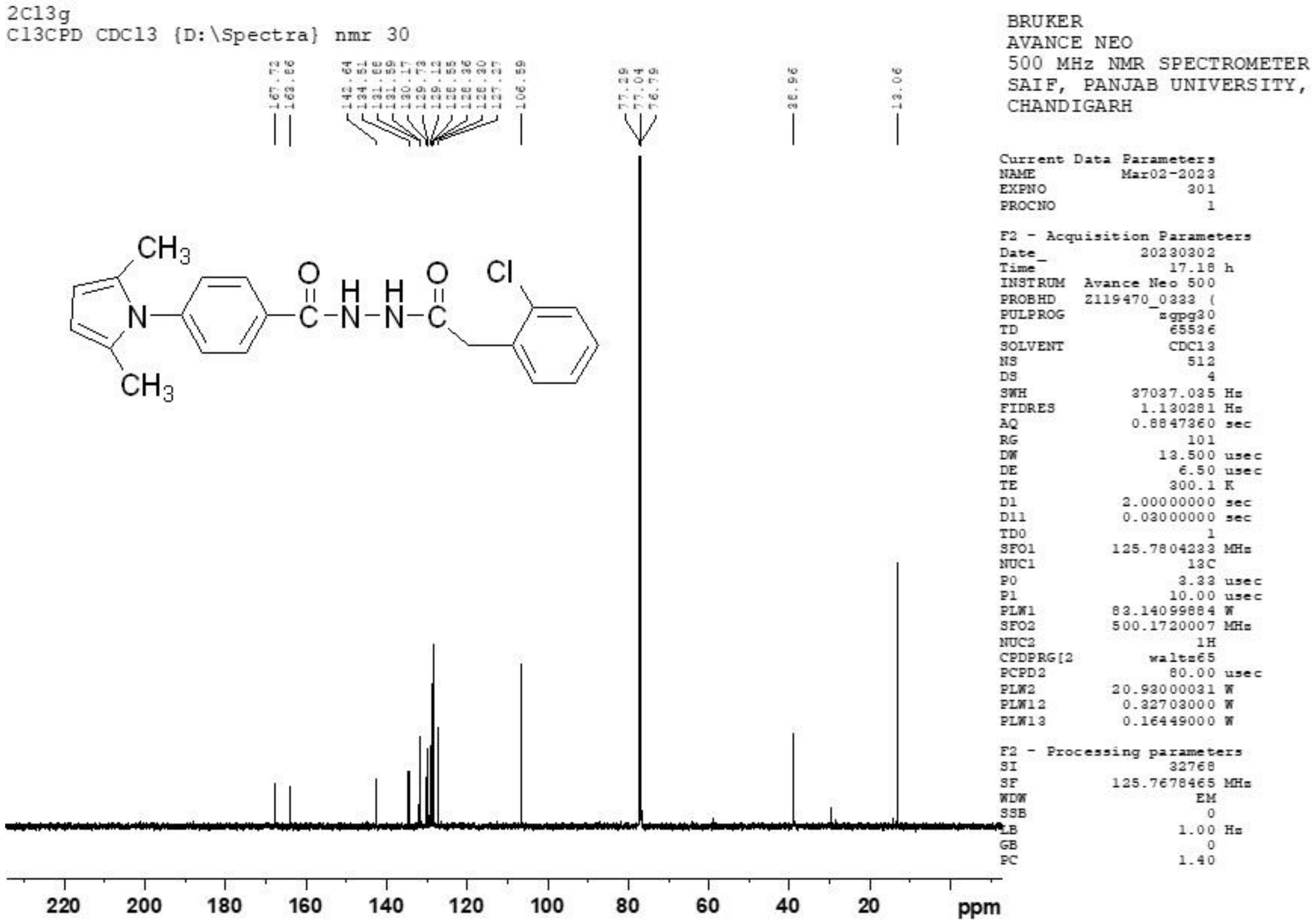

SPECTRUM NO: 28 (^{13}C NMR spectrum of Compound 5g)

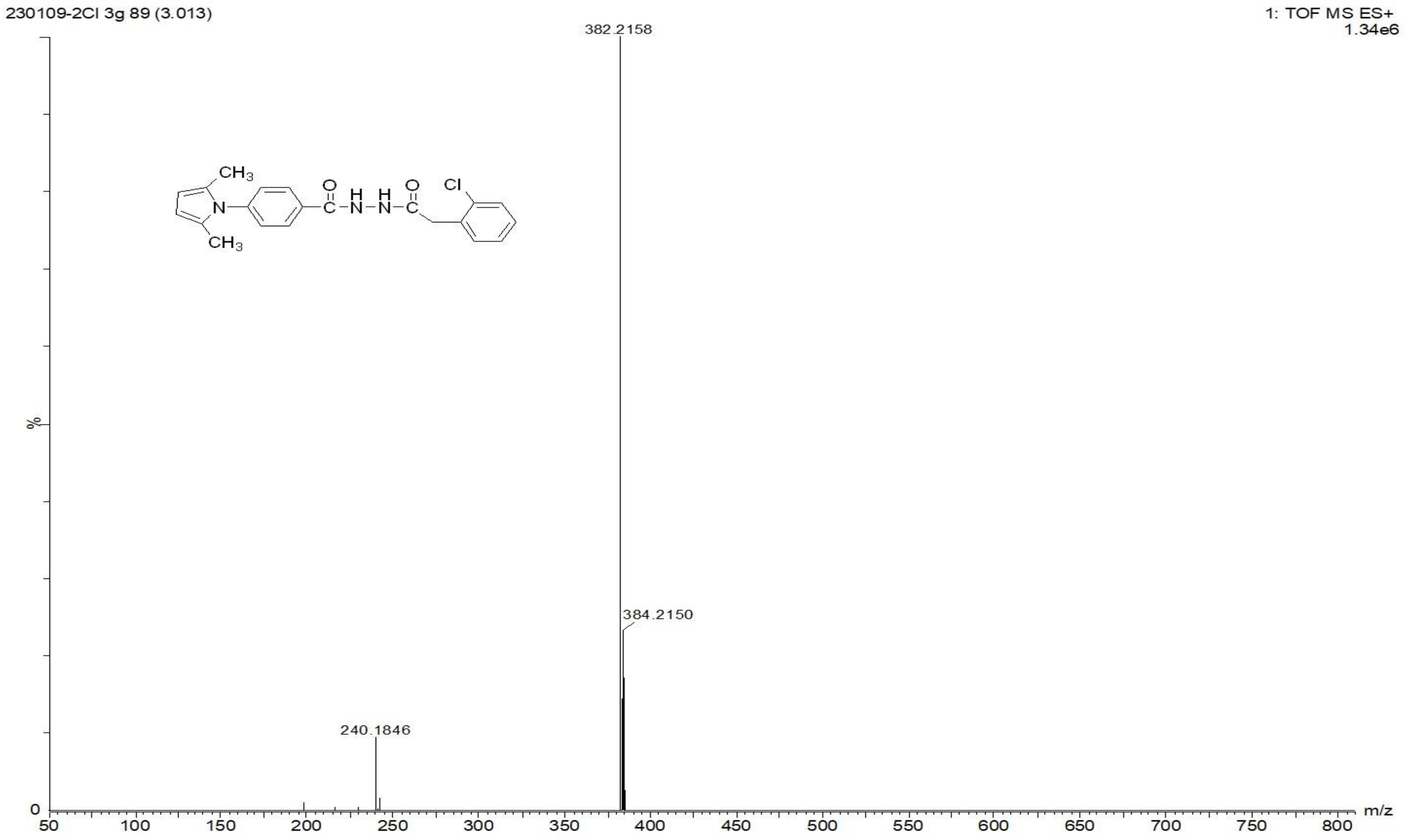

SPECTRUM NO: 29 (IR spectrum of Compound 5h)

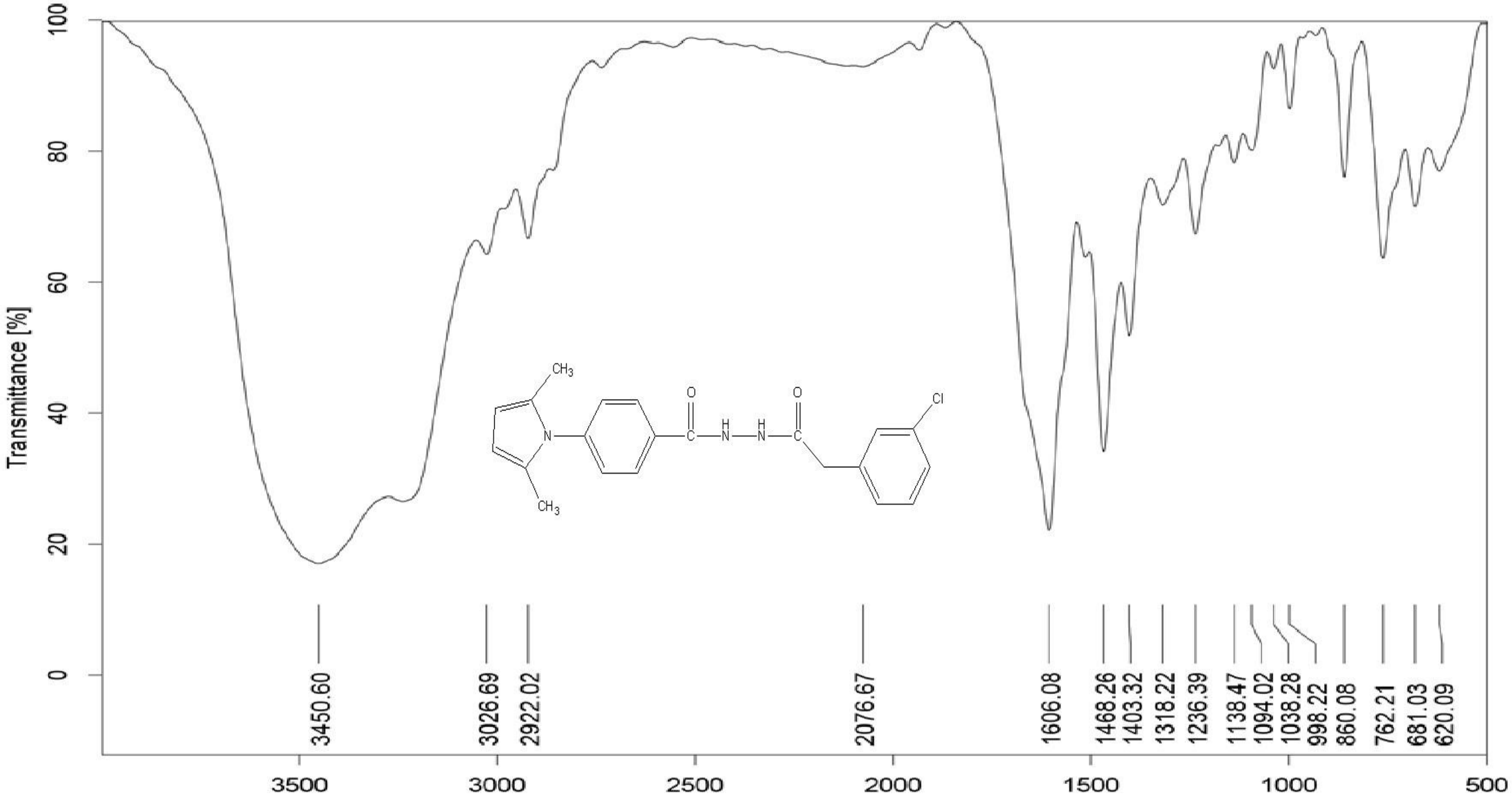

SPECTRUM NO: 30 (^{1}H NMR spectrum of Compound 5h)

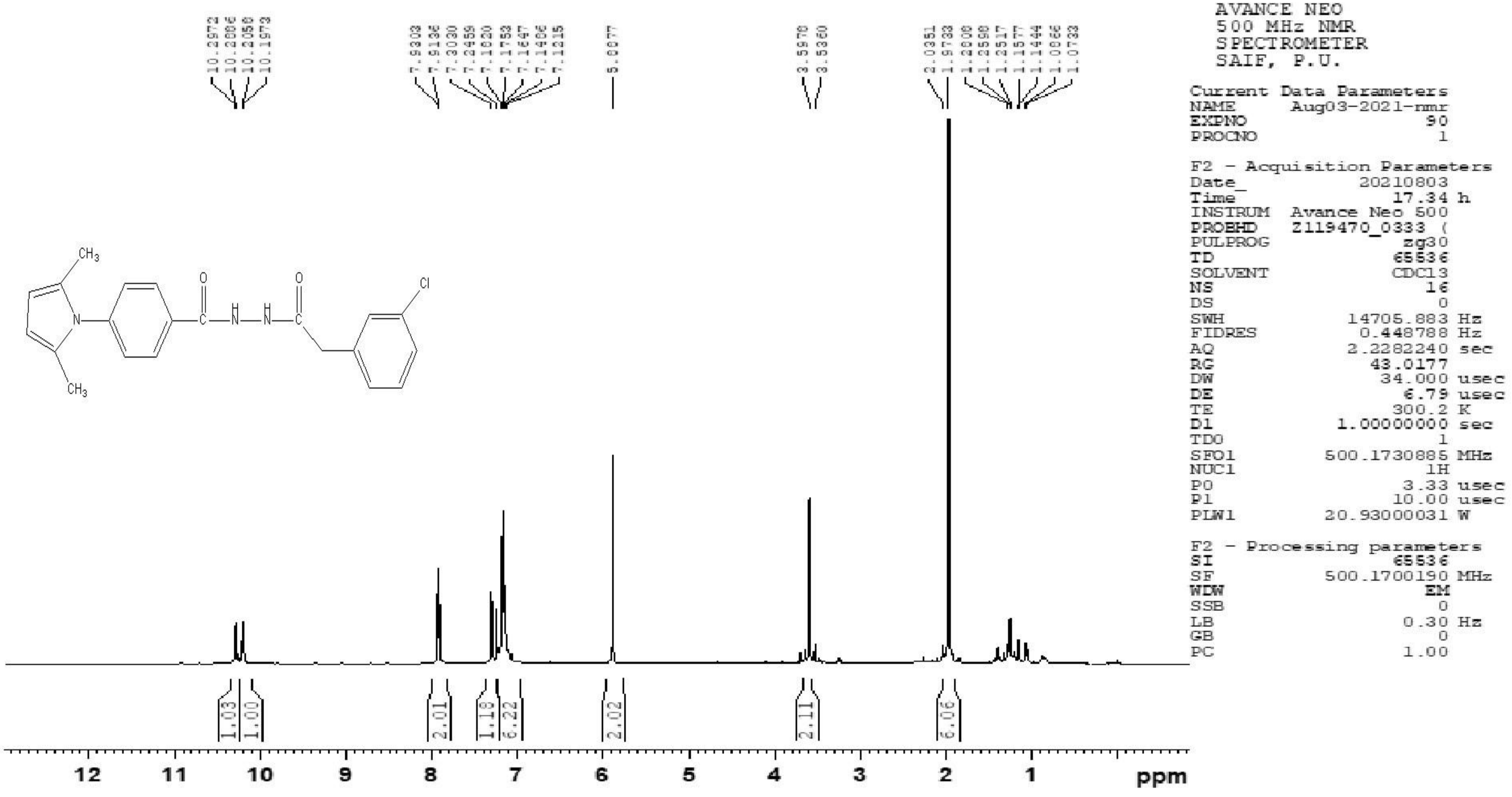

SPECTRUM NO: 31 (^{13}C NMR spectrum of Compound 5h)

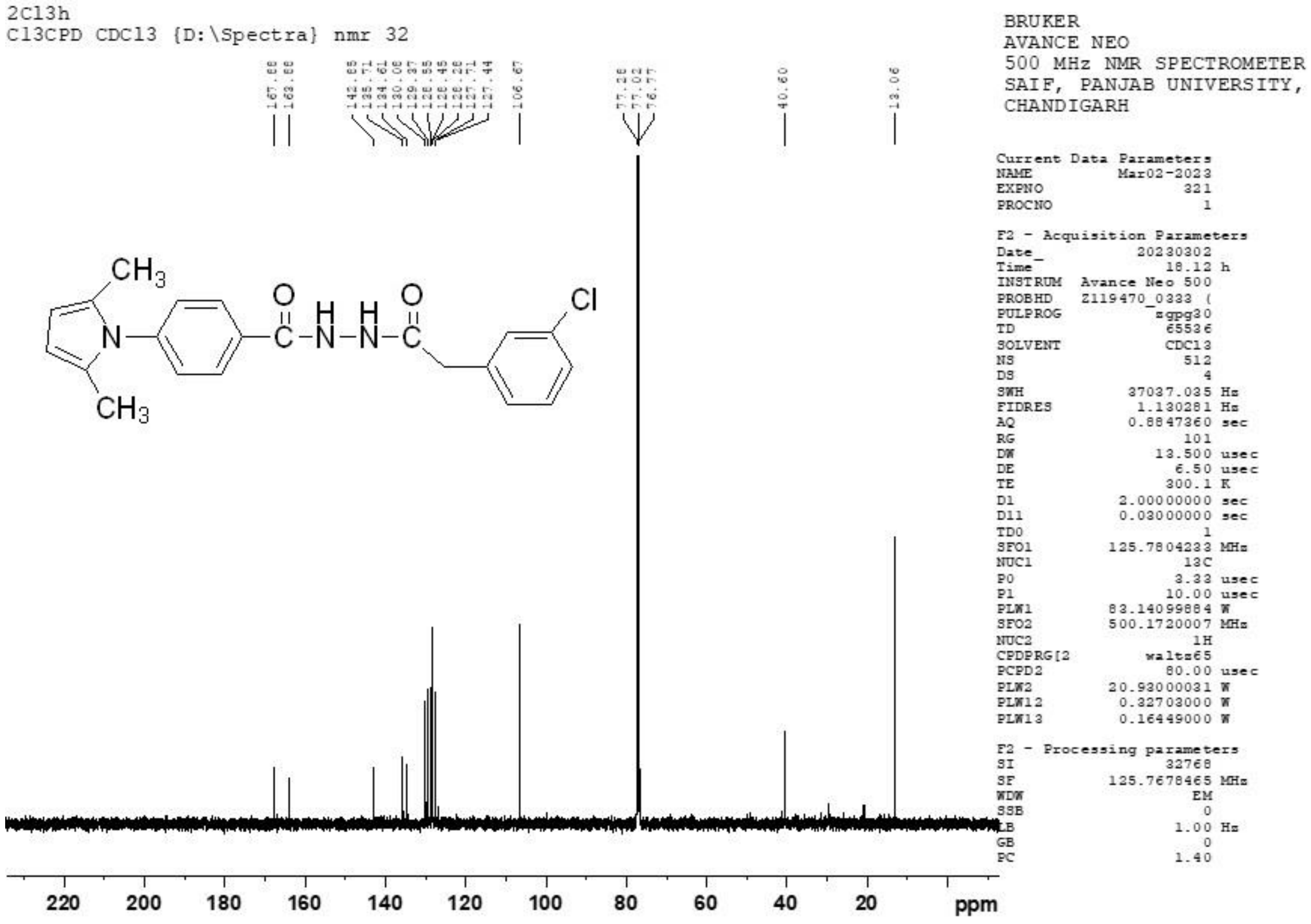

SPECTRUM NO: 32 (Mass spectrum of Compound 5h)

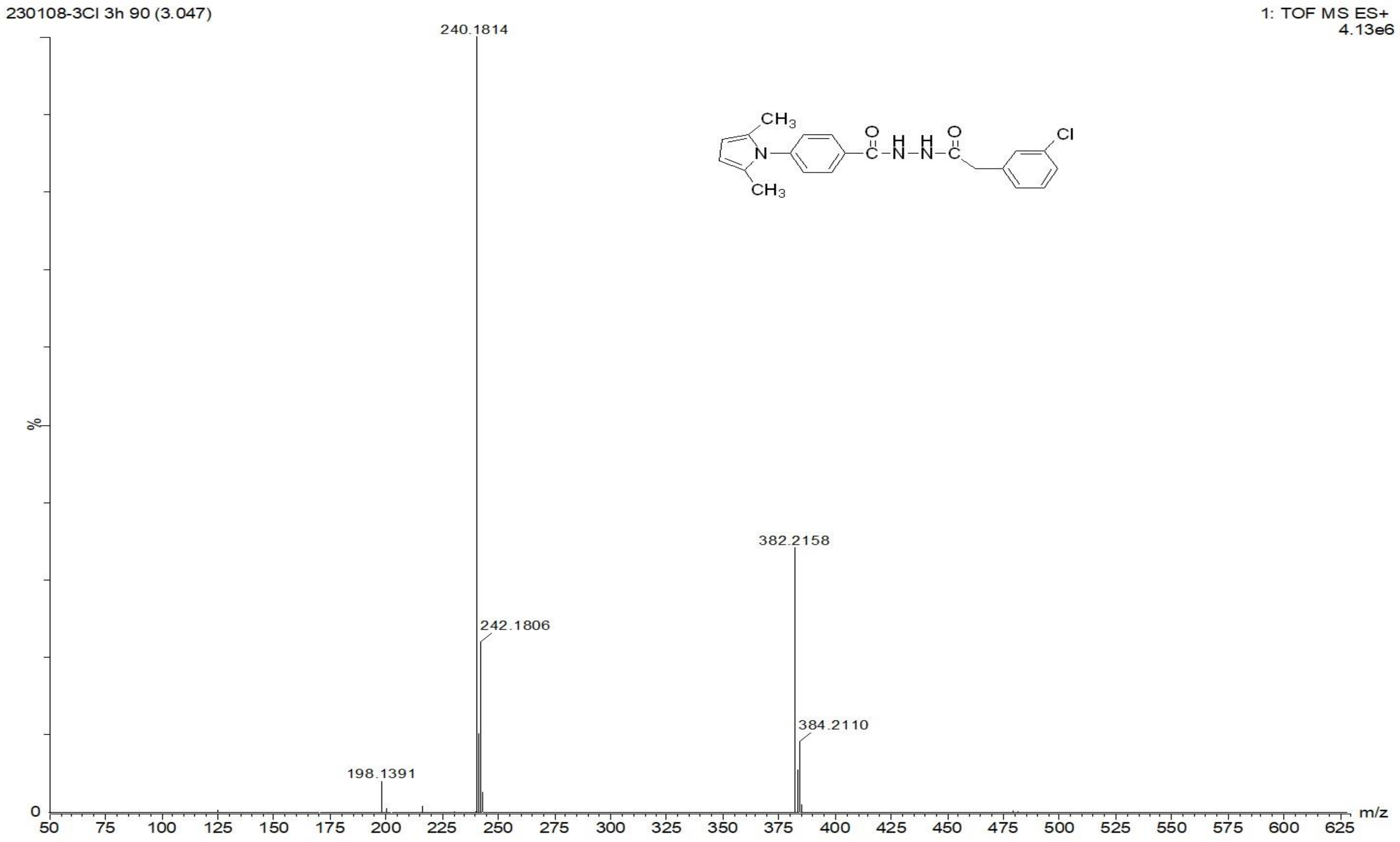

SPECTRUM NO: 33 (IR spectrum of Compound 5i)

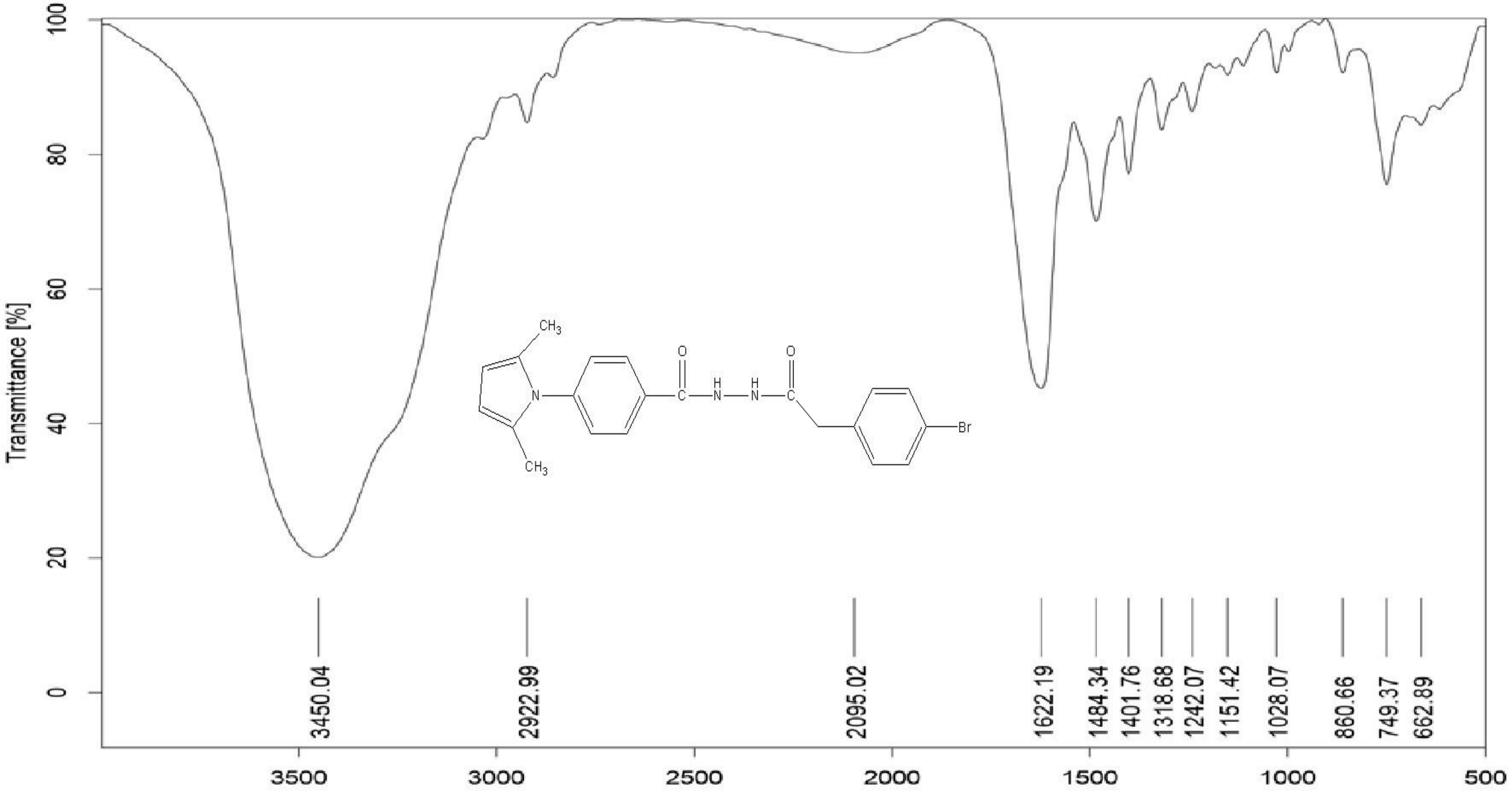

SPECTRUM NO: 34 (^{1}H NMR spectrum of Compound 5i)

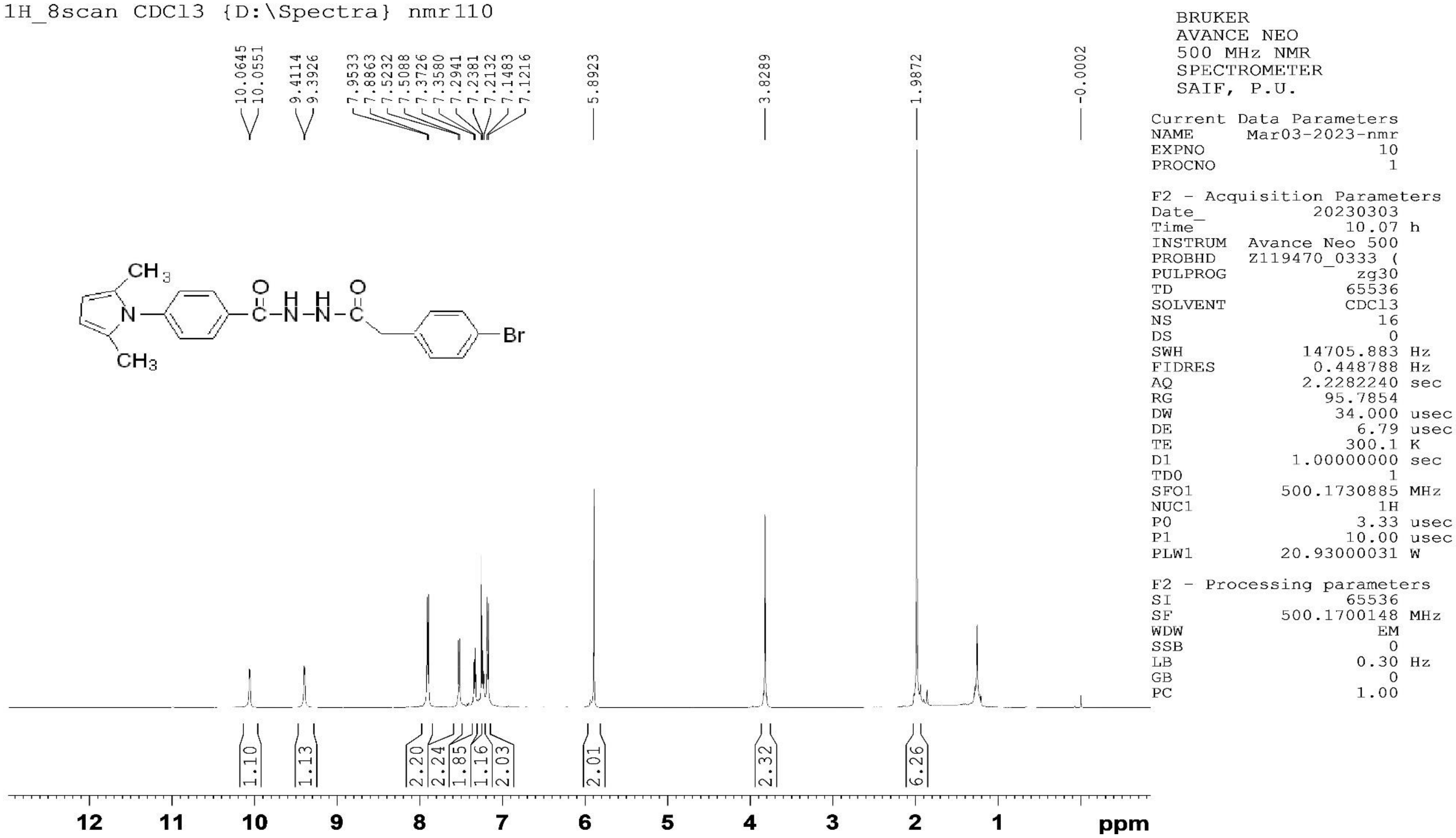

SPECTRUM NO: 35 (^{13}C NMR spectrum of Compound 5i)

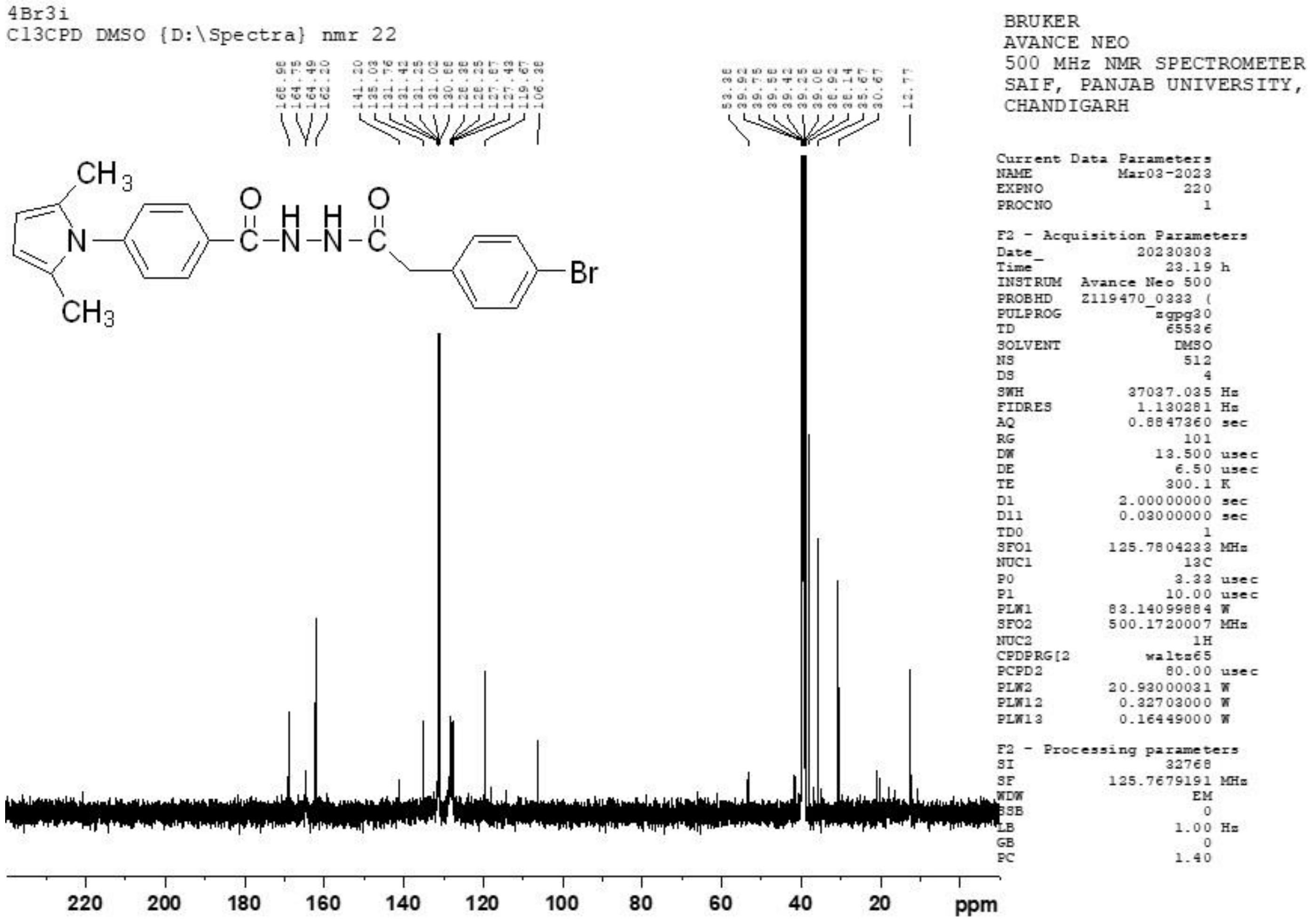

SPECTRUM NO: 36 (Mass spectrum of Compound 5i)

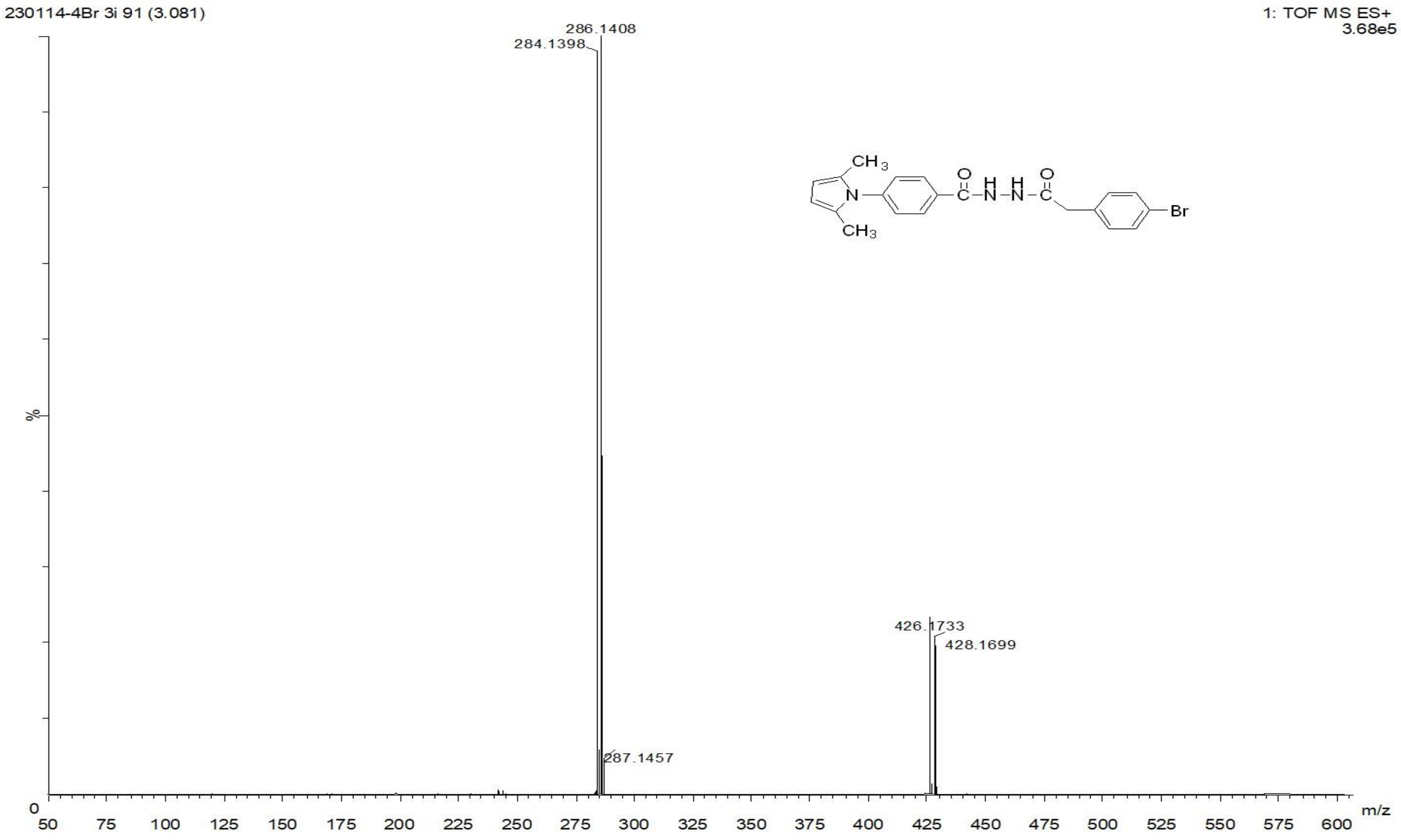

SPECTRUM NO: 37 (IR spectrum of Compound 5j)

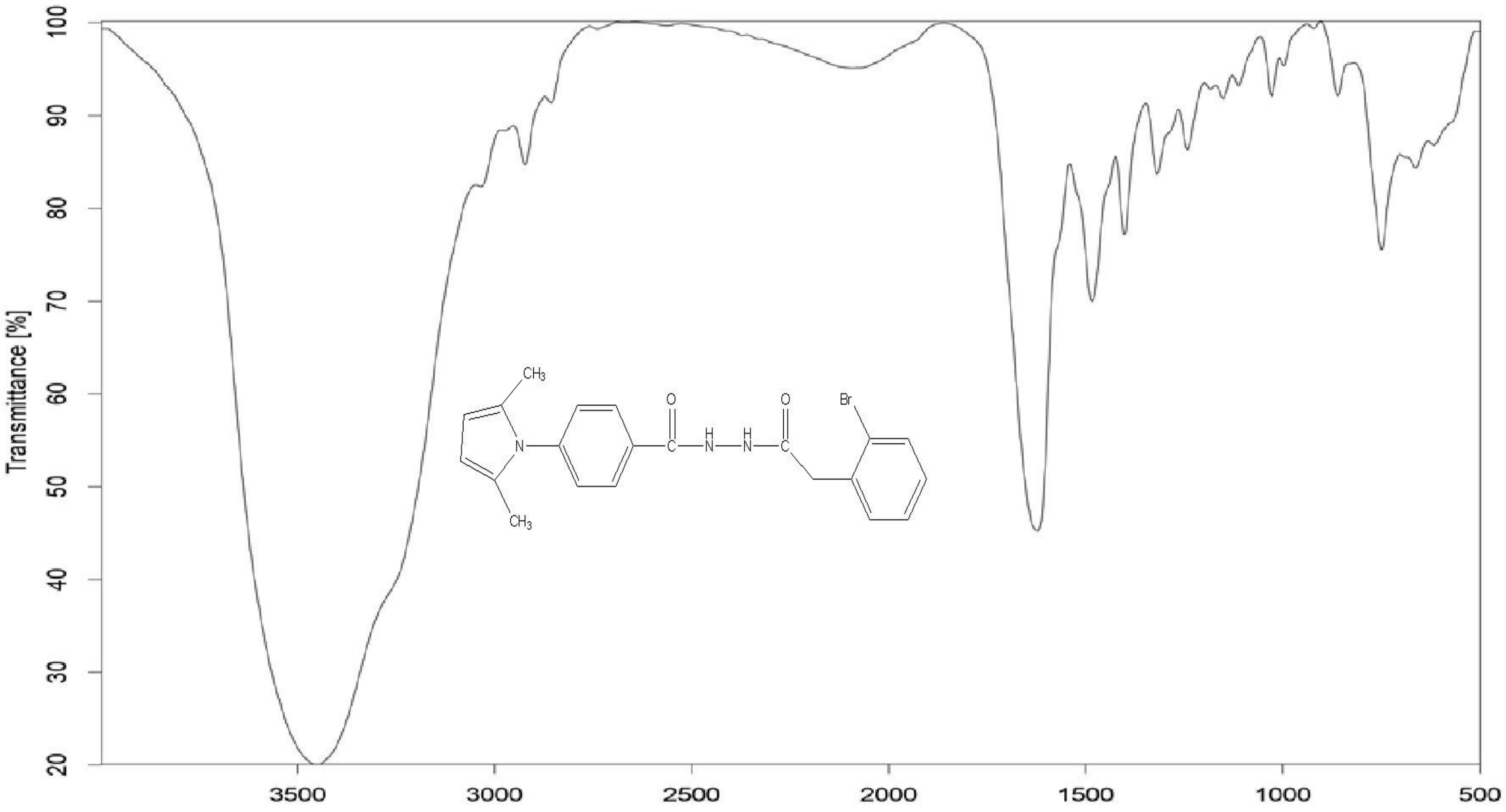

SPECTRUM NO: 38 (^{1}H NMR spectrum of Compound 5j)

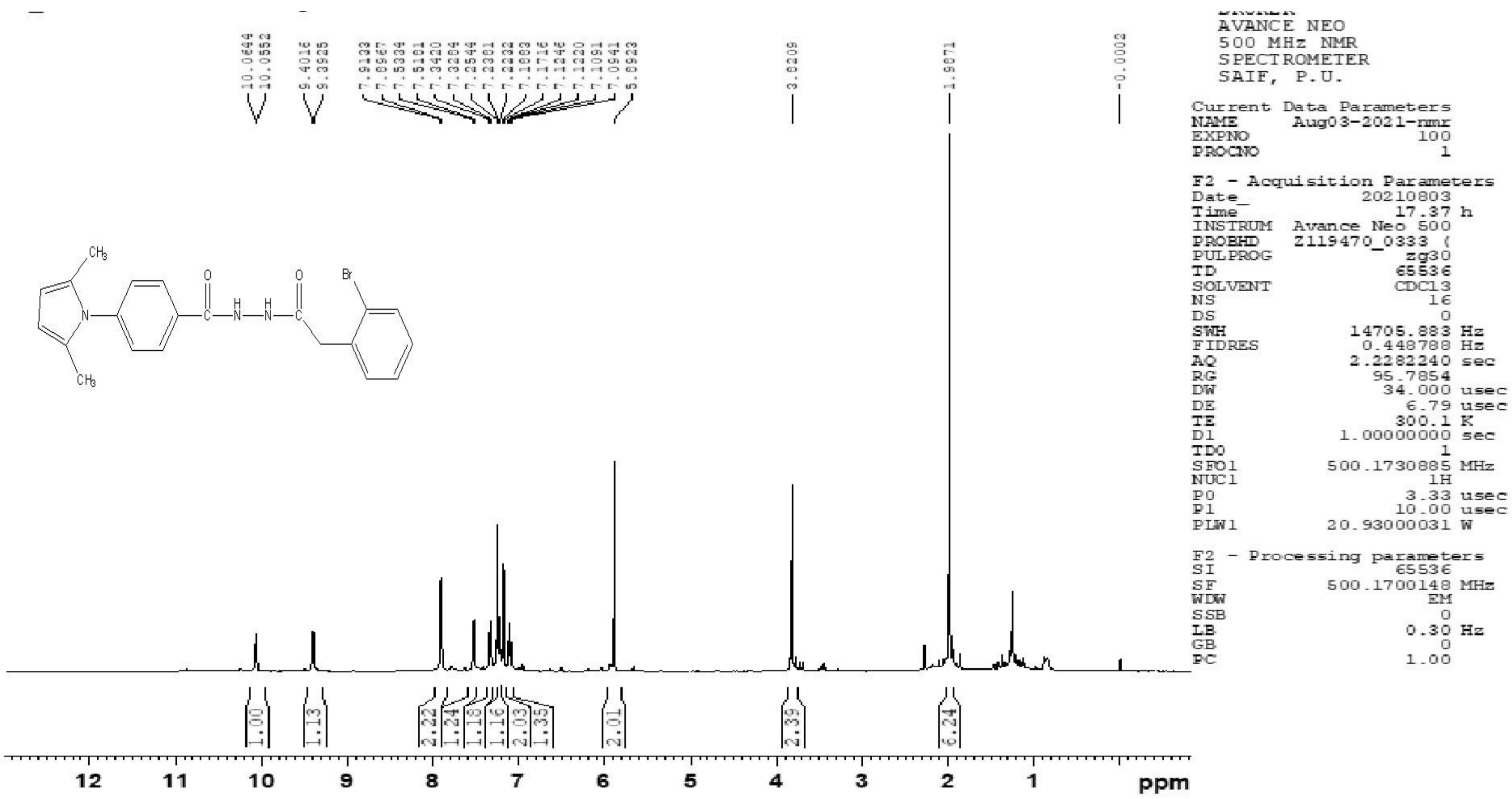

SPECTRUM NO: 39 (^{13}C NMR spectrum of Compound 5j)

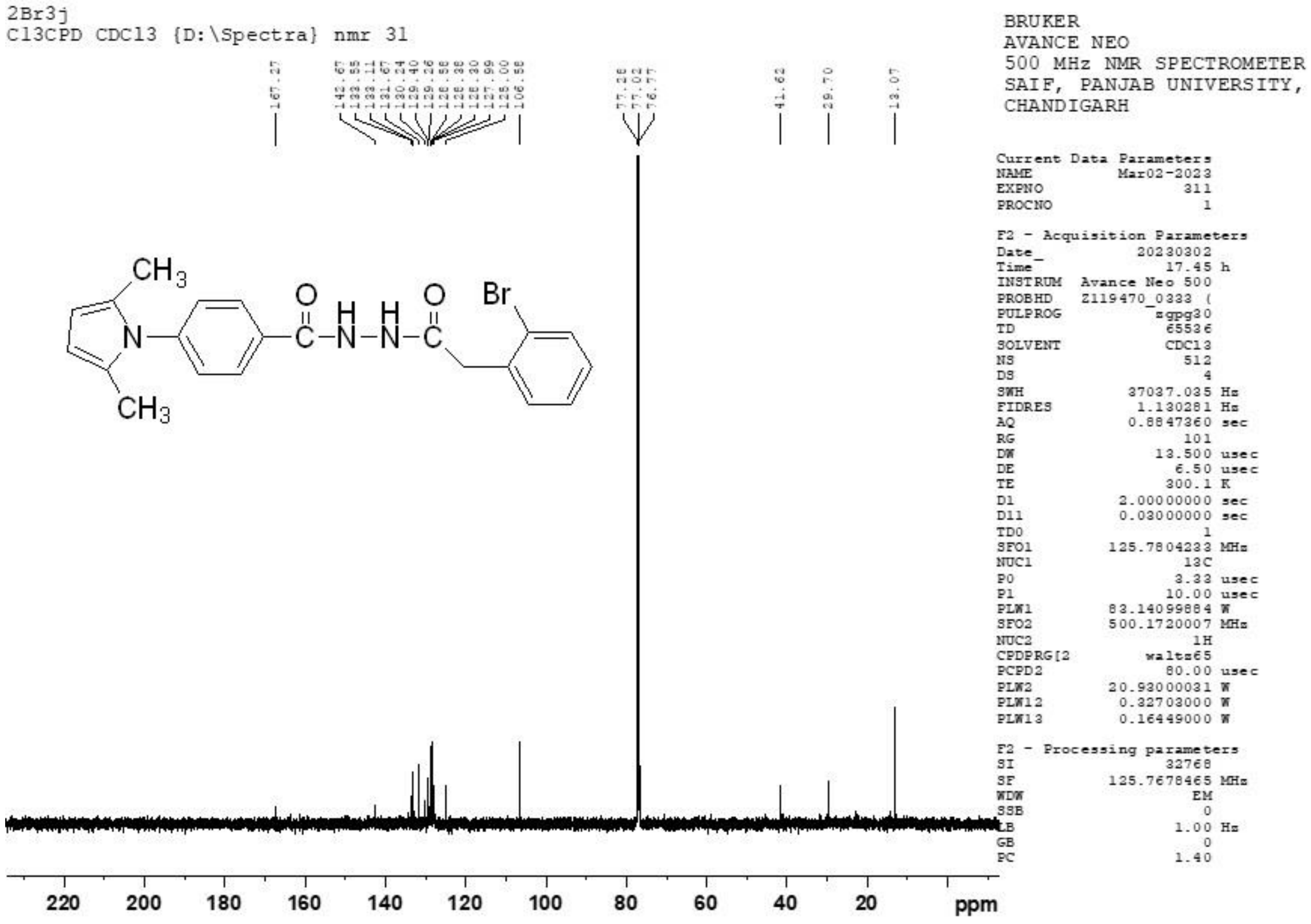

SPECTRUM NO: 40 (Mass spectrum of Compound 5j)

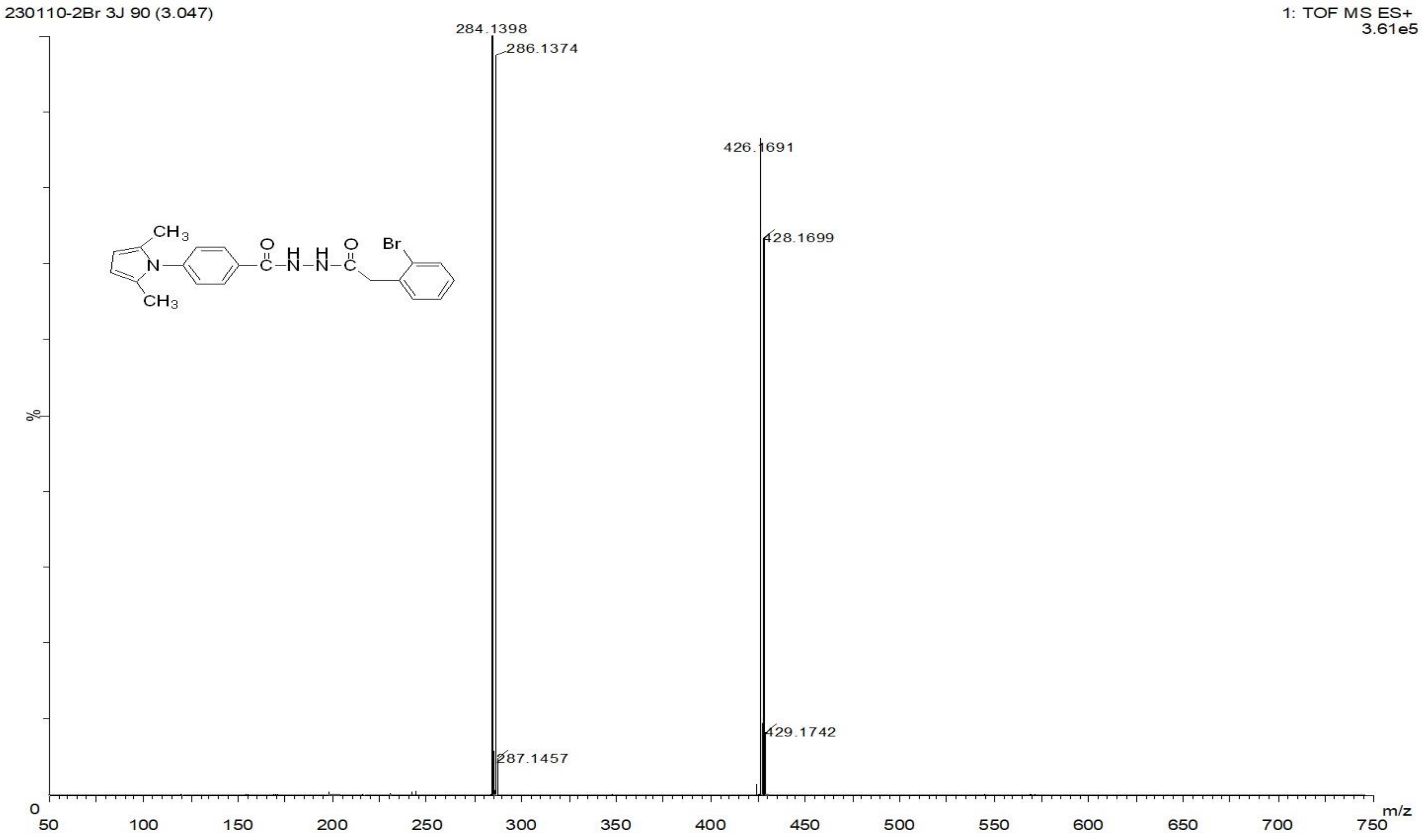

SPECTRUM NO: 41 (IR spectrum of Compound 5k)

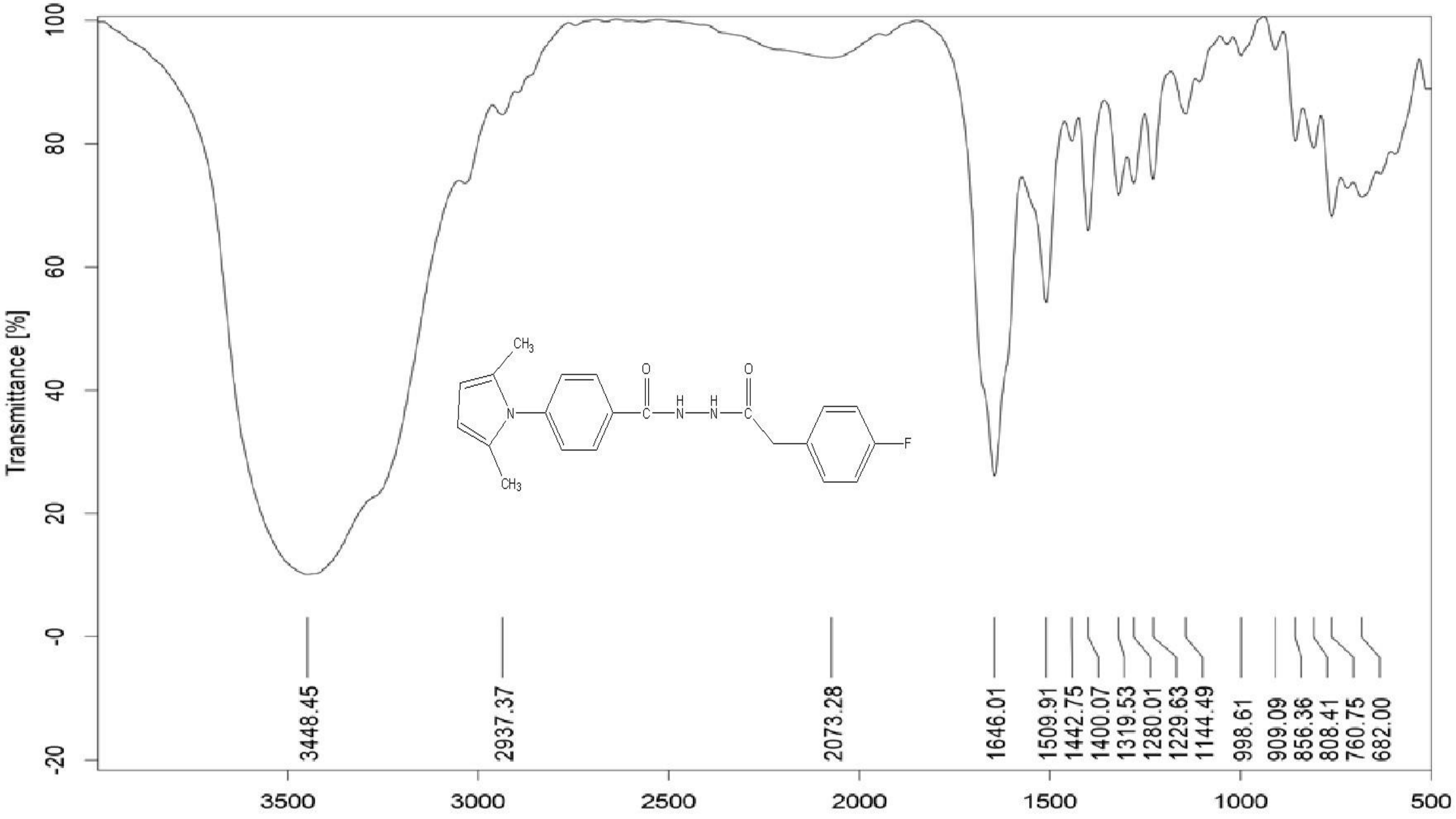

SPECTRUM NO: 42 (^{1}H NMR spectrum of Compound 5k)

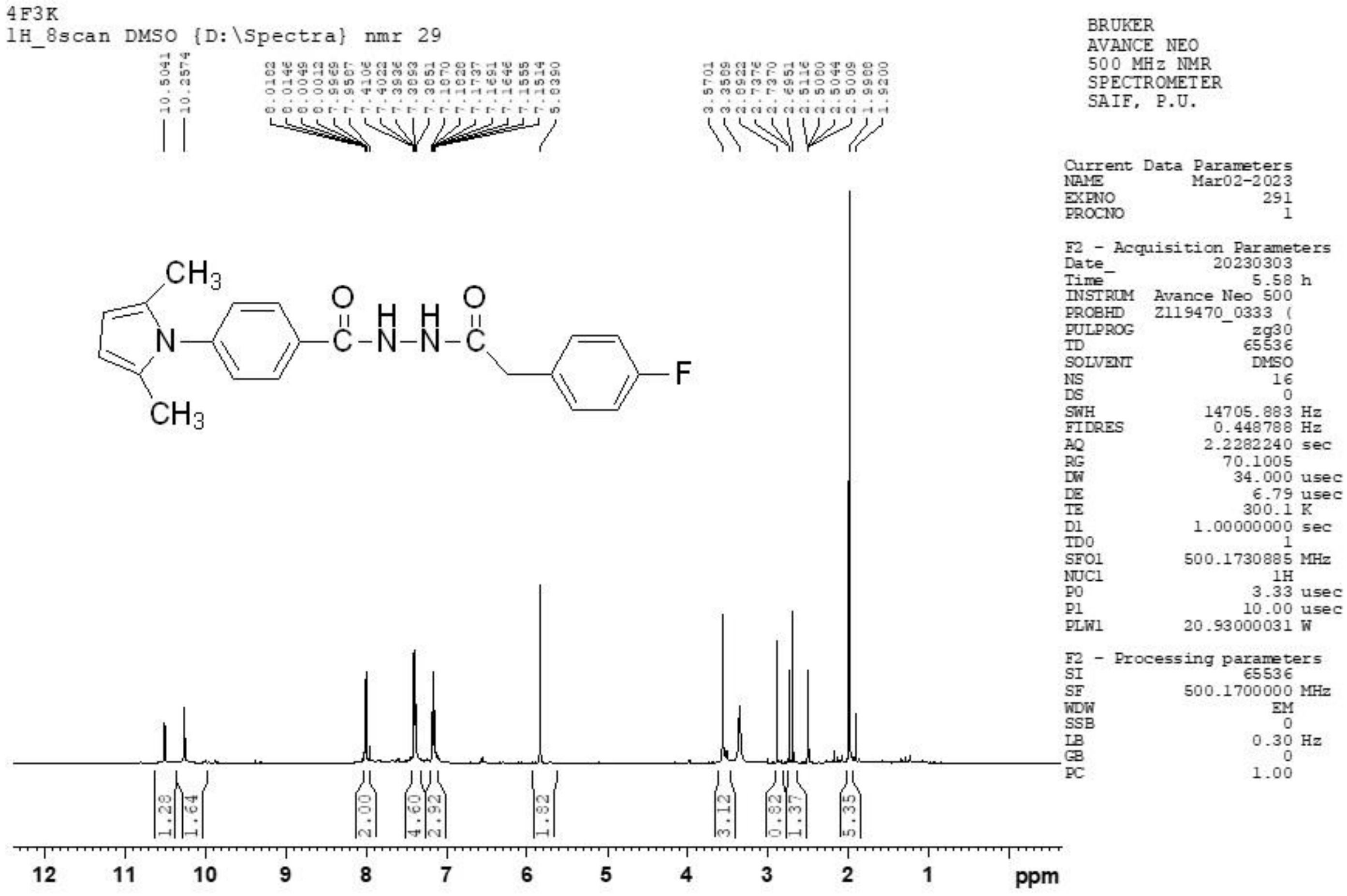

SPECTRUM NO: 43 (^{13}C NMR spectrum of Compound 5k)

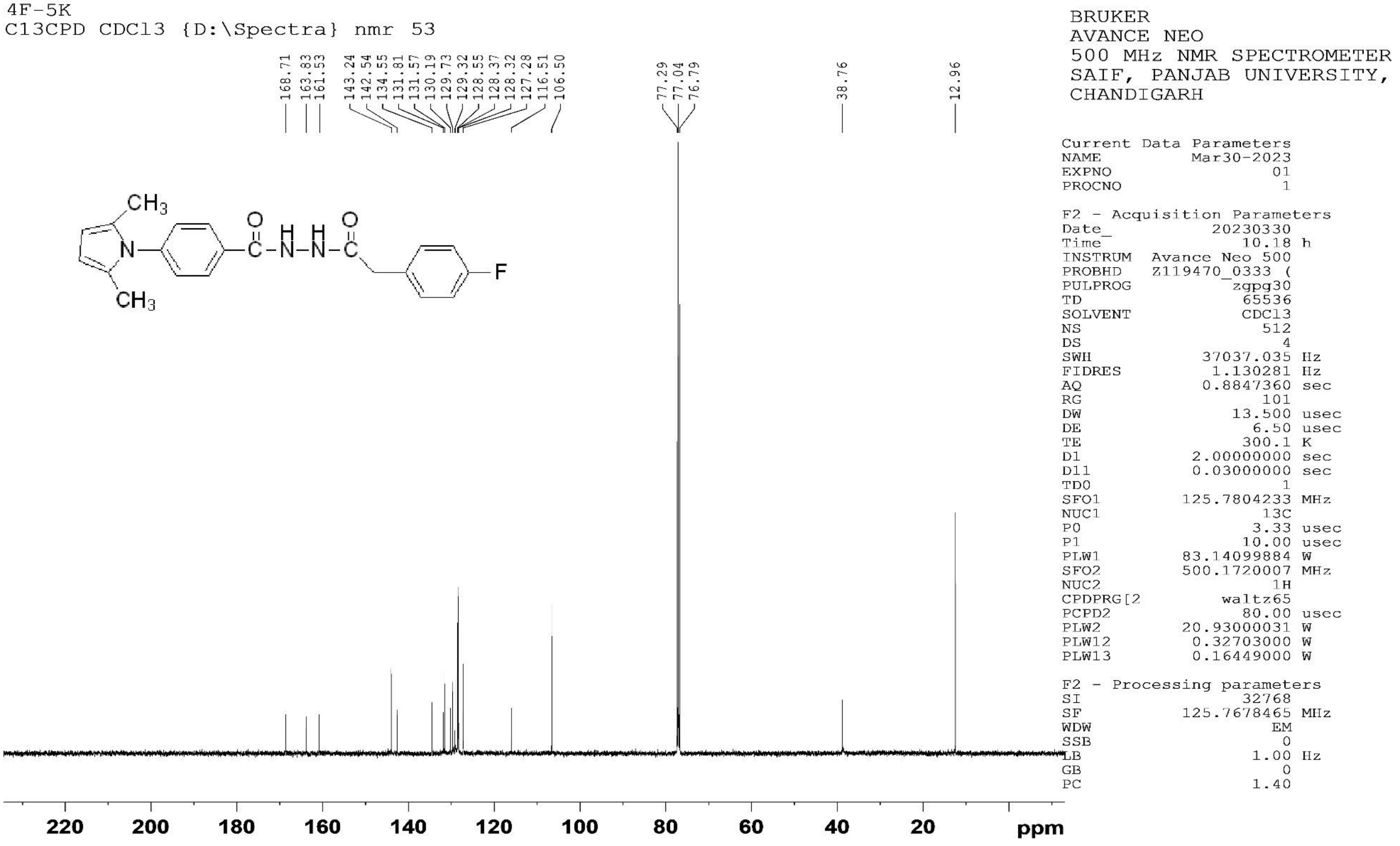

SPECTRUM NO: 44 (Mass spectrum of Compound 5k)

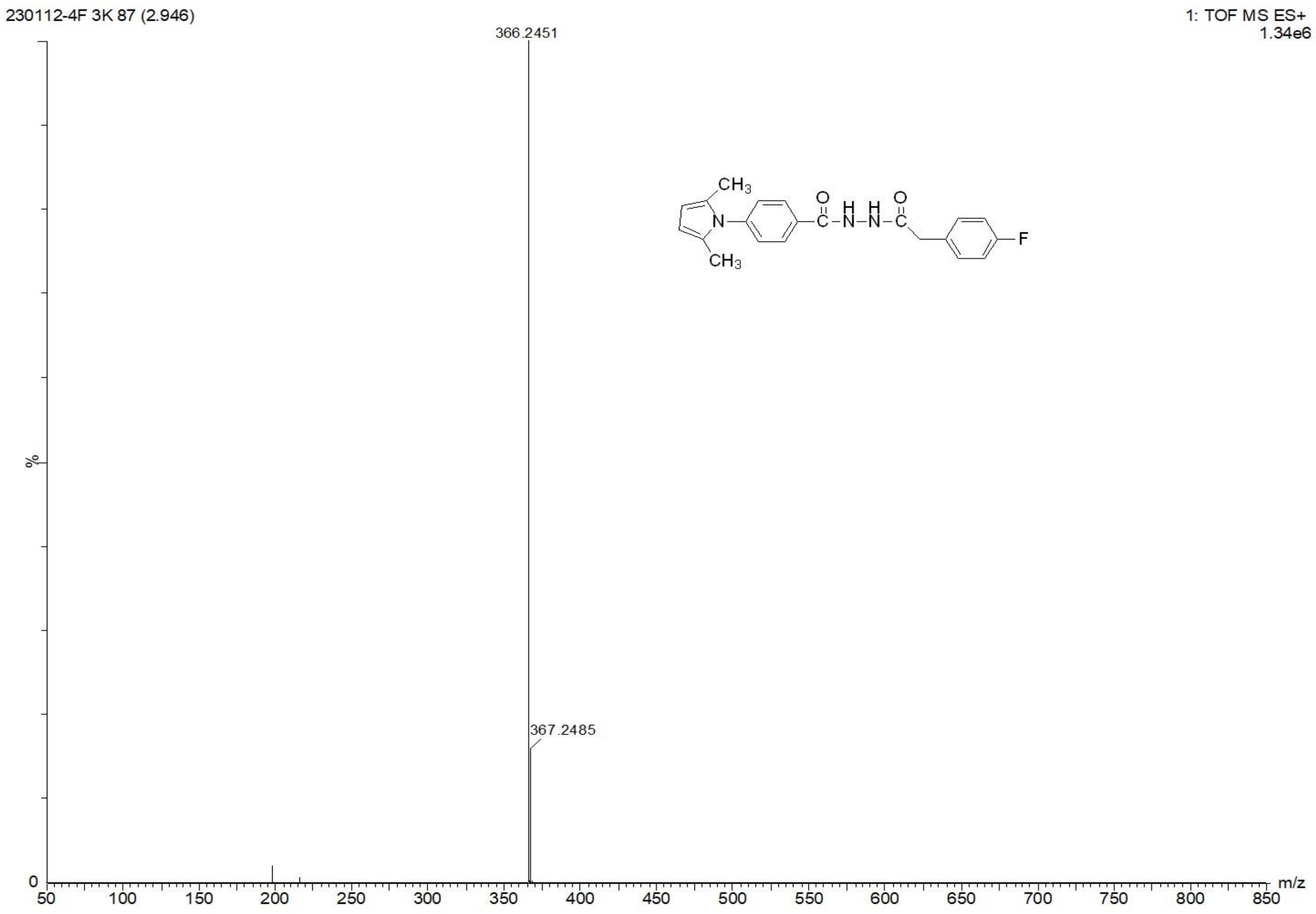

SPECTRUM NO: 45 (IR spectrum of Compound 5l)

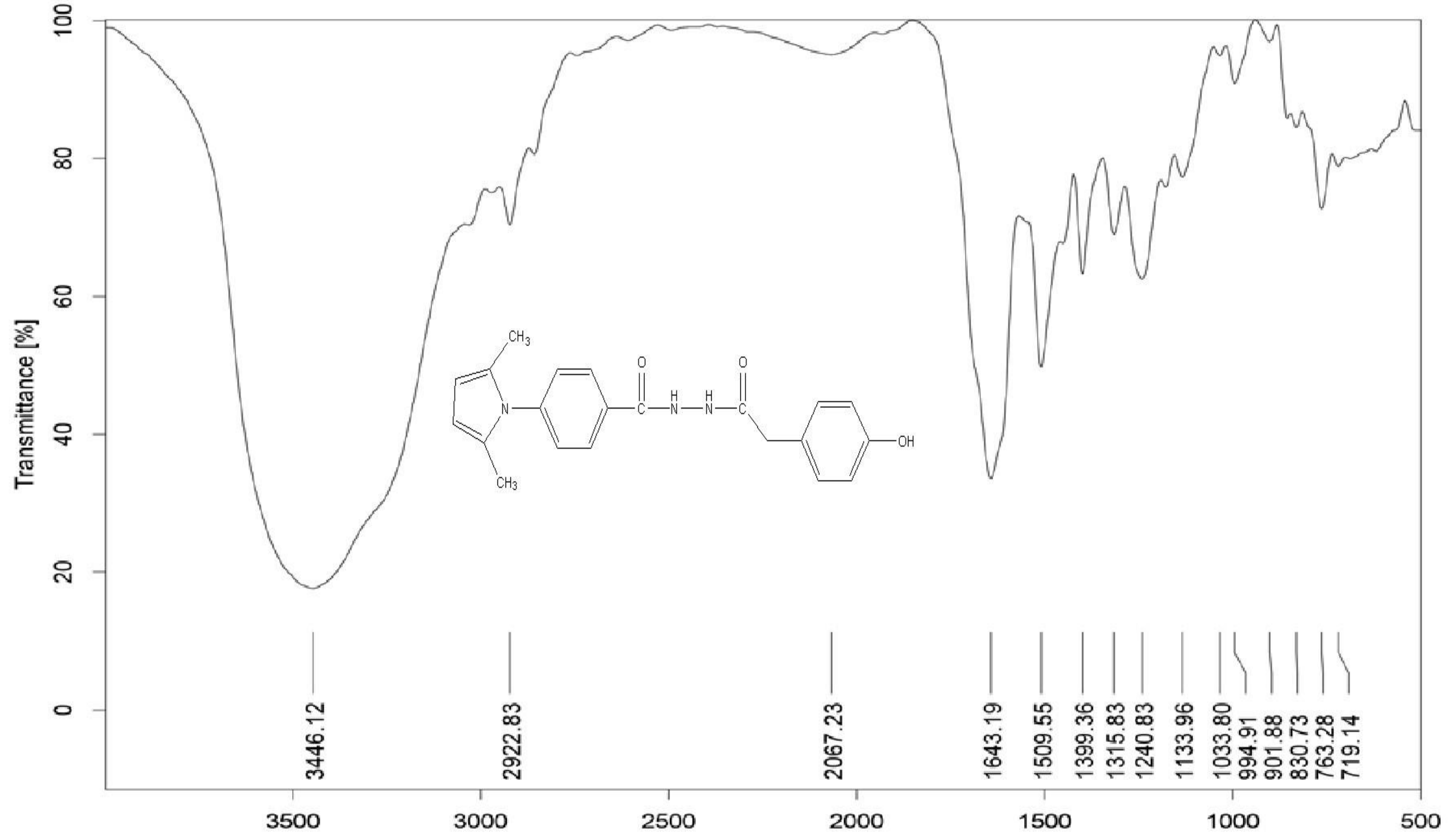

SPECTRUM NO: 46 (^{1}H NMR spectrum of Compound 5l)

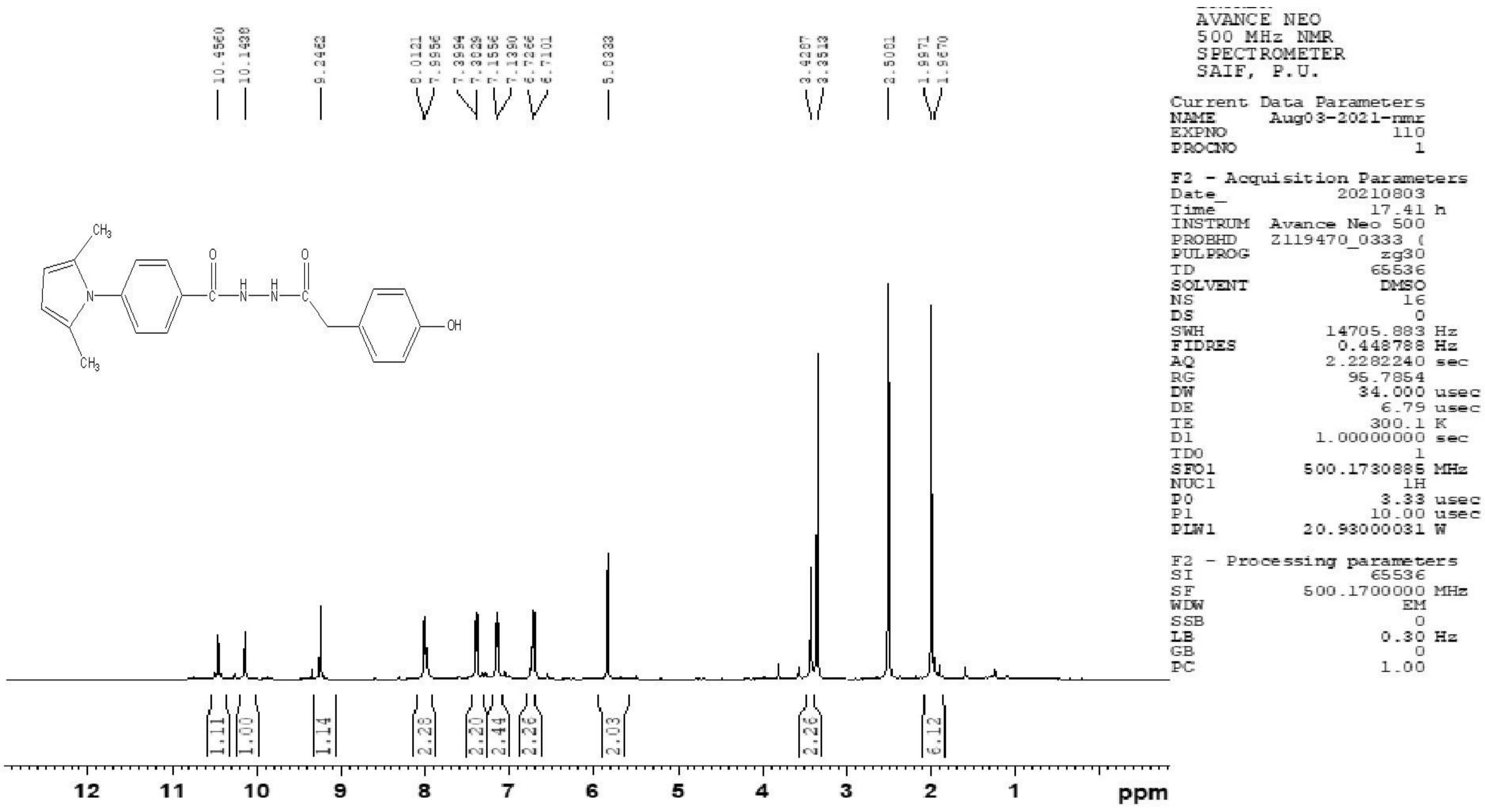

SPECTRUM NO: 47 (^{13}C NMR spectrum of Compound 5l)

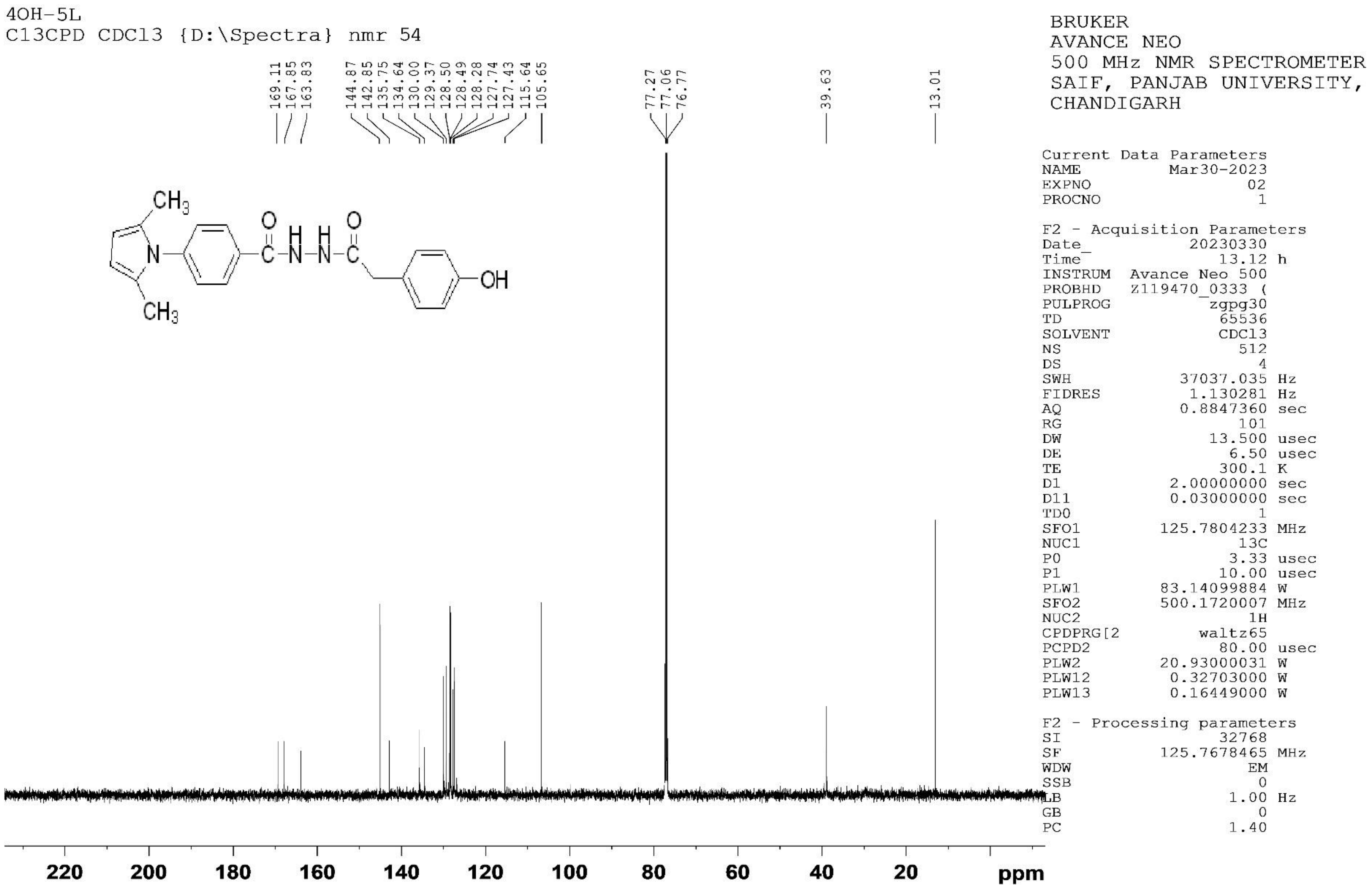

SPECTRUM NO: 48 (Mass spectrum of Compound 5l)

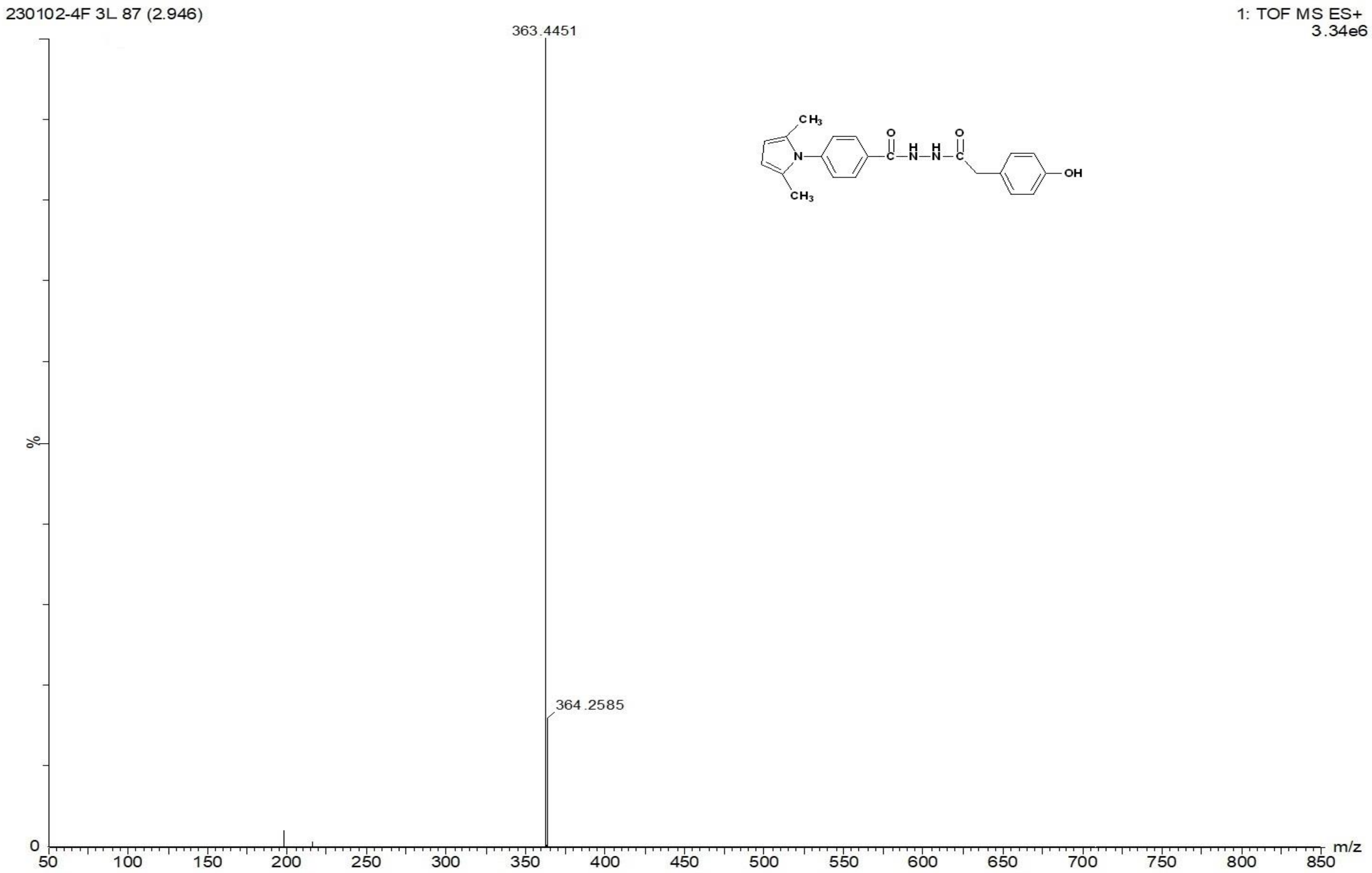

SPECTRUM NO: 49 (IR spectrum of Compound 5m)

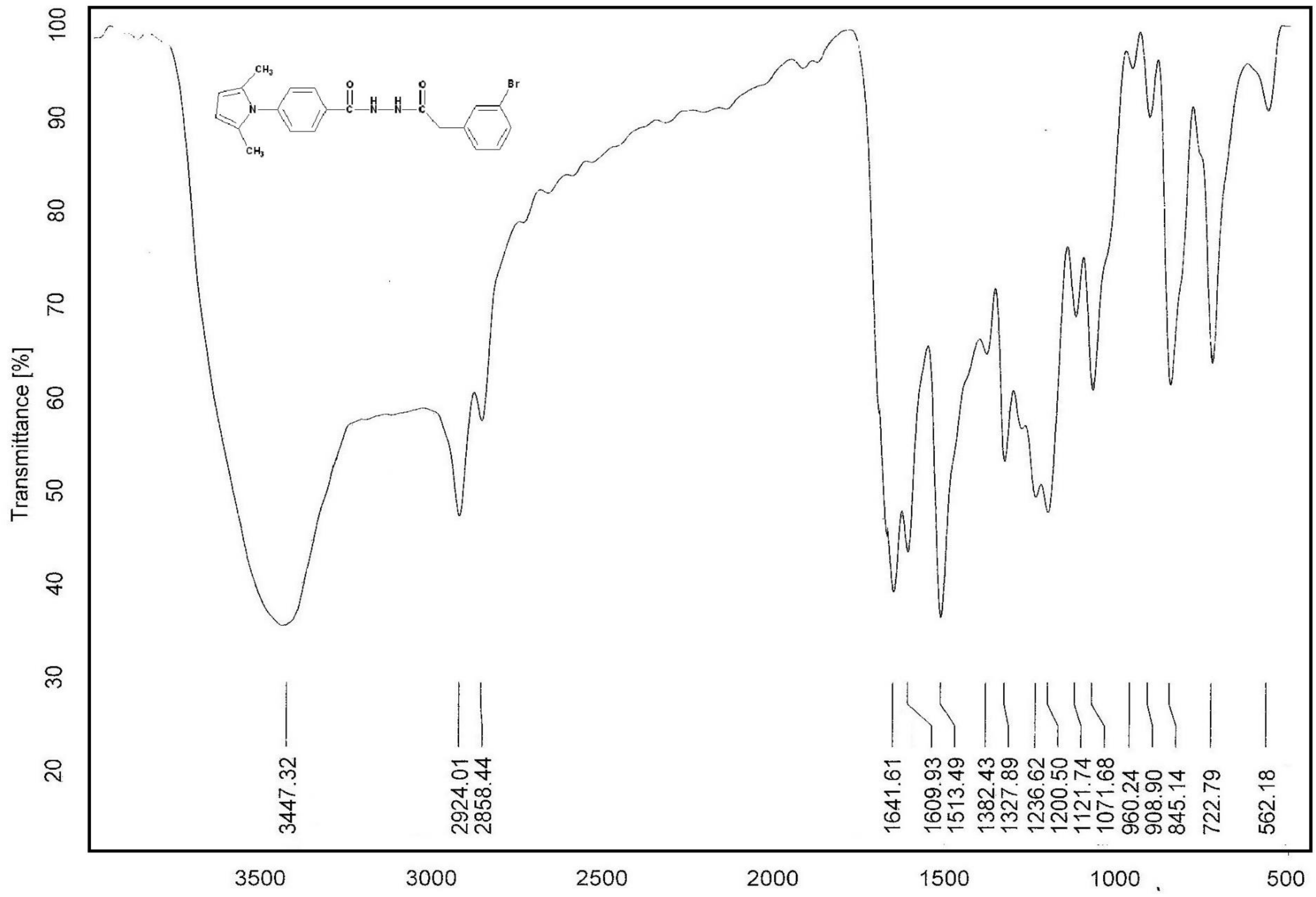

SPECTRUM NO: 50 (^{1}H NMR spectrum of Compound 5m)

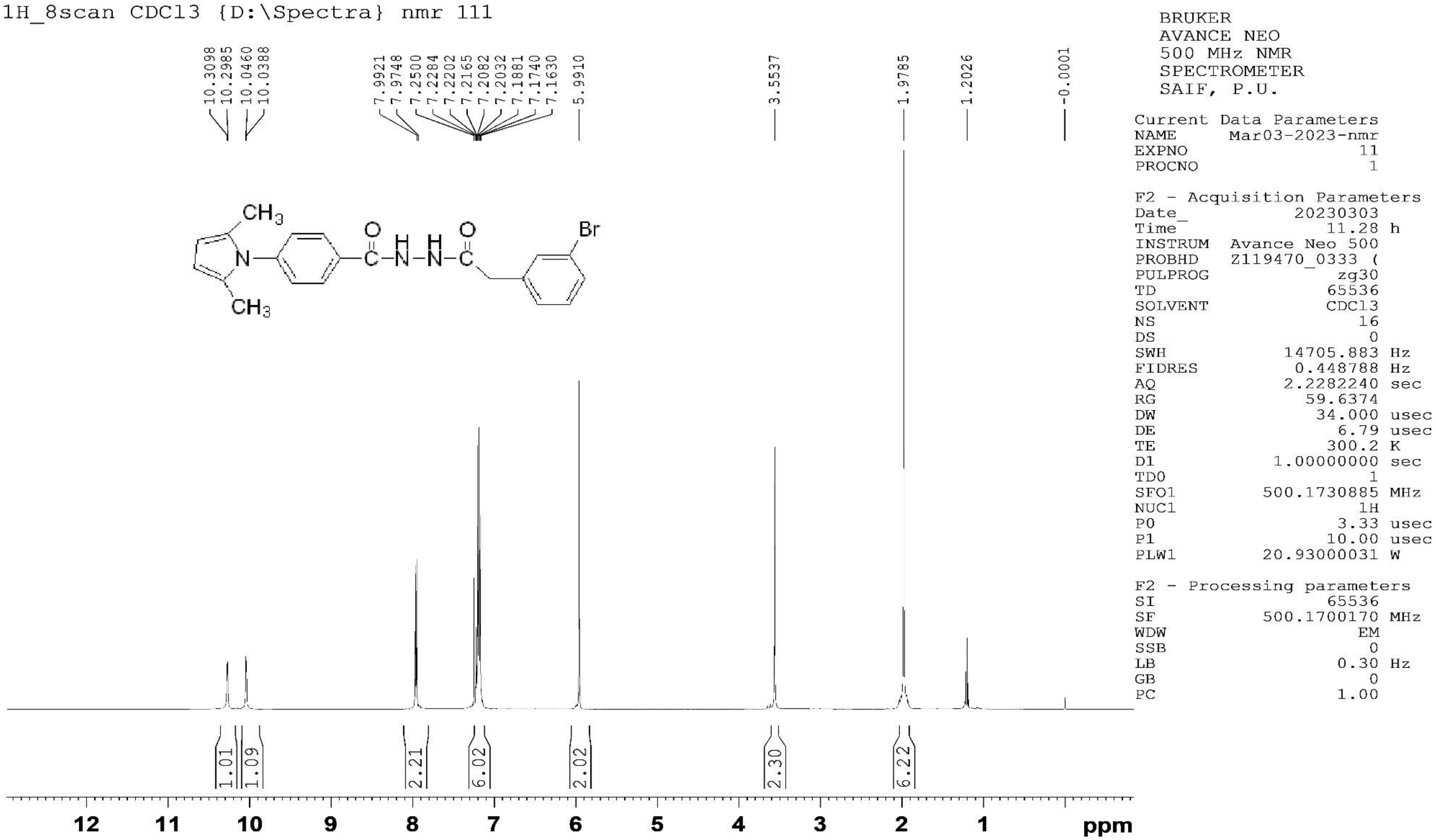

SPECTRUM NO: 51 (^{13}C NMR spectrum of Compound 5m)

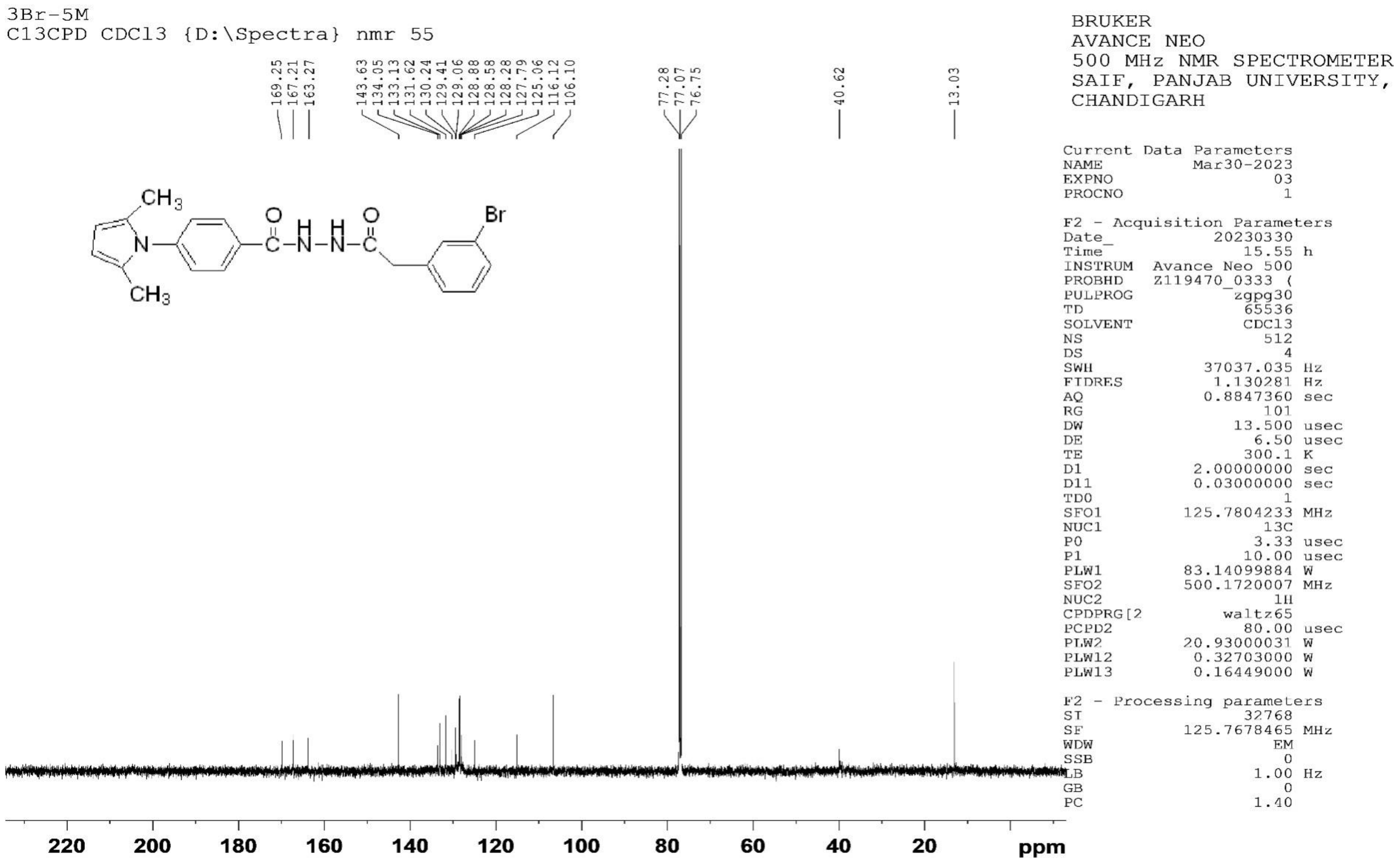

SPECTRUM NO: 52 (Mass spectrum of Compound 5m)

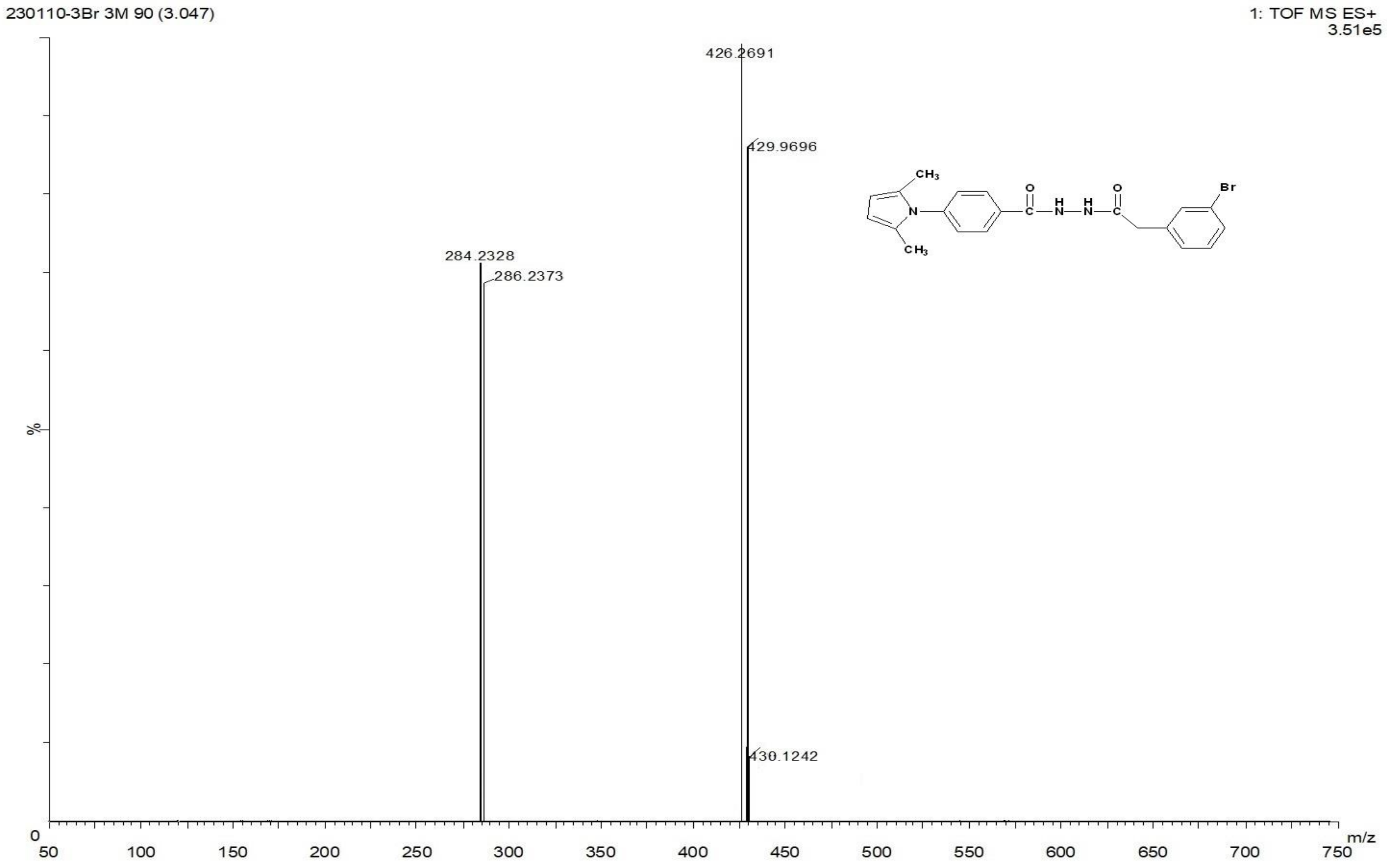

SPECTRUM NO: 53 (IR spectrum of Compound 5n)

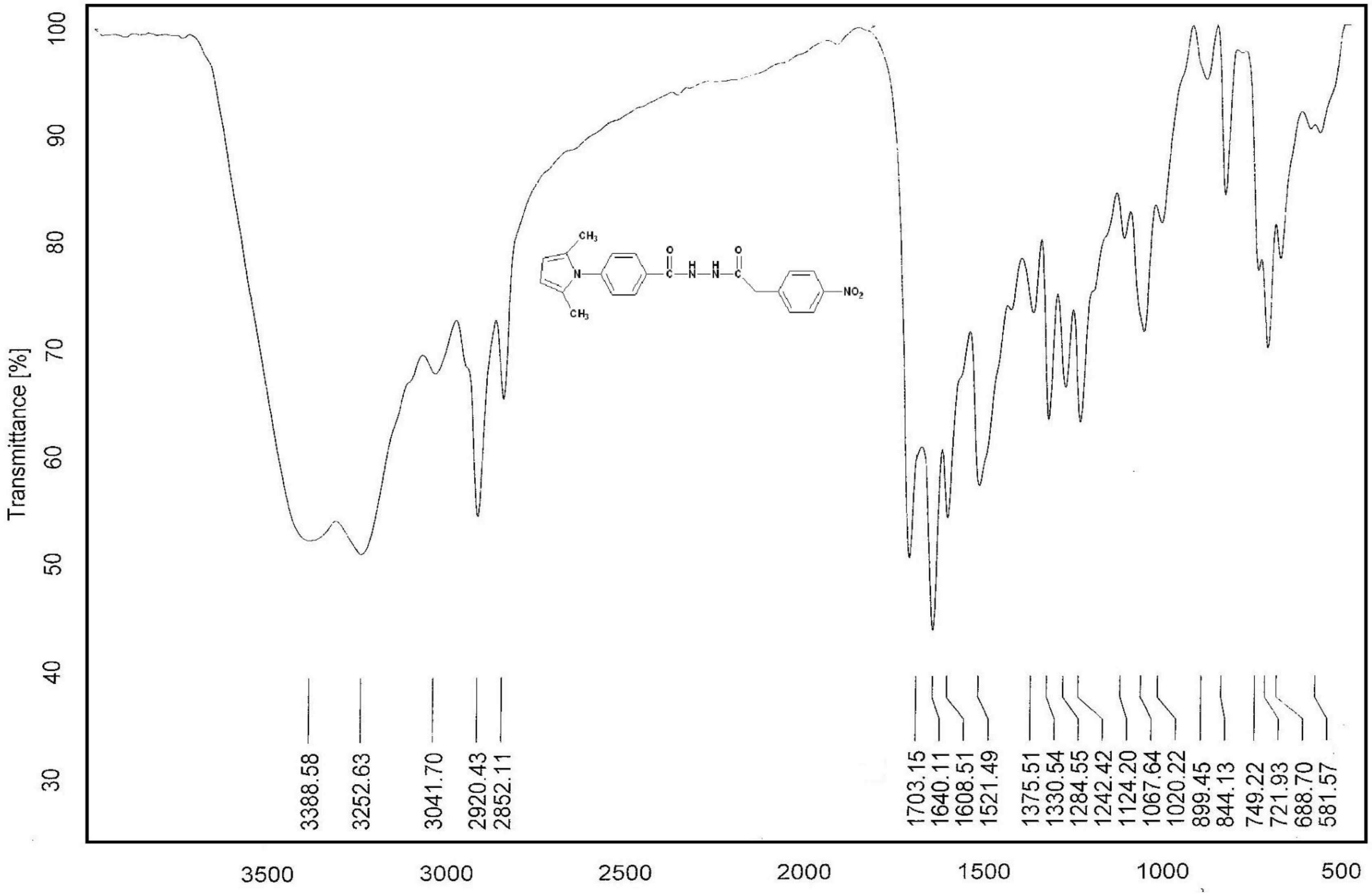

SPECTRUM NO: 54 (^{1}H NMR spectrum of Compound 5n)

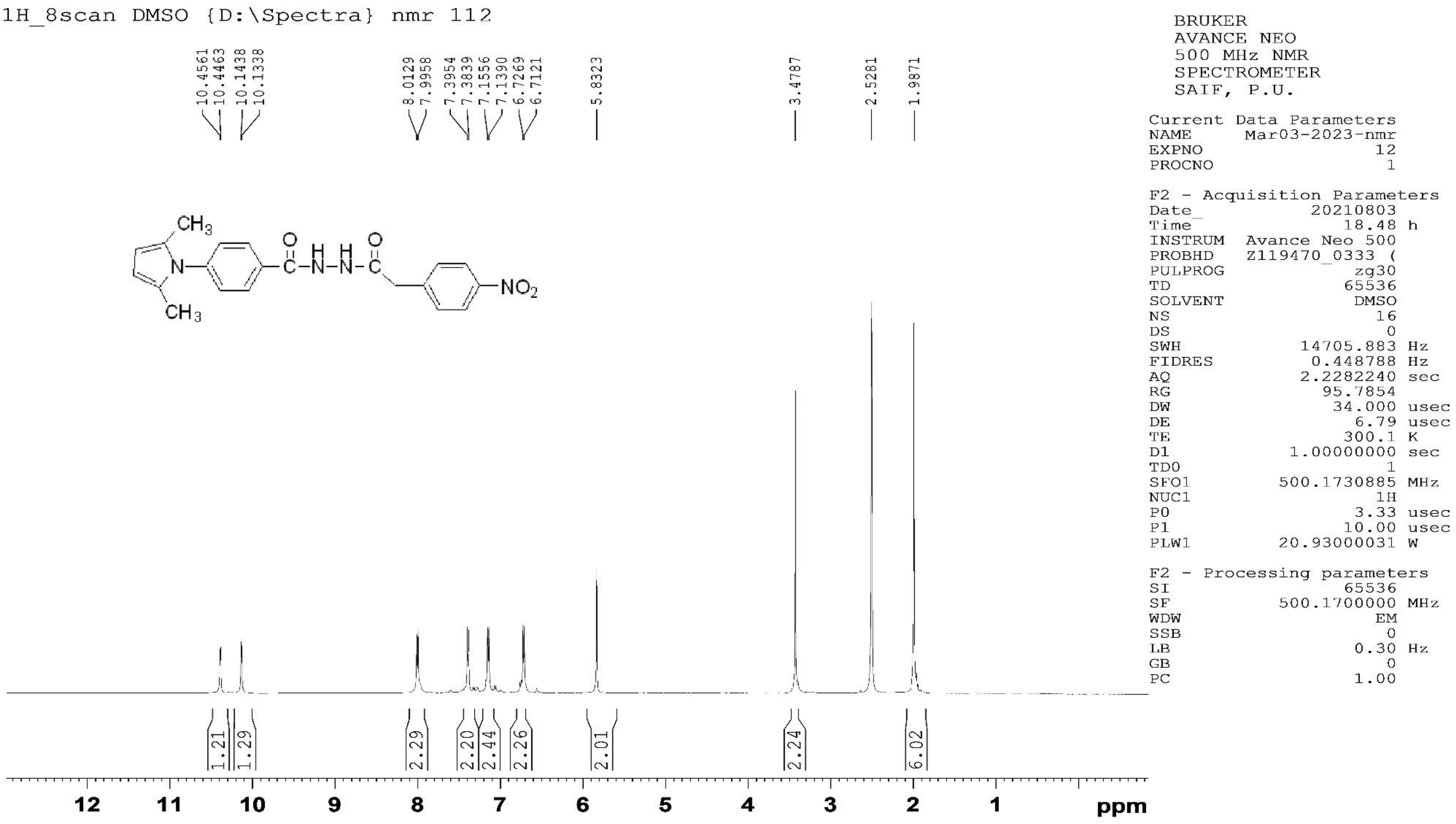

SPECTRUM NO: 55 (^{13}C NMR spectrum of Compound 5n)

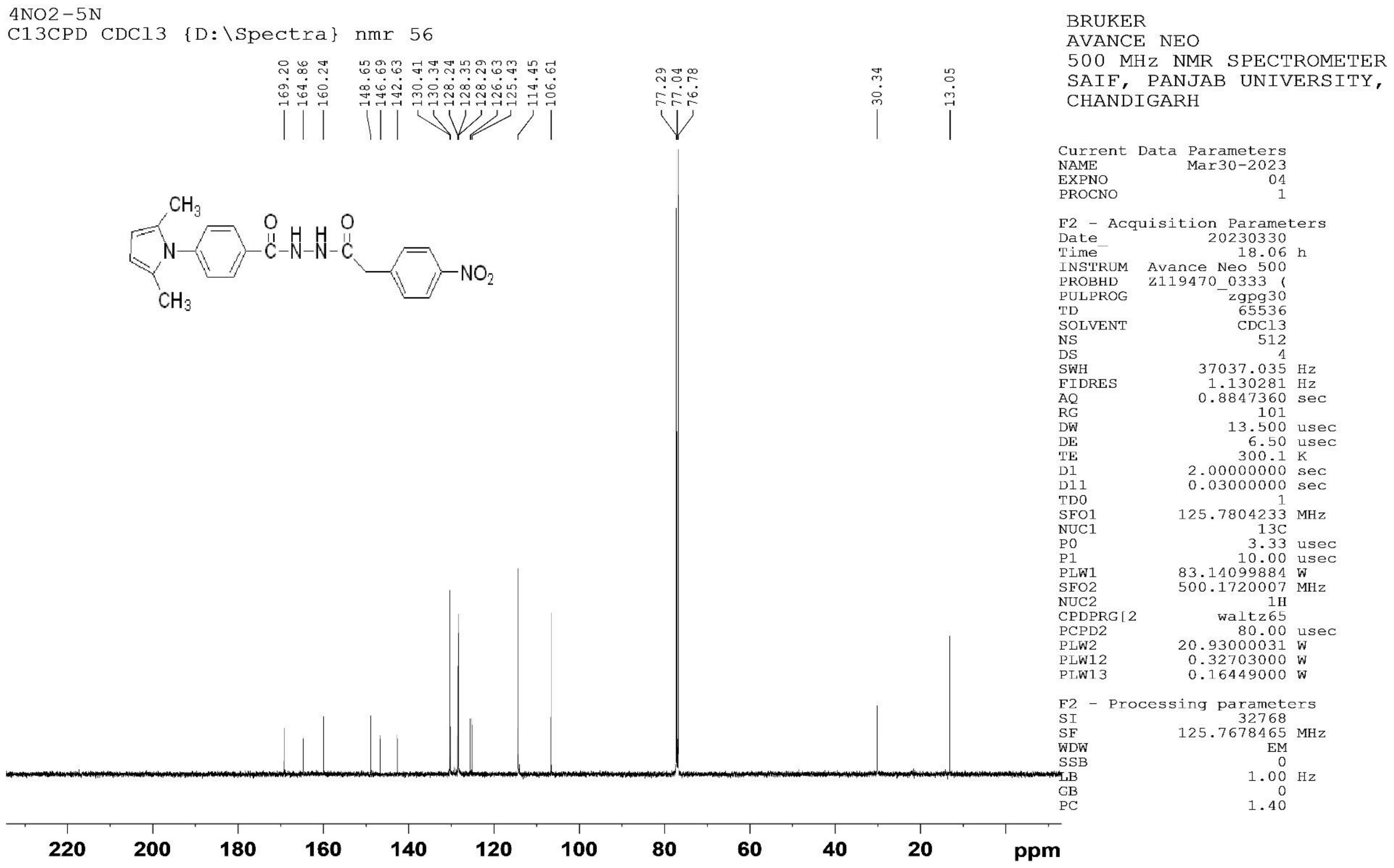

SPECTRUM NO: 56 (Mass spectrum of Compound 5n)

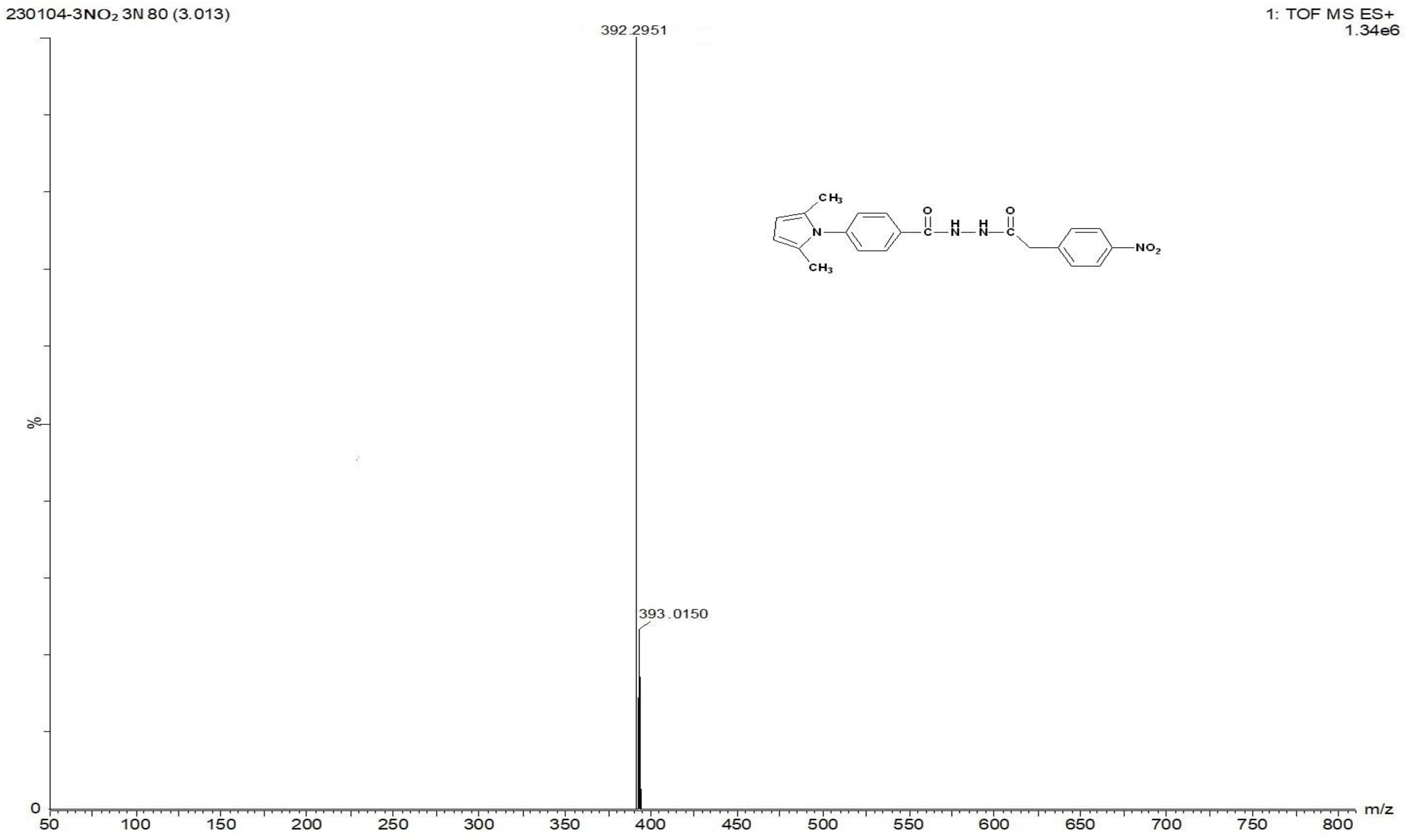

Supplement: Supplementary file 1 [file antibiotics-12-00763-s001.zip › antibiotics-2232440-supplementary.pdf]
